# The quorum quenching enzyme Aii20J modifies *in vitro* periodontal biofilm formation

**DOI:** 10.3389/fcimb.2023.1118630

**Published:** 2023-02-02

**Authors:** Ana Parga, Andrea Muras, Paz Otero-Casal, Alexandre Arredondo, Agnès Soler-Ollé, Gerard Àlvarez, Luis D. Alcaraz, Alex Mira, Vanessa Blanc, Ana Otero

**Affiliations:** ^1^ Department of Microbiology and Parasitology, CIBUS-Faculty of Biology, Universidade de Santiago de Compostela, Santiago de Compostela, Spain; ^2^ Department of Surgery and Medical-Surgical Specialties, Faculty of Medicine and Odontology, Universidade de Santiago de Compostela, Santiago de Compostela, Spain; ^3^ Unit of Oral Health, Santa Comba-Negreira, (CS) SERGAS, Santiago de Compostela, Spain; ^4^ Department of Microbiology, Dentaid Research Center, Cerdanyola Del Vallès, Spain; ^5^ Department of Cellular Biology, Faculty of Sciences, National Autonomous University of Mexico, Coyoacán, Mexico; ^6^ Department of Genomics and Health, FISABIO Foundation, Valencia, Spain

**Keywords:** acyl-homoserine lactones, quorum quenching, oral biofilm, multispecies biofilm, antibiofilm strategies, oral metagenomes, bacterial diversity, periodontal disease

## Abstract

**Introduction:**

Recent studies have revealed the presence of *N*-acyl-homoserine lactones (AHLs) quorum sensing (QS) signals in the oral environment. Yet, their role in oral biofilm development remains scarcely investigated. The use of quorum quenching (QQ) strategies targeting AHLs has been described as efficient for the control of pathogenic biofilms. Here, we evaluate the use of a highly active AHL-targeting QQ enzyme, Aii20J, to modulate oral biofilm formation *in vitro*.

**Methods:**

The effect of the QQ enzyme was studied in *in vitro* multispecies biofilms generated from oral samples taken from healthy donors and patients with periodontal disease. Subgingival samples were used as inocula, aiming to select members of the microbiota of the periodontal pocket niche in the *in vitro* biofilms. Biofilm formation abilities and microbial composition were studied upon treating the biofilms with the QQ enzyme Aii20J.

**Results and Discussion:**

The addition of the enzyme resulted in significant biofilm mass reductions in 30 – 60% of the subgingival-derived biofilms, although standard AHLs could not be found in the supernatants of the cultured biofilms. Changes in biofilm mass were not accompanied by significant alterations of bacterial relative abundance at the genus level. The investigation of 125 oral supragingival metagenomes and a synthetic subgingival metagenome revealed a surprisingly high abundance and broad distribution of homologous of the AHL synthase HdtS and several protein families of AHL receptors, as well as an enormous presence of QQ enzymes, pointing to the existence of an intricate signaling network in oral biofilms that has been so far unreported, and should be further investigated. Together, our findings support the use of Aii20J to modulate polymicrobial biofilm formation without changing the microbiome structure of the biofilm. Results in this study suggest that AHLs or AHL-like molecules affect oral biofilm formation, encouraging the application of QQ strategies for oral health improvement, and reinforcing the importance of personalized approaches to oral biofilm control.

## Introduction

Most oral bacteria are embedded in a matrix of extracellular polymers of host and bacterial origin and attached to the tooth surface forming a biofilm, presenting altered phenotypes compared to planktonic cells (Donlan and Costerton, 2002; Marsh, 2006). The two major oral bacterial diseases, dental caries and periodontal disease (PD), are caused by changes in local environmental conditions that result in a loss of the ecological equilibrium in the oral microbiota, eventually leading to a pathogenic biofilm (Marsh, 1994; Marsh, 2003; Belda-Ferre et al., 2012; Hajishengallis et al., 2012). Therefore, it is not a single pathogen but rather the entire community residing in the oral cavity and its functional activities that are responsible for the progression of these diseases (Duran-Pinedo and Frias-Lopez, 2015; Yost et al., 2015). In this context, interfering with biofilm formation processes through modulation of microbial interactions has been proposed as a relevant strategy for preventing and treating oral polymicrobial diseases (Simón-Soro and Mira, 2015).

Cell density-dependent gene-regulation processes, known as quorum sensing (QS), have been proposed as potential targets for developing strategies to inhibit oral biofilm formation (Muras et al., 2018b; Muras and Otero, 2020; Muras et al., 2020b). Among the three most studied QS signals, the presence of Gram-positive autoinducer peptides has been reported in various oral streptococci, while the universal autoinducer-2 (AI-2) has been found in both Gram-positive and Gram-negative oral pathogens (Whittaker et al., 1996; Frias et al., 2001; Burgess et al., 2002; Kolenbrander et al., 2006). The *N*-acyl-homoserine lactones (AHLs), the QS signals typically used by Gram-negative bacteria, have traditionally been considered non-relevant in the oral cavity due to the lack of success in detecting their production in pure cultures of oral pathogens in the past (Whittaker et al., 1996; Frias et al., 2001; Burgess et al., 2002; Jakubovics and Kolenbrander, 2010; Huang et al., 2011; Guo et al., 2014). Despite this, a diversity of AHLs has been found in saliva samples and extracted teeth, including C_8_-HSL, C_14_-HSL, and C_18_-HSL (Kumari et al., 2006; Muras et al., 2020b). Additionally, AHL-producing bacterial strains have been isolated from dentine caries (Goh et al., 2014; Goh et al., 2016) and human tongue surface (Yin et al., 2012a; Yin et al., 2012b; Chen et al., 2013). Previous studies had observed that AHLs and AHL analogs modified protein expression in *Porphyromonas gingivalis* (Komiya-Ito et al., 2006; Asahi et al., 2010) and the production of OC_8_-HSL has been described in pure cultures of this periodontopathogen (Muras et al., 2020b). Moreover, a recent study on the effect of exogenous AHLs on *in vitro* oral biofilms proved that certain AHLs affected bacterial lactic acid production and protease activity and that the addition of C_6_-HSL shifted the microbial composition of biofilms towards a periodontal bacterial profile (Muras et al., 2020a). In addition, bacterial gene clusters predicted to be related to homoserine lactone biosynthesis have been described in the genomes of bacteria from caries and periodontitis patients (Aleti et al., 2019). Together, these results suggest that the established role of AHLs in the current paradigm of cell-to-cell communication in oral biofilms should be revised (Muras and Otero, 2020; Muras et al., 2020b, Muras et al., 2022). Confirmation of the role of AHLs as QS signals in oral biofilm formation would open a broad range of possibilities for the prevention and treatment of oral diseases.

Different strategies to interfere with QS systems are present in natural environments. The use of these strategies in both natural and *in vitro* conditions has been successful in decreasing biofilm mass in single-species (Sun et al., 2021) and multispecies biofilms (Jo et al., 2016; Huang et al., 2019; Schwab et al., 2019; Jeong et al., 2020; Muras et al., 2020b; Muras et al., 2022). The use of these strategies is less likely to promote antimicrobial resistance than antibiotics, as they do not interfere directly with bacterial growth (Romero et al., 2014; Mayer et al., 2015; Romero et al., 2015, Mayer et al., 2018; García-Contreras et al., 2016; Guendouze et al., 2017; Muras et al., 2018b). The enzymatic inactivation of QS signals, also known as quorum quenching (QQ), has been thoroughly studied (Romero et al., 2015), especially enzymes capable of disrupting AHLs (Dong et al., 2001). Remarkably, the presence of QQ activity against AHLs has already been described in cultivable bacteria from saliva and dental plaque samples (Muras et al., 2020b). The use of Aii20J, a broad-range, highly active AHL-lactonase belonging to the metallo-beta-lactamase family (Mayer et al., 2015), has yielded interesting results for controlling several AHL-regulated phenotypes in Gram-negative pathogenic bacteria (Mayer et al., 2015, Mayer et al., 2018; Mayer et al., 2020) and reducing oral biofilm grown *in vitro* from saliva and in a mixed biofilm of six oral pathogens (Muras et al., 2020b).

In this study, we assessed the effect of Aii20J on oral polymicrobial biofilms generated *in vitro* using samples from healthy donors and patients with PD. Subgingival samples were chosen to inoculate the biofilms, as they are expected to be more representative of the periodontal microbiota than saliva samples. *In vitro* biofilms were characterized in terms of total biomass and microbial diversity achieved in the presence of the enzyme. Additionally, supragingival shotgun metagenomes were investigated for the presence of QS- and QQ-related sequences.

## Materials and methods

### Subject recruitment

An initial group of 17 patients (female/male: 11/6; mean age: 48 ± 13 years) and 35 additional patients (female/male: 19/16; 60 ± 15 years) were recruited from the University Dental Clinic of the Faculty of Medicine and Dentistry at the University of Santiago de Compostela between December 2019 and March 2020, and between June 2020 and May 2022, respectively. In the first group of patients (*n* = 17), saliva and subgingival samples were taken to optimize biofilm generation conditions (see section 2.5). In the second group of patients (*n* = 35), only subgingival samples were taken, as a result of the optimization done with the first group. Oral health status was assessed according to the proceedings from the 2017 World Workshop on the Classification of Periodontal and Peri-Implant Diseases and Conditions (Papapanou et al., 2018; Tonetti et al., 2018) (**Supplementary Tables S1, S2**). Antibiotic treatment up to one month prior to sampling was the exclusion criterium set for the study.

### Ethics statement

The investigation protocol 2009/319 for patient recruitment and sample handling, modified in July 2017, was approved by the Ethical Committee of Clinical Investigations of Galicia (Xunta de Galicia). Written informed consent was obtained from all participants in this study.

### Production and purification of the AHL-lactonase Aii20J

The QQ enzyme Aii20J, a lactonase enzyme obtained from the QQ marine bacterium *Tenacibaculum* 20J (Romero et al., 2011; Romero et al., 2014), was obtained as previously described (Mayer et al., 2018). Briefly, protein expression was induced by adding 1 mM Isopropyl-D-thiogalatopyranoside to a suspension of a recombinant *E. coli* BL21(DE3)pLysS containing Aii20J. The induced cells were lysed by sonication, and purification of Aii20J was achieved using the His GraviTrap affinity column protein purification kit (GE Healthcare). The remaining imidazole from the purification buffer solutions was removed by dialyzing the sample in D-tubes (MWCO 10 kDa) (Merck KGaA, Darmstadt, DE) in sterile Milli-Q water. When appropriate, purified Aii20J was added to the biofilms at a working concentration of 20 µg mL^-1^, corresponding to ten times the minimum concentration of purified enzyme needed to degrade 10 µM of C_6_-HSL in 24 h. The AHL-degradation capacity of purified Aii20J and remaining Aii20J activity in biofilms’ supernatants was routinely verified using a chromogenic assay (see “Quorum quenching activity solid plate assays” section below).

### Quorum quenching activity solid plate assays

The AHL degradation activity of Aii20J, both purified and after total incubation time in biofilm supernatants, was tested using solid plate assays. QQ activity in saliva samples was assessed using the same methodology. Assays were carried out using the AHL biosensor strains *Chromobacterium subtsugae* CV026 for short-chain AHLs (McClean et al., 1997; Harrison and Soby, 2020) and *C. violaceum* VIR07 for long-chain AHLs (Morohoshi et al., 2008). Samples were mixed with either C_6_-HSL (10 µM) or C_12_-HSL (10 µM) (Sigma-Aldrich, Merck). These AHLs are commonly used as representatives of short- and long-chain AHLs, respectively (Muras et al., 2020b). PBS pH 6.5 with the corresponding AHL (10 μM) was used as negative control (Muras et al., 2018b). The pH of the saliva samples was checked beforehand and acidified when it exceeded pH 7 to avoid the spontaneous opening of the lactone ring of the AHLs at high pH values (Yates et al., 2002). All mixtures were incubated for 24 h at 22°C.

The presence of the AHLs was detected by adding a mixture of an overnight culture of the corresponding biosensor and soft Luria-Bertani (LB) (0.8% agar) in a 1:4 proportion on top of an LB agar plate. Wells were manufactured onto the agar, and 100 µL of the sample were placed in each well. Plates were incubated for 24 h at 30°C. The lack of violet pigmentation, as opposed to the violet halo observed around the negative controls, indicated QS inhibition. This assay allows identifying in a rather precise manner if the QS inhibition is due to an enzymatic degradation or to the presence of a QS inhibitor molecule. Enzymatic degradation is often displayed as a lack of pigmented halo or as a halo of reduced diameter respect to the negative control. On the other hand, QS inhibitors diffuse with the AHL added to the sample; the different titers of both components, with the AHL normally present in a much higher titer, result in an inner non-pigmented halo surrounded by an outer violet halo (Muras et al., 2018a). Furthermore, this assay allows differentiating between QS inhibition (observation of growth-turbid halos) and cell growth inhibition (observation of transparent halos) (Muras et al., 2018a).

### Sample collection, biofilm generation, and biofilm quantification

The initial biofilm collection was derived from saliva and subgingival samples from seven healthy donors (codes H1 – H7) and ten patients with PD (codes P1 – P10) (**Supplementary Table S1**) and cultured immediately. Saliva samples were collected in sterile tubes and diluted 1:100 in each culture media used (Fernandez y Mostajo et al., 2017; Muras et al., 2020a). Subgingival samples consisted of gingival crevicular fluid (GCF) collected by inserting 16 sterile paper points (4 in each mouth quadrant) in the gingival sulcus for 1 minute. In patients with PD, samples were taken from the deepest periodontal pockets. Samples were transported in 1 mL of thioglycollate broth (Merck), from which a 1:53 dilution with the corresponding medium was made (Mira et al., 2019). For this initial collection, saliva and subgingival samples were inoculated in 1) Brain Heart Infusion (BHI) (PanReac, Barcelona, ES) in aerobic conditions and 2) Schaedler medium supplemented with vitamin K1 (0.1 mg L^-1^) (SCH-K1) (Conda Pronadisa, Madrid, ES) in anaerobic conditions. The Aii20J enzyme was added at the moment of the inoculation, at a final concentration of 20 µg mL^-1^. Negative controls received sterile Milli-Q water. The purified Aii20J (35 kDa in size) was filtered through a Centricon with a molecular weight cut-off of 10 kDa (Merck) to remove the enzyme and use the remaining soluble fraction as an additional control (**Supplementary Figure S7**). A heat-inactivated Aii20J (90 min at 121°C) that did not conserve AHL-degrading activity was also tested (**Supplementary Figure S7**). Biofilms were grown for 24 h at 37°C.

*In vitro* oral biofilms (*n* = 17) were generated using the culture media and conditions described above and measured in parallel using two different methodologies. The first is a modification of the Amsterdam Active Attachment model (AAA model) (Exterkate et al., 2010; Muras et al., 2020a). Briefly, the lids of 12-well cell culture plates (VWR) were replaced by custom-made stainless-steel lids onto which 12 silicone tubes were attached, allowing for the insertion of glass coverslips (18 × 18 mm) (Menzel Gläser, Braunschweig, DE) to serve as substrata for biofilm formation. The glass coverslips fit vertically into the culture plate wells, filled with 3 mL of the corresponding adjusted inocula (Muras et al., 2020b), prompting the active attachment of bacterial cells onto their surface. Aii20J was added at a final concentration of 20 µg mL^-1^. The AAA model allows the refreshment of culture media and treatments at 12 h by transferring the lid with the glass coverslips to a new plate. When required, anaerobiosis was achieved using an anaerobiosis jar (OXOID, Basingstoke, GB) with AnaeroGen 2.5L Atmosphere Generation System (OXOID). Biofilms were quantified by crystal violet (CV) assay. After incubation, coverslips were allowed to dry in a fresh plate, and biofilms were stained for 20 minutes with 2 mL of a 0.04% CV solution (v/v in distilled water) (Panreac). The excess dye was removed by washing twice with distilled water. Before releasing the CV for quantification, pictures were taken to perform visual examinations of the biofilms. When two independent observers found control and Aii20J-treated biofilms to be visually distinguishable, the biofilms were considered to have macroscopical differences (**Supplementary Figure S2**). Finally, CV bound to the biofilms was diluted in 3 mL of a 33% acetic acid solution (v/v in distilled water) (Scharlab, Barcelona, ES). Sample absorbance was measured at 590 nm. All experiments were performed in triplicate.

The second methodology used was the xCELLigence Real Time Cell Analyzer (RTCA) System (ACEA, Biosciences Inc., San Diego, US) (Muras et al., 2018b, Muras et al. 2020b; Mira et al., 2019). E-plates 16 (ACEA, Biosciences Inc.) were inoculated with 100 μL of the corresponding culture medium and 80 μL of the saliva/subgingival samples. To lactonase-treated samples, 20 μL of Aii20J (to a final concentration of 20 µg mL^-1^) were added, while 20 μL of sterile Milli-Q water were added to the control wells. An overlay of sterile paraffin was added when an anaerobic atmosphere was required. The xCELLigence measures changes in impedance as cells attach to the bottom of the E-plates, and the software computes cell index (CI) values that correspond to the amount of biofilm formed. All experiments were performed in triplicate.

A broader screening of culture conditions was then performed using subgingival samples (*n* = 35). Samples were collected following the previously described methodology and cultured in the AAA model. Culture conditions for four initial patients (H8, G1, P11, and P12) (**Supplementary Table S2**) were BHI in aerobiosis, BHI in anaerobiosis, SCH-K1 in anaerobiosis, and semi-defined McBain medium (McBain et al., 2005; Janus et al., 2015) in anaerobiosis. Subsequent biofilms from ten healthy donors (H9 – H18), two patients with gingivitis (G2, G3), and 19 patients with periodontitis (P13 – P31) (**Supplementary Table S2**) were inoculated in BHI-anaerobiosis and McBain-anaerobiosis. Biofilms were grown for 24 h at 37°C, with culture media and treatment refreshment at 12 h. Biofilm quantification was performed as previously described.

### Confocal-laser scanning microscopy

Subgingival-derived biofilms from patients P30 and P31 grown in McBain-anaerobiosis in the AAA model were further visualized using confocal-laser scanning microscopy (CLSM) to compare control and Aii20J-treated biofilms. Biofilms formed onto the glass coverslips were stained with two nucleic acid dyes; SYTO 9, which is membrane permeable and enters all bacterial cells, and propidium iodide (PI), which only enters membrane-damaged cells (LIVE/DEAD BacLight Bacterial Viability Kit, Thermo Fisher Scientific, Waltham, US). The staining solution was prepared by mixing SYTO 9 and PI in a 1:1 proportion and then diluting it 150-fold in sterile PBS (pH 7.4). Approximately 50 μL of the staining solution were poured onto each biofilm. Samples were kept for 15 minutes at room temperature and protected from light before image acquisition with a Leica Stellaris 8 FALCON confocal microscope, with the objective HC PL APO CS2 20x/0.75 Dry (Leica Microsystems GmbH, Wetzlar, DE). Biofilms were observed using the 499 nm laser excitation and 504 – 555 nm emission band for SYTO 9, and 561 nm excitation laser and 570 – 675 nm emission band for PI. Photographs were acquired at 20x magnification. To calculate the area covered by biofilms, eight fields for each condition were randomly selected and processed using the Leica Application Suite X software (LAS X v.3.7.4, Leica Microsystems). The area emitting photons from the SYTO 9 and PI channels was divided by the total field of view. The results are expressed in percentage of the area covered by biofilm structures in each condition.

### Harvesting of biofilms and DNA extraction

Genomic DNA for sequencing analysis was obtained from biofilms grown in the AAA model. Biofilms were harvested by transferring the glass coverslips into 5 mL of sterile PBS. Then, they were sonicated for 15 minutes to disperse the biomass. Genomic DNA extraction was done using the “DNeasy PowerBiofilm Kit” (Qiagen, Germantown, US), following the manufacturer’s instructions. Briefly, biofilms were subjected to chemical and mechanical lysis. Proteins and inhibitors were removed, followed by pH-driven precipitation of large insoluble macromolecules. Total genomic DNA was captured using a silica spin filter column and finally eluted. DNA concentration was measured using a NanoDrop (Thermo Scientific).

### Library preparation and microbiome analysis

Microbial genomic DNA (5 ng µL^-1^ in 10 mM Tris pH 8.5) was used to amplify the 16S rRNA gene V3-V4 hypervariable regions. After size verification, the libraries were prepared according to the supplier’s protocol (Illumina, Inc.), and the resulting 11 pM 20% on PhiX pooled library was sequenced using a 2x300 base pairs paired-end run (MiSeq Reagent kit V3(MS-102-3001)) on a MiSeq Sequencer.

Quality assessment was performed using the prinseq-lite program (Schmieder and Edwards, 2011). The pipeline DADA2 (Callahan et al., 2016) was used to analyze and cluster the sequences into amplicon sequence variants, classified to the genus level with the SILVA database (Quast et al., 2013). Computations and statistics were carried out in R RStatistics (R Core Team, 2022) using knitr, knitcitations, markdown (Allaire et al., 2014; Boettiger, 2014; Xie, 2014), biostrings, and vegan (Oksanen et al., 2017). The differential relative abundance of the bacterial genera identified was assessed with the packages Phyloseq and DESeq2 (Love et al., 2014). Differences were filtered with a log2 fold change threshold higher than 2 and by their base mean value, discarding the lower quartile. Beta diversity was studied using a PCoA of weighted UniFrac distances using the package Phyloseq. Differences between samples, grouped according to the studied variables, were analyzed using the PERMANOVA test implemented in the adonis function of the package vegan (Oksanen et al., 2017). Alpha diversity was analyzed with the R package Phyloseq (McMurdie and Holmes, 2013), using the Chao1 and the Shannon and Simpson indexes as richness and diversity estimators. The data’s normality and homoscedasticity were assessed before choosing parametric or nonparametric tests included in the package stat (R Core Team, 2022).

### Bacterial quantification by quantitative PCR

The amount of total bacteria, *P. gingivalis*, and *P. endodontalis* present in 33 subgingival biofilms from patients with periodontitis and grown in McBain-anaerobiosis in the AAA model was evaluated by quantitative PCR (qPCR) in a LightCycler 480 II Thermocycler (Roche Diagnostics GmbH, Mannheim, DE). The reactions were performed in a 20 μL volume containing LightCycler 480 II Probes Master (Roche Diagnostics GmbH), specific primers and probes for each bacteria or universal primers for Eubacteria (**Supplementary Table S3**), 5 μL of isolated DNA and PCR grade sterile water. Positive and negative controls and sample reactions were performed in duplicate. The samples underwent an initial amplification cycle of 95°C – 10 min, followed by 40 cycles of 95°C – 10 s, annealing at 60°C – 30 s and extension at 72°C – 1 s. Crossing points (C_P_) were calculated with LightCycler 480 Software 1.5 (Roche Diagnostics GmbH) using the second derivative maximum method. Crossing point values were then extrapolated to colony-forming units (CFUs) using standard curves, as described in Àlvarez et al. (2013). Briefly, to obtain standard curves, first pure bacterial cultures were generated in three biological replicates. When cultures reached log phase, CFU plating and, in parallel, DNA extractions for qPCR amplification were performed, both in 10-fold serial dilutions. Then, curves correlating CFUs/mL and C_P_ values were generated using the qPCR software. Reproducibility of the R^2^ values of the curves obtained from the different biological replicates was used as a quality criterium to consider the correlations reliable (Àlvarez et al., 2013).

### AHL extraction and preparation for HPLC-MS analysis

To detect the presence of AHLs at low concentrations in untreated biofilms, the supernatants of the biofilm cultures were extracted to concentrate the polar molecules and analyze them by high-performance liquid chromatography-mass spectrometry (HPLC-MS). Glass coverslips from the AAA models were transferred, together with the spent culture media, into fresh tubes (VWR) and sonicated for 15 minutes to disperse the biomass. Samples were then acidified with HCl 1 M to induce the circularization of the lactone rings (Yates et al., 2002). Samples were extracted sequentially with two volumes of ethyl acetate (Scharlab) and two volumes of dichloromethane (Scharlab) (Muras et al., 2020a). The organic phases were evaporated in a Rotavapor-R (Büchi), and the dry samples were resuspended in 1 mL of acetonitrile (Merck) and stored at -20°C until its quantification by HPLC-MS. Fresh culture media were also extracted following the same procedures.

HPLC-MS analyses were performed at the Unit of Chromatographic Techniques and Water Analysis (University of A Coruña, ES) using an HPLC 1100 series (Agilent). The mass spectrometer (MS) used was an API 4000 triple quadrupole (Applied Biosystems). A calibration curve was obtained running standard synthetic AHLs (with and without oxo- and hydroxy-substitutions, ranging from 4 to 18 carbons in their acyl chains). The calibration curve was used to quantify AHLs in the samples. Elution times and molecular spectra of the standard synthetic AHLs were used to identify the AHLs present in the samples (Romero et al., 2010; Mayer et al., 2018).

### Search of quorum-related sequences in oral metagenomes

A selection of QS and QQ-related sequences was made by manually searching NCBI’s databases (https://www.ncbi.nlm.nih.gov/) for annotated nucleotide and amino acid sequences with demonstrated activity. Selected QS sequences comprised AHL synthases (*n* = 36), AHL receptors (*n* = 28), the AI-2 synthase LuxS (*n* = 11), and AI-2 receptors (*n* = 3). PQS synthases (*n* = 2) and a PQS receptor (*n* = 1), were included as well. QS “dialect” systems were also selected, including non-AHL synthases (*n* = 5) and receptors (*n* = 10) in the analyses. Selected QQ sequences included three types of enzymes: lactonases (*n* = 63), acylases (*n* = 20), and paraoxonases (*n* = 3). Nucleotide sequences were translated into amino acid sequences using the software Expasy (Duvaud et al., 2021).

The initial 182 sequences were clustered into protein families using CD-HIT (Fu et al., 2012), establishing a minimum of 70% length coverage and 40% sequence identity to reduce redundancy. Even though several protein sequences have the same annotation, some were clustered into different protein families, reflecting evolutionary divergence, e.g., LuxS sequences were clustered into four groups with the representative type sequences corresponding to *Vibrio harveyi, Streptococcus pyogenes, Bacillus thuringiensis*, and *Bacteroides heparinolyticus*. The 182 sequences were clustered and grouped by function, resulting in 78 protein families. The accession numbers of the sequences in each protein family are available in **Supplementary Table S4**.

The representative sequences selected for each protein family were then used to query 118 shotgun oral metagenomes from the Human Microbiome Project (HMP) (Human Microbiome Project Consortium, 2012a; Human Microbiome Project Consortium, 2012b) (https://portal.hmpdacc.org/) (**Supplementary Table S5**) and seven oral metagenomes from a Spanish collection (Belda-Ferre et al., 2012). These 125 metagenomes corresponded to supragingival plaque samples. Due to the absence of metagenomic data from subgingival sites in the HMP, a “synthetic” subgingival metagenome was generated (**Supplementary Table S6**). To select the information contained in this metagenome, the 16S rRNA gene sequencing data from subgingival and supragingival sites available in the HMP (Human Microbiome Project Consortium, 2012b) was investigated. First, a comparison of the relative abundances of the bacterial genera present in subgingival and supragingival niches was done to evaluate the resemblance between both sites (**Supplementary Table S7**). Then, the bacterial genera of subgingival sites with abundance values over 1% were selected, and the genomes of the most representative species from each genera were retrieved (**Supplementary Table S6**) to generate the synthetic subgingival metagenome.

Sequence searches were performed using DIAMOND (Buchfink et al., 2021) with a minimum subject coverage of 70% and an e-value cut-off of 1e-10. Alignment results were parsed into tables using in-home scripts (available at https://github.com/genomica-fciencias-unam/tabla_genes). Statistical analyses were done using R (R Core Team, 2022) with the phyloseq (McMurdie and Holmes, 2013), ggplot2 (Wickham, 2009), vegan (Oksanen et al., 2017), and tydiverse (Wickham et al., 2019) packages. All R procedures are available as a jupyter notebook in the **Supplementary Material**.

### Statistical analyses

Statistical analyses were performed using Prism 8.3.0 (GraphPad, San Diego, CA, USA, www.graphpad.com) or R (R Core Team, 2022). Two-tailed Student’s *t-*tests (referred to in the text as *t-*tests) or ANOVAs were performed for normally distributed samples. Mann-Whitney tests were performed for non-normally distributed samples to compare control and Aii20J-treated biofilm mass and cell index values within the same donor. Wilcoxon matched-pairs signed-rank tests (referred to in the text as Wilcoxon tests) were performed to assess the significance of the differences between biofilms that were reduced in the presence of the enzyme and those that were increased in the presence of the enzyme, subject by subject, grouped according to their oral health status. Quantitative PCR results were analyzed using Mann-Whitney tests for independent samples. For all the statistical analyses, significant differences were determined with an α = 0.05.

## Results

### Effect of sample origin and cultivation systems on *in vitro* oral biofilm response to the AHL-lactonase Aii20J

**Figure 1:**
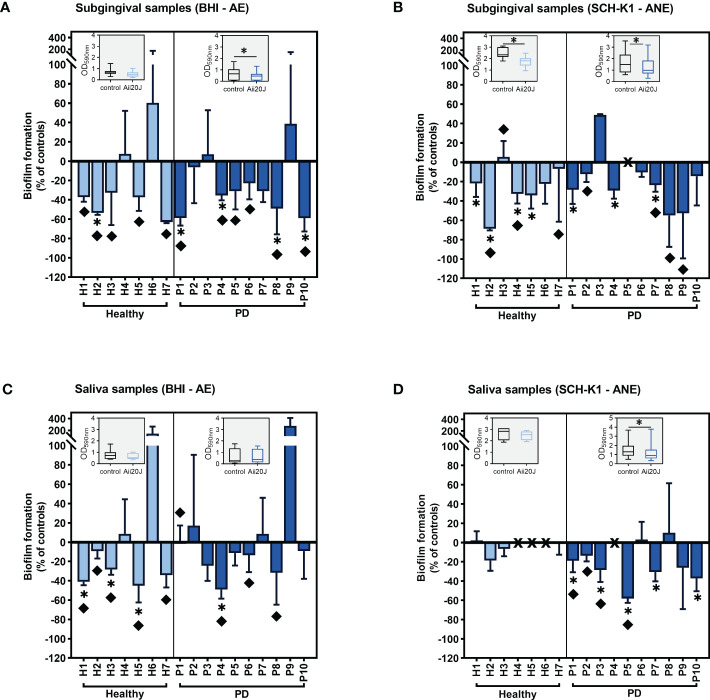
Inhibition of saliva and subgingival biofilm formation in the AAA model in the presence of Aii20J. Results are represented as mean ± SD (*n =* 3) of the percentage of biomass in Aii20J-treated biofilms compared to controls (OD_590nm_), in which control biofilms are represented by the value “0”. Negative values on the y-axis indicate that the Aii20J-treated biofilm was reduced respect to the control, whereas positive values indicate that the Aii20J-treated biofilm was increased respect to the control. Samples used to inoculate the biofilms were obtained from healthy donors (light blue bars) and patients with periodontal disease (PD; dark blue bars). All biofilms were grown for 24 h. Subgingival samples were grown in BHI and aerobiosis **(A)** and SCH-K1 and anaerobiosis **(B)**. Saliva samples were grown in BHI and aerobiosis **(C)** and SCH-K1 and anaerobiosis **(D)**. Asterisks (*) indicate statistical significance (*t-*tests, *α = 0.05). Diamonds (♦) represent macroscopical differences between untreated and Aii20J-treated biofilms. In samples P5 **(B)** and H4, H5, H6 and P4 **(D)**, marked with an “X” on the x-axis, biofilms could not be quantified. Inserted in each graph is a boxplot (median and interquartile range -IQR-, whiskers range from minimum to maximum) comparing absolute values of biofilm mass (OD_590nm_) in control and Aii20J-treated samples from healthy donors (*n =* 7) and PD patients (*n =* 10). Asterisks (*) indicate statistical significance of the differences between biofilms that were reduced and those that were increased in the presence of Aii20J (Wilcoxon tests, *α = 0.05). AE, aerobiosis. ANE, anaerobiosis. BHI, Brain Heart Infusion. SCH-K1, Schaedler medium supplemented with 0.1 mg L^-1^ of vitamin K1.

To assess the potential use of Aii20J in the prevention of oral diseases, the biofilm inhibitory capacity of the enzyme was first explored using two cultivation techniques: the AAA model (Exterkate et al., 2010; Muras et al., 2020a) and the xCELLigence system (ACEA, Biosciences Inc.). The AAA model was assembled with vertically placed glass coverslips onto which bacterial cells can only attach in an active manner. The AAA model allows the refreshment of culture media, further prompting biofilm formation. Besides, the substrata can be easily manipulated at the end of the incubation time to perform quantification assays, microscopy observations, and DNA extraction, allowing a complete characterization of the biofilms that is hampered by the use of the CV assay alone (Muras et al., 2018b). On the other hand, the xCELLigence system is a real-time monitoring technique based on cell index (CI) values that correlate to the amount of biofilm formed onto the bottom of modified culture plates (Mira et al., 2019). This system has been used to monitor oral biofilm formation (Mira et al., 2019), but is not suitable to detect structural differences in biofilms. Both systems were inoculated with saliva and subgingival samples from 17 subjects (**Supplementary Table S1**). Samples were grown aerobically in BHI medium and anaerobically in SCH-K1. Both culture media contain a high protein load, but the Schaedler medium has additional components that favor the growth of anaerobes, such as cysteine and hemin, and in addition to the commercial recipe, vitamin K1 (Murray, 1978; Marsh et al., 2016). The use of either aerobic or anaerobic atmospheres aimed to assess whether the effect of the enzyme would be different on bacterial communities grown under such conditions. We observed important variations in biofilm formation capacity among samples (**Supplementary Figures S3, S4**) without significant differences in biomass values between healthy and PD groups grown in the AAA model (*t-*test) (**Supplementary Figure S3**). In the xCELLigence system, PD samples yielded significantly higher cell index values than healthy samples for subgingival biofilms grown in BHI and saliva biofilms grown in SCH-K1 (*t-*test) (**Supplementary Figures S4A, D**). In addition, initial biofilm mass achieved in the control condition did not correlate with sensitivity to Aii20J in any cultivation system (Pearson r) (data not shown). Regarding the effect of Aii20J on biofilm formation, when cultivated in the AAA model, subgingival biofilms were more sensitive to the enzyme than saliva biofilms in both culture media (**Figure 1**). Analysis of the effect of the enzyme on subgingival biofilms grouped according to the oral health status of the patients showed a significant decrease in PD-derived biofilms grown in BHI and both healthy- and PD-derived biofilms grown in SCH-K1 (Wilcoxon test) (**Figures 1A, B**). For saliva biofilms cultured in the same conditions in the AAA model, only PD samples grown in SCH-K1 presented a significant biomass decrease (Wilcoxon test) (**Figures 1C, D**). However, the xCELLigence system was much less sensitive than the AAA model in detecting the effect of Aii20J, as significant changes were only found in two saliva-derived biofilms treated with Aii20J, either in BHI or SCH-K1 (*t-*test) (**Supplementary Figure S2**). Therefore, the AAA biofilm cultivation model and subgingival samples were selected for further experiments.

Additionally, we evaluated the presence of QS inhibitory activity among the initial saliva samples against short- (C_6_-HSL) and long-chain (C_12_-HSL) AHLs with standardized bioassays (Romero et al., 2011). QQ activity against C_12_-HSL was common, with five out of seven saliva samples from healthy donors and seven out of eight saliva samples of PD origin inhibiting AHL detection by the biosensor strain VIR07 (**Supplementary Figure S5**). Conversely, QQ against C_6_-HSL was scarce, with only two healthy and one PD saliva samples decreasing violacein production in the biosensor strain CV026 (**Supplementary Figure S5**). The small volume of the saliva samples collected did not allow assessing the presence of AHLs in these samples.

**Figure 2:**
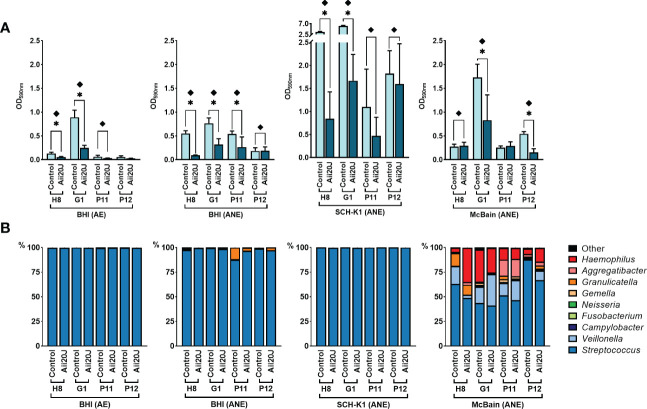
Effect of culture media and conditions on *in vitro* subgingival-derived biofilm formation **(A)** and bacterial diversity **(B)** in the presence of Aii20J. Subgingival samples from four patients were grown in the AAA model for 24 h in four different culture conditions: BHI in aerobiosis, and BHI, SCH-K1, and McBain medium in anaerobiosis. **(A)** Biofilm formation, represented as mean ± SD (*n =* 3) of control and Aii20J-treated biofilms absorbance values (OD_590nm_). Asterisks (*) indicate statistical significance (*t-*tests, *α = 0.05). Samples marked with a diamond (♦) presented macroscopical differences between untreated and Aii20J-treated biofilms. **(B)** Relative abundance (in percentage) of the most prevalent genera in the biofilms, as assessed by 16S rRNA gene sequencing. Genera detected in lower relative abundance appear under the category “Other”. AE, aerobiosis. ANE, anaerobiosis. BHI, Brain Heart Infusion. SCH-K1, Schaedler medium supplemented with 0.1 mg L^-1^ of vitamin K1.

To assess which culture media and incubation conditions resulted in higher microbial diversity within the PD-derived biofilms, we chose four culture conditions: BHI-aerobiosis, BHI-anaerobiosis, SCH-K1-anaerobiosis, and in addition, McBain-anaerobiosis (**Figures 2A, B**). The use of McBain culture medium provides with a high protein load, and like the SCH-K1 medium, McBain comprises cysteine, hemin, and vitamin K1, that favor the proliferation of Gram-negative anaerobes. To achieve a higher resemblance with the oral environment, the McBain medium contains also mucin, one of the major salivary polypeptides, present in the acquired enamel pellicle. Experiments were performed using subgingival samples, as they are expected to be more representative of the periodontal microbiota than saliva samples. Regarding microbial diversity, the genus *Streptococcus* was predominant in all biofilms (**Figure 2B**), but McBain medium harbored the most diverse biofilms, with increased proportions of genera such as *Veillonella, Granulicatella, Gemella*, and *Haemophilus* both in control and Aii20J-treated biofilms, as compared with the other culture media. The presence of the QQ enzyme resulted in significant reductions of subgingival-derived biofilm mass even in Gram-positive predominated biofilms obtained in BHI and SCH-K1 media, without significantly changing bacterial relative abundance at the genus level (**Figures 2A, B**).

### Effect of the AHL-lactonase Aii20J on *in vitro* subgingival-derived biofilm formation and bacterial diversity

Once we established the optimal conditions for obtaining diverse oral biofilms in the AAA model, we studied a greater number of samples, assessing changes in biofilm mass and microbial diversity in the presence of Aii20J. Subgingival samples were chosen as inocula since they present higher sensitivity to Aii20J than saliva samples, as seen in previous experiments (**Figure 1**). For these experiments, 35 patients were recruited; 11 healthy donors, three patients with gingivitis, and 21 with periodontitis (**Supplementary Table S2**). Biofilms were grown in BHI-anaerobiosis and McBain-anaerobiosis to compare the effect of Aii20J on biofilms dominated by Gram-positive bacteria and in more diverse biofilm communities, respectively. As seen with the initial set of patients, biofilm formation abilities varied widely depending on the donor (**Supplementary Figure S6**), with no significant differences in biomass values between healthy and PD groups being observed (*t-*test) (**Supplementary Figure S6**). Biomass values of untreated biofilms did not correlate with sensitivity to Aii20J (Pearson r) (data not shown).

**Figure 3:**
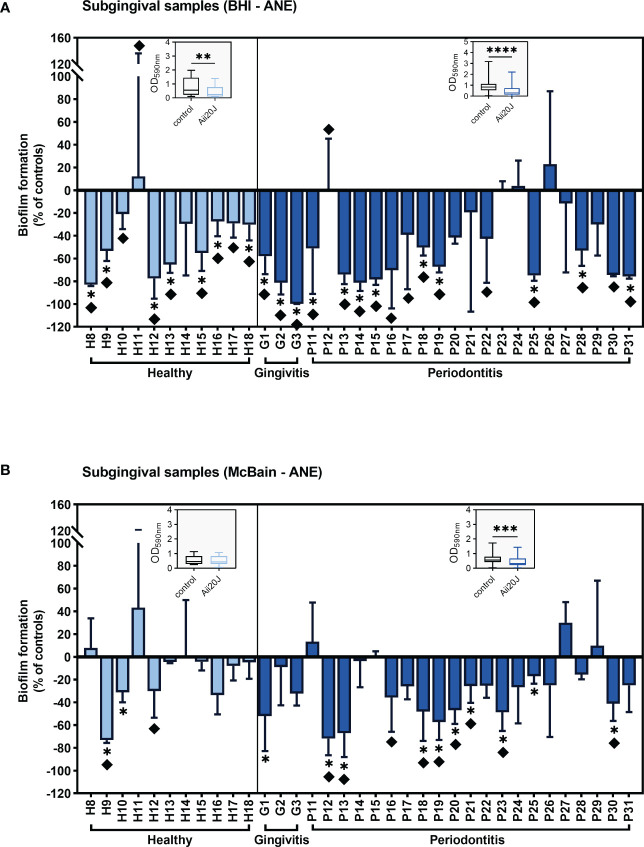
Inhibition of subgingival-derived biofilm formation in the presence of Aii20J. Results are represented as mean ± SD (*n =* 3) of the percentage of biomass in Aii20J-treated biofilms compared to controls (OD_590nm_), in which control biofilms are represented by the value “0”. Negative values on the y-axis indicate that the Aii20J-treated biofilm was reduced respect to the control, whereas positive values indicate that the Aii20J-treated biofilm was increased respect to the control. Samples used to inoculate the biofilms were obtained from healthy donors (light blue bars) and patients with gingivitis or periodontitis (dark blue bars). All biofilms were grown on the AAA model for 24 h. Subgingival samples were cultured in anaerobiosis in BHI **(A)** and McBain medium **(B)**. Asterisks (*) indicate statistical significance (*t-*tests, *α = 0.05). Diamonds (♦) represent macroscopical differences between untreated and Aii20J-treated biofilms. Inserted in each graph is a boxplot (median and interquartile range -IQR-, whiskers range from minimum to maximum) comparing absolute values of biofilm mass (OD_590nm_) in control and Aii20J-treated biofilms from healthy donors (*n =* 11) and patients with gingivitis and periodontitis (*n =* 24). Asterisks (*) indicate statistical significance of the differences between biofilms that were reduced and those that were increased in the presence of Aii20J (Wilcoxon tests, **α = 0.01, ***α = 0.001, ****α = 0.0001). ANE, anaerobiosis. BHI, Brain Heart Infusion.

Biomass was reduced in the presence of Aii20J in biofilms grown in both BHI-anaerobiosis and McBain-anaerobiosis, with few exceptions (**Figure 3**), confirming previous results obtained in *Streptococcus*-dominated biofilms (**Figure 1**). When the effect of Aii20J was analyzed by grouping the biofilms according to the oral health classification of the patients, PD-derived biofilms were significantly reduced in all culture conditions, whereas healthy-derived biofilms were significantly reduced only when grown in BHI (Wilcoxon test) (**Figure 3**). In subgingival, PD-derived biofilms, macroscopical differences (**Supplementary Figure S1**) were detected in 71% of biofilms grown in BHI and 38% of biofilms grown in McBain in the presence of Aii20J, and biomass decrease was statistically significant in 50% and 42% of the samples, respectively (*t-*test) (**Figure 3**). In subgingival, healthy-derived biofilms, macroscopical differences were found in 91% of biofilms grown in BHI and 18% of biofilms grown in McBain. Additionally, biomass reduction due to Aii20J was significant in 64% of healthy biofilms grown in BHI and 18% of healthy biofilms grown in McBain (*t-*test) (**Figure 3**). Assessment of additional controls, i.e., the fraction smaller than 10 kDa resulting from the purified Aii20J solution and the heat-inactivated Aii20J, yielded no differences in biofilm mass compared with water-only negative controls in biofilms from two periodontal donors susceptible to Aii20J (**Supplementary Figure S7**).

Subgingival biofilms from patients P30 and P31 grown in McBain were visualized under CLSM to compare biofilms that presented different responses to Aii20J in the CV assay (**Figure 3B**). Biofilms from patient P30 were significantly reduced in biomass in the CV assay and also showed a significant reduction in biofilm coverage when comparing CLSM photographs of control (11.0 ± 4.9%) and Aii20J-treated biofilms (5.8 ± 3.7%) (*t-*test) (**Supplementary Figure S8**). On the other hand, biofilms from patient P31 presented no significant differences in biofilm mass in the CV assay nor in biofilm coverage between control (3.26 ± 2.57%) and Aii20J-treated biofilms (3.57 ± 1.43%) (**Supplementary Figure S8**). However, control biofilms of patient P31 were predominantly stained with PI, whereas Aii20J-treated biofilms were predominantly stained with SYTO 9 (**Supplementary Figure S8**).

**Figure 4:**
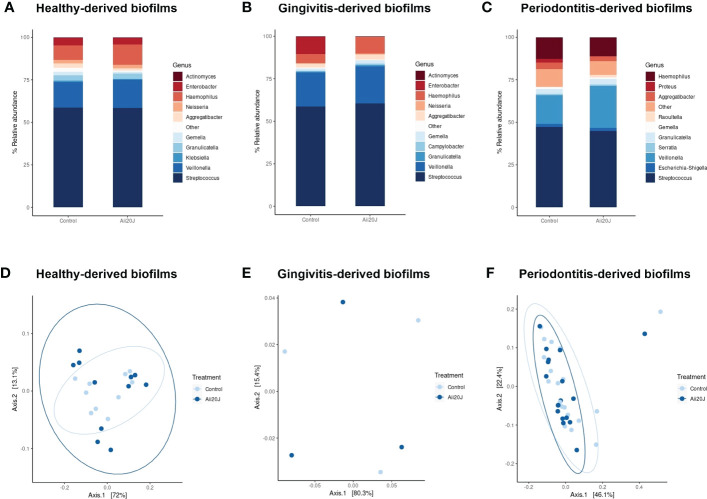
Relative abundance of the bacterial genera identified in the biofilms in the presence of Aii20J. Relative abundance is represented as the percentage of the ten most relatively abundant bacterial genera identified in each biofilm. Genera detected in lower relative abundance appear under the category “Other”. Subgingival samples were grown in the AAA model for 24 h in McBain, with and without Aii20J. Samples were obtained from healthy donors **(A)**, *n =* 11), patients with gingivitis **(B)**, *n =* 3), and patients with periodontitis **(C)**, *n =* 19). Principal Coordinate Analysis (PCoA) of weighted Unifrac plots of the microbiome structure of the samples obtained from healthy donors **(D)**, *n =* 11), patients with gingivitis **(E)**, *n =* 3), and patients with periodontitis **(F)**, *n =* 19) are shown.

**Figure 5:**
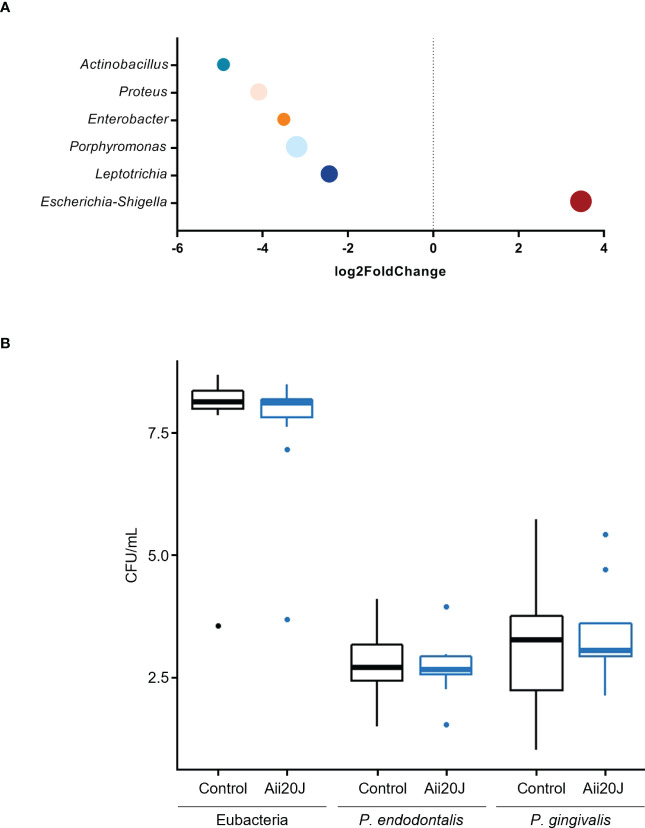
Differential microbial abundance of important genera identified in subgingival, periodontitis-derived biofilms (*n =* 19) in the presence of Aii20J. Samples were grown in the AAA model for 24 h in McBain, with and without Aii20J. **(A)** Genera with significant (α = 0.05) differential abundance in Aii20J-treated biofilms are shown. Data were analyzed using the DESeq2 package (Love et al., 2014). Genera with log2 fold change values < 2 or > 2 were considered to be significantly reduced or increased in Aii20J-treated biofilms, respectively. **(B)** Quantification, using qPCR, of total 16S rRNA, *P. gingivalis* and *P. endodontalis*, comparing control (black boxes) and Aii20J-treated biofilms (blue boxes). Boxplots represent the median and interquartile range (IQR), and whiskers range from Q1 - 1.5 IQR to Q3 + 1.5 IQR. Mann-Whitney tests (α = 0.05) for independent samples were performed. CFU, colony-forming unit.

In parallel to biofilm quantification, biofilm mass from control and Aii20J-treated samples grown in McBain was collected for DNA extraction, as we previously observed that this culture medium allowed higher microbial diversity in biofilms from subgingival samples (**Figure 2**). Sequencing of the 16S rRNA gene confirmed the dominance of the genus *Streptococcus*, reaching a 45 – 61% relative abundance in the biofilms (**Figures 4A–C**). *Veillonella, Haemophilus, Granulicatella, Gemella*, and *Aggregatibacter* were also found in all biofilms within the top ten most relatively abundant genera. Beta diversity examined with Principal Coordinate Analysis (PCoA) and alpha diversity indexes revealed no significant differences in microbial composition between control and Aii20J-treated biofilms (**Figures 4D–F**, **Supplementary Figure S9**). In addition to diversity indexes, a differential abundance analysis was performed grouping the biofilms according to the oral health status of the donors. In periodontitis-derived biofilms, the genus *Porphyromonas*, among others, significantly decreased its differential abundance in the presence of Aii20J (**Figure 5A**). Further quantification of *Porphyromonas gingivalis* and *Porphyromonas endodontalis*, both species linked to oral diseases (Hajishengallis et al., 2012; de Brito et al., 2020), was done using qPCR on periodontitis-derived biofilms (*n =* 19). No significant differences were found for *P. gingivalis* nor *P. endodontalis* CFUs between control and Aii20J-treated biofilms (Mann-Whitney test) (**Figure 5B**). Total 16S rRNA gene quantification for Eubacteria was performed as a normalization control.

Last, supernatants of untreated biofilms (*n =* 16) were investigated for the presence of the most common AHLs (from C_4_-HSL to C_18_-HSL, either with or without oxo and hydroxy substituents) by HPLC-MS analysis. None of these standard AHLs were identified in any of the samples.

### Abundance of quorum-related sequences in oral supragingival metagenomes

**Figure 6:**
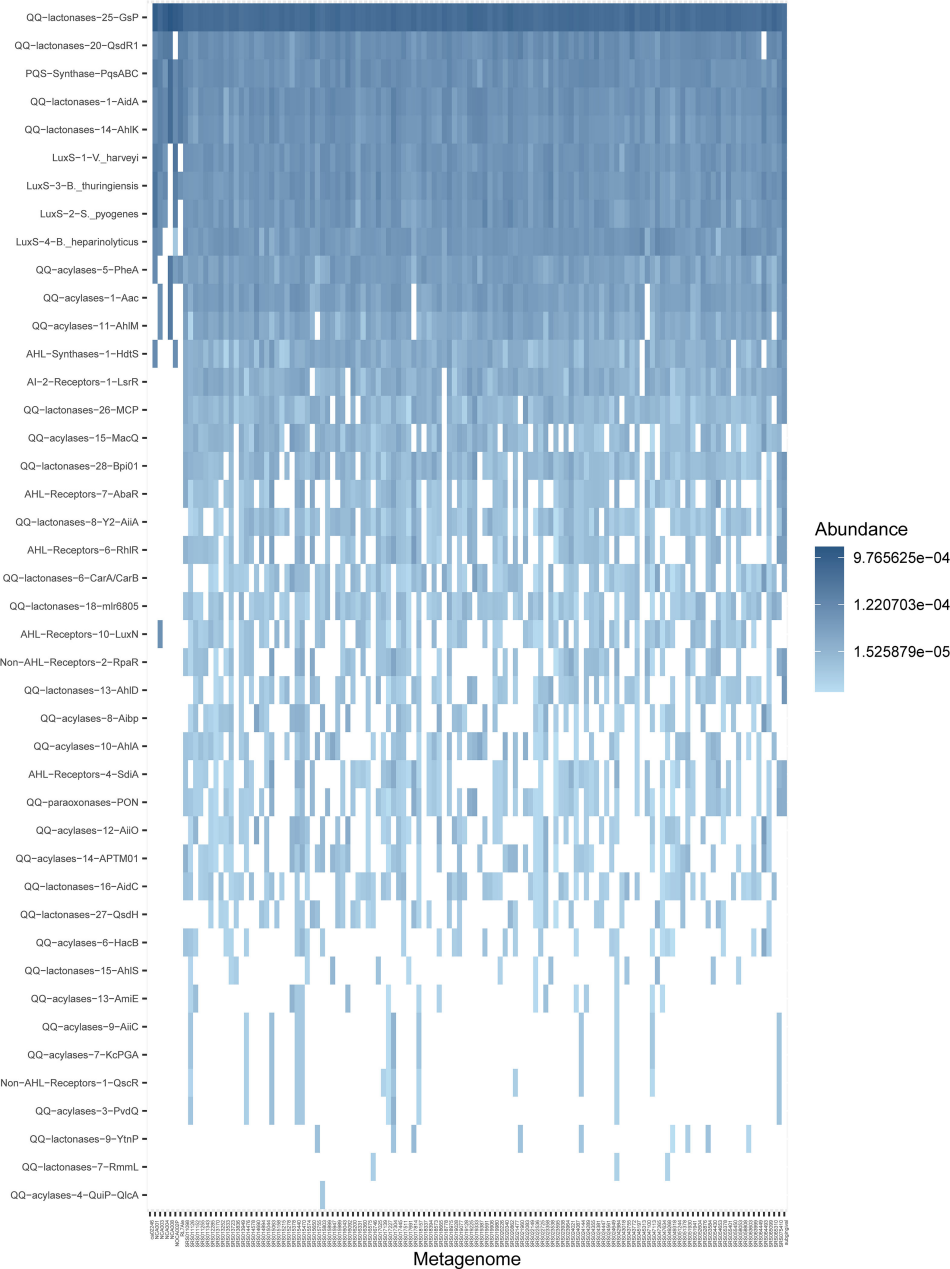
Abundance of selected quorum sensing and quorum quenching protein families in supragingival shotgun metagenomes. Results are represented as a heatmap where rows are sorted by abundance and columns by non-metric multidimensional scaling distances. The last column on the right is the synthetic subgingival metagenome generated for this study (**Supplementary Table S6**). The complete set of queried metagenomes is available in **Supplementary Table S5**. Non-detected proteins are represented by blank spaces. QS, quorum sensing; QQ, quorum quenching; AHL, *N*-acyl-homoserine lactone; AI-2, autoinducer-2.

The absence of standard AHLs in the supernatants of biofilm cultures and the impossibility of performing the same analytical procedures to detect AHLs on the original saliva and subgingival samples due to their reduced volume prompted the investigation of the presence of sequences related to AHL synthesis and sensing, as well as AHL enzymatic degradation in 125 metagenomes from supragingival plaque samples available in public databases (Belda-Ferre et al., 2012; Human Microbiome Project Consortium, 2012a; Human Microbiome Project Consortium, 2012b), and one synthetic subgingival metagenome that comprised the most representative bacterial species from the subgingival niche (**Supplementary Table S6**), and that displayed very similar results to those of the supragingival metagenomes in terms of sequences found and their abundance. The analysis of these metagenomes revealed a surprisingly high abundance of proteins homologous to the AHL synthase HdtS (**Figure 6**, **Supplementary Table S4**). This protein family comprised three HdtS sequences from *Pseudomonas*, and it appeared in nearly every metagenome, almost as abundantly as the four protein families of LuxS, included as reference in this analysis (**Figure 6**). Among the AHL receptors investigated, three protein families appeared in elevated abundance in both the supragingival metagenomes and the synthetic subgingival metagenome. Representative members of these families are AbaR of *Acinetobacter*, RhlR of *Pseudomonas*, and the promiscuous AHL-receptor SdiA of *Burkholderia* (**Figure 6**). These protein families appeared present in a high number of metagenomes, although their abundance was lower than that observed for HdtS. It is worth mentioning that the protein family represented by RhlR also comprised several SdiA sequences, including those from *Escherichia coli* and *Salmonella*, that were divergent from those classified together with the SdiA sequences of the *Burkholderia* family.

In addition to AHL synthases and receptors, we investigated the supragingival and subgingival metagenomes for the presence of AHL-inactivating enzymes: lactonases, acylases, and paraxonases. Protein families of lactonase and acylase enzymes appeared in extremely high abundances, in some cases, even higher than LuxS sequences (**Figure 6**). These *in silico* results align with those obtained *in vitro* on the high abundance of QS inhibitory activity among saliva samples (**Supplementary Figure S5**).

## Discussion

This work aimed to evaluate the potential of the QQ enzyme Aii20J to prevent biofilm formation of periodontal origin based on *in vitro* studies. Given the rise of antimicrobial resistance in dentistry, alternative approaches to control periodontal diseases need to be explored (Cieplik et al., 2014; Rams et al., 2014). Several studies have described the effectiveness of QQ strategies against monospecific (Sun et al., 2021) and polymicrobial biofilms (Jo et al., 2016; Huang et al., 2019; Schwab et al., 2019; Jeong et al., 2020). Specifically, the inhibitory effect of the AHL-lactonase Aii20J on biofilm formation obtained from saliva samples has already been described (Muras et al., 2020b). Aii20J is a wide-spectrum, extremely thermoresistant enzyme that has no genotoxicity (UNE-EN ISO 10993-3:2015) and no cytotoxicity in eukaryotic cells (OECD Guideline 471) (unpublished results). Besides, we have confirmed that Aii20J retains its activity after 24 months in a commercial mouthwash at room temperature (unpublished results). Despite being a member of the metallo-beta-lactamase super-family, Aii20J does not affect beta-lactam antibiotics (Mayer et al., 2015) nor interferes with bacterial growth (Muras et al., 2018b), reducing the risk of inducing antimicrobial resistance. These properties make Aii20J a promising candidate for developing antibiofilm strategies in the oral health context.

Our results indicate that the AAA model, together with CV staining, is a more sensitive methodology than the xCELLigence system for detecting different responses of biofilms to Aii20J (**Figure 1**). The AAA model allows quantification, macroscopic evaluation (**Supplementary Figure S1**), and microscopy imaging (**Supplementary Figure S8**) of the biofilms attached to the glass coverslips, complementing the CV assay for detecting structural differences (Muras et al., 2018b). The AAA model also facilitates the refreshment of treatments and culture media, making more nutrients available for the attached cells, eliminating loose bacteria and metabolic waste, and ensuring the assessment of actively attached cells only. On the other hand, the xCELLigence system monitors biofilm growth during early adhesion stages (Muras et al., 2018b; Mira et al., 2019), reaching saturation after 10 h of incubation (Muras et al., 2018b). In the AAA model, an end-point quantification technique, we describe significant biofilm alterations due to Aii20J. In contrast, in the xCELLigence system, a real-time monitoring technology, no significant changes were found for most samples. Thus, Aii20J may affect biofilm in its maturing phase rather than in the initial attachment stages.

Different culture media and conditions were examined to achieve biofilms that contained higher proportions of anaerobic Gram-negative bacteria. Biofilms inoculated in BHI and SCH-K1 harbored populations dominated by the genus *Streptococcus* (**Figures 2** and **4**). However, McBain medium, containing a higher protein load than the other culture media, allowed the proliferation of more diverse communities (**Figure 4**). These conditions (the use of McBain in anaerobiosis) have already been proposed as an adequate option to recreate the gingival crevice niche *in vitro* (Janus et al., 2015), as high availability of nutrients and low oxygen conditions have been described to favor the proliferation of asaccharolytic and proteolytic bacteria *in vivo* (Drucker, 2000).

The addition of Aii20J resulted in significant biofilm mass decreases in most conditions tested. In the initial set of biofilms grown in BHI-aerobiosis and SCH-K1-anaerobiosis from different inocula, up to 41% of subgingival-derived biofilms were significantly reduced compared with the 29% of significantly reduced saliva biofilms (**Figure 1**). This differential response of the biofilms to the enzyme could be explained by the variation of bacterial communities between saliva and subgingival samples (Simón-Soro et al., 2013). We also describe an increased sensitivity to Aii20J in PD samples compared to healthy samples, finding significant biofilm reductions in PD-derived biofilms treated with Aii20J in three of four conditions tested (**Figure 1**). Differences in the behavior of healthy- and PD-derived biofilms to the enzyme could be attributed to differences in genera that appear at low relative abundance. In subsequent experiments, subgingival samples from healthy and PD patients were grown under conditions driving to more diverse biofilms. Once more, Aii20J caused significant reductions in biomass in both diverse biofilms (McBain-anaerobiosis), and biofilms dominated by Gram-positive taxa (BHI-anaerobiosis), with 35 – 87% of samples presenting macroscopical differences, respectively (**Figure 3**). These results further confirm the effectiveness of this enzyme as an *in vitro* biofilm inhibitory strategy. Even in non-responsive biofilms, as for patient P31, we could observe an effect of the enzyme on the biofilms using CLSM (**Supplementary Figure S8**). Specifically, untreated biofilms from patient P31 were predominantly stained with PI, i.e., contained mostly membrane-damaged cells, whereas Aii20J-treated biofilms were predominantly stained with SYTO 9, indicating that most cells were membrane-intact (**Supplementary Figure S8**). Reductions in response to Aii20J ranged from 50 – 99% of initial biomass in several biofilms and between 20 – 50% of initial biomass in most biofilms, whereas a small proportion presented little or no reductions when treated with Aii20J. This contrasting behavior among biofilms generated from different patients has already been described with the antibiotic amoxicillin (Mira et al., 2019). The observed heterogeneity in biofilm formation abilities and response to the QQ enzyme indicates the importance of using samples from different patients for assessing any oral treatment, as it allows observing different responses among individuals when considering the administration of a treatment. This is of special importance in the current context of oral health, with a growing consensus towards personalized therapy and the proposals of studying individual responses on complex samples before treatment administration (Bartold, 2017; Belibasakis et al., 2019).

Further investigations on the microbial diversity of biofilms grown in McBain revealed no significant changes in microbial relative abundance between control and Aii20J-treated samples (**Figure 4**, **Supplementary Figure S9**). In fact, similar studies on the effect of lactonases on polymicrobial biofilm communities also reported that relative abundances in the microbial population were not changed in the presence of QQ agents (Jo et al., 2016; Schwab et al., 2019). Conversely, some works did observe changes in the relative abundance of oral- (Muras et al., 2020b) and water-associated communities (Huang et al., 2019) in the presence of lactonases. The study of Muras et al. (2020b), relied on the use of saliva samples from two subjects, whereas the present study increased to 33 the number of subjects studied, and used subgingival samples instead of saliva for investigating changes in microbial populations under the influence of Aii20J. These differences can explain the contrast in the findings reported by both studies. Additionally, Muras et al. (2020b) described changes in bacterial population only at the species level, as 99.74% of the population belonged to the genus *Streptococcus.* In contrast, in the present study, biofilm populations comprised *Streptococcus* proportions of 45 – 63%, and the assessment of changes in bacterial population was done at the genus level, which can also explain the differences in the results of both studies.

Surprisingly, and despite the clear inhibitory effect observed in the presence of the Aii20J lactonase, none of the most common AHLs were detected in the supernatants of untreated biofilm cultures in this study. However, AHLs such as C_8_-HSL have been detected *ex vivo* in extracted teeth, and C_8_-HSL, C_14_-HSL, and C_18_-HSL have been found *in vivo* in saliva samples (Muras et al., 2020b). In addition, several oral isolates have been demonstrated to produce AHLs in pure or controlled mixed cultures *in vitro* (Kumari et al., 2006; Yin et al., 2012a; Yin et al., 2012b; Chen et al., 2013; Goh et al., 2014; Goh et al., 2016; Muras et al., 2020b), and predicted AHL-related genes have been described in oral genomes (Aleti et al., 2019). Here, culture conditions could be affecting the production of AHLs within the *in vitro* biofilms, either as a consequence of the selection of non-AHL producer bacteria or as a result of culture conditions affecting AHL producers. The latter has been previously demonstrated in the human pathogen *Acinetobacter*, which switches on and off its AHL-producing systems depending on incubation conditions *in vitro* (Mayer et al., 2018). Moreover, the low volume of biofilm and culture media available for AHL extraction could have hampered their detection by HPLC-MS, and we cannot fully rule out the presence of AHLs acting at a micro-scale within the biofilms.

The analysis of 125 supragingival plaque metagenomes and an additional synthetic subgingival metagenome revealed a high abundance of proteins related to AHL biosynthetic pathways. The abundance of LuxS was used as a reference, as it is implicated in central metabolism and, therefore, widely represented in Gram-positive and Gram-negative bacteria. However, AI-2, the molecule resulting from the detoxification of S-adenosylmethionine by the action of LuxS, can also act as a QS signal in some species (Schauder et al., 2001; Winzer et al., 2002). The fact that the response regulator of AI-2, the LuxR protein of *V. harveyi* (Showalter et al., 1990), is present in lower abundance in the metagenomes investigated (protein family “AI−2−Receptors−1−LsrR”) (**Figure 6**), indicates a primary metabolic role of LuxS in these samples. In this study, we describe a remarkably high abundance of sequences related to HdtS synthases in both supragingival metagenomes and the synthetic subgingival metagenome (**Figure 6**). HdtS does not belong to the main AHL synthase families, and it has been demonstrated to produce several AHLs, including OHC_14:1_-HSL, an uncommon AHL that can act as a bacteriocin (Laue et al., 2000). Besides, the analysis of the supragingival metagenomes revealed three protein families of AHL receptors present in high abundance levels among the metagenomes (**Figure 6**). Two of these families comprised several sequences of SdiA. SdiA is described as a LuxR solo, i.e., a bacterial receptor that cannot produce AHLs but respond to them (Michael et al., 2001). SdiA has been demonstrated to be highly promiscuous, presenting a broad ligand-binding specificity and being able to respond to molecules other than AHLs (Nguyen et al., 2015; Styles and Blackwell, 2018). Hence, we cannot fully disregard the possibility of signal molecules different from AHLs being the cognate ligands of SdiA receptors and being present in the oral environment. The increasing volume of studies on LuxR solos has revealed that these receptors can bind a wide range of AHL derivatives and molecules containing aromatic groups derived from fatty acid metabolic pathways (Tobias et al., 2020). These observations subscribe to those of Aleti et al. (2019), who found putative AHL-biosynthesis genes in the genomes of human oral taxa. Altogether, these results suggest a role of uncommon AHLs or yet-undescribed AHL-like molecules in oral biofilms that are not identified when using standard AHLs as reference but that could be sensed by broad-range specificity receptors, such as SdiA and other LuxR solos. The findings reported herein with the use of supragingival metagenomes constitute novel knowledge on AHL-related metabolism in the oral cavity. A first approach to assess the abundance of these sequences in bacteria from the subgingival niche has been done with a synthetic subgingival metagenome. With the increase in the number of metagenomic studies and publicly available metagenomic data, further investigations will help confirm the abundance of QS-related sequences in the oral cavity, specifically in the subgingival niche.

Last, the search for sequences related to AHL-quenching also yielded surprising results, with a widespread abundance of QQ enzymes in the supragingival metagenomes and the subgingival synthetic metagenome investigated (**Figure 6**). Finding the protein family “QQ-lactonases-14-AhlK” among the most abundant groups is particularly interesting (**Figure 6**). This protein family contains, among others, the enzyme AiiB from *Agrobacterium tumefaciens*, a lactonase with a broad specificity of substrates (Liu et al., 2007). The existence of several QQ enzymes capable of degrading AHL derivatives and even molecules other than AHLs has been described in the literature (Hiblot et al., 2013; Bergonzi et al., 2019). The high abundance in supra- and subgingival metagenomes of QQ enzymes that may be degrading other molecules apart from AHLs aligns with the finding of promiscuous receptors in those same metagenomes (**Figure 6**), strengthening the idea of the presence of AHL-like molecules in the oral cavity. Therefore, we hypothesize that the Aii20J-mediated biofilm inhibitory effect described here may not be derived from the action of Aii20J on AHLs but on other signal molecules of related structure. A similar case has already been described with the QQ lactonase SsoPox, whose antibiofilm activity against *Pseudomonas aeruginosa* monospecies biofilm is more dependent on its promiscuous activity on the *P. aeruginosa* PQS signal than on its activity against AHLs (Rémy et al., 2020). When Rémy et al. (2020) compared SsoPox with a lactonase without activity against PQS, the latter presented a much lower antibiofilm activity despite being more active against AHLs, suggesting that wider-range lactonases have greater antibiofilm properties. Hence, our results on the antibiofilm activity of the lactonase Aii20J on oral samples could be explained by a possible susceptibility of uncommon AHLs or AHL-like molecules to the enzymatic activity of Aii20J. This is further reinforced by the finding of the PQS synthase (protein family “PQS-Synthase-PqsABC”), in high abundance in the supragingival and subgingival metagenomes (**Figure 6**). The confirmation of the presence of such molecules would imply a more complex signaling network in oral biofilms than currently established. Furthermore, the effect of Aii20J on biofilms dominated by *Streptococcus*, a Gram-positive that is neither expected to respond to AHLs nor is affected by this QQ enzyme in *in vitro* pure cultures (Muras et al., 2018b), could be partially explained by the introduction of non-AHL signals in the scenery. Future investigations on the substrate specificity of Aii20J towards molecules other than standard AHLs will help validate this hypothesis.

We describe the use of QQ strategies, specifically of the QQ lactonase Aii20J, as successful in reducing multispecies biofilms *in vitro.* We propose the use of Aii20J as a modulatory strategy for oral disease control, as it results in lower bacterial load within the biofilm. The biofilms generated in the presence of the enzyme are expected to respond better to other treatments, such as antibiotic therapy, due to their reduced structural density, and their potentially increased porosity, presenting QQ strategies as possible coadyuvants in these therapies. Despite these promising results, further *in vitro* studies, including the evaluation of the effect of Aii20J at a transcriptional and proteomic level are required to understand the mechanisms underneath the behavioral changes detected in such biofilms. Additionally, changes in enzymatic activities within these polymicrobial communities must be assessed, as functional activities such as proteases or siderophore production are intimately linked to bacterial virulence. Previous studies have already described changes in enzymatic activities driven by the presence of AHLs in *in vitro* oral biofilms (Muras et al., 2020a), suggesting that similar changes could be observed with the use of AHL-degrading enzymes. Regarding the abundance of QS-related sequences in oral metagenomes, since all available metagenomes were of supragingival origin, the investigation of subgingival samples for the presence of AHL-related sequences would shed light on the extent of their distribution in the oral cavity, confirming a correlation with the periodontal diseases and endorsing the use of strategies targeting QS components for oral health care. The final objective of testing QS-targeting strategies would be their transfer from *in vitro* experiments to *in vivo* and clinical trials. To do so, it would be interesting to assess the possible synergies of Aii20J with antimicrobials. Also, it would be necessary to assess the optimal *in vivo* delivery doses, retaining time, and other pharmacological aspects when considering the addition of the QQ enzyme to oral products.

Findings in this work suggest an important role of AHLs or AHL-like molecules in oral biofilm formation. Targeting these molecules results in significant biomass reductions *in vitro* with no significant alterations in microbial relative abundance. This study is first in describing a remarkably high abundance of an AHL synthase and promiscuous AHL receptors on supragingival and subgingival metagenomes. Overall, these results indicate that QQ strategies could be useful for controlling oral biofilm formation while highlighting the importance of a personalized assessment in the clinical practice.

## Data availability statement

The datasets presented in this study can be found in online repositories. The names of the repository/repositories and accession number(s) can be found in the article/**Supplementary Material**.

## Ethics statement

The studies involving human participants were reviewed and approved by Ethical Committee of Clinical Investigations of Galicia, Xunta de Galicia (Investigation protocol 2009/319, modified in July 2017). The patients/participants provided their written informed consent to participate in this study.

## Author contributions

All authors contributed extensively to the work presented in this paper. AP and AMu performed experiments, analyzed data, and wrote the manuscript with contributions from AA, AS-O, GA, and LDA. PO-C recruited the patients, collected the samples, and provided each patient’s oral health status classification. AMi helped in experiment planification and formal analysis. VB gave technical support and conceptual advice. AO conceived the project, designed experiments with contributions from VB, corrected the manuscript, and supervised the study. All authors discussed the results and implications and commented on the manuscript at all stages. All authors contributed to the article and approved the submitted version.
