# Supplementary material for: The quorum quenching enzyme Aii20J modifies in vitro periodontal biofilm formation
Source: Front Cell Infect Microbiol. 2023 Feb 2;13:1118630. doi: 10.3389/fcimb.2023.1118630 (PMC9932050; doi:10.3389/fcimb.2023.1118630)
Supplement: Supplementary file 1 [file DataSheet_1.pdf]

1 Supplementary Material [1]

1.1 Supplementary Figures

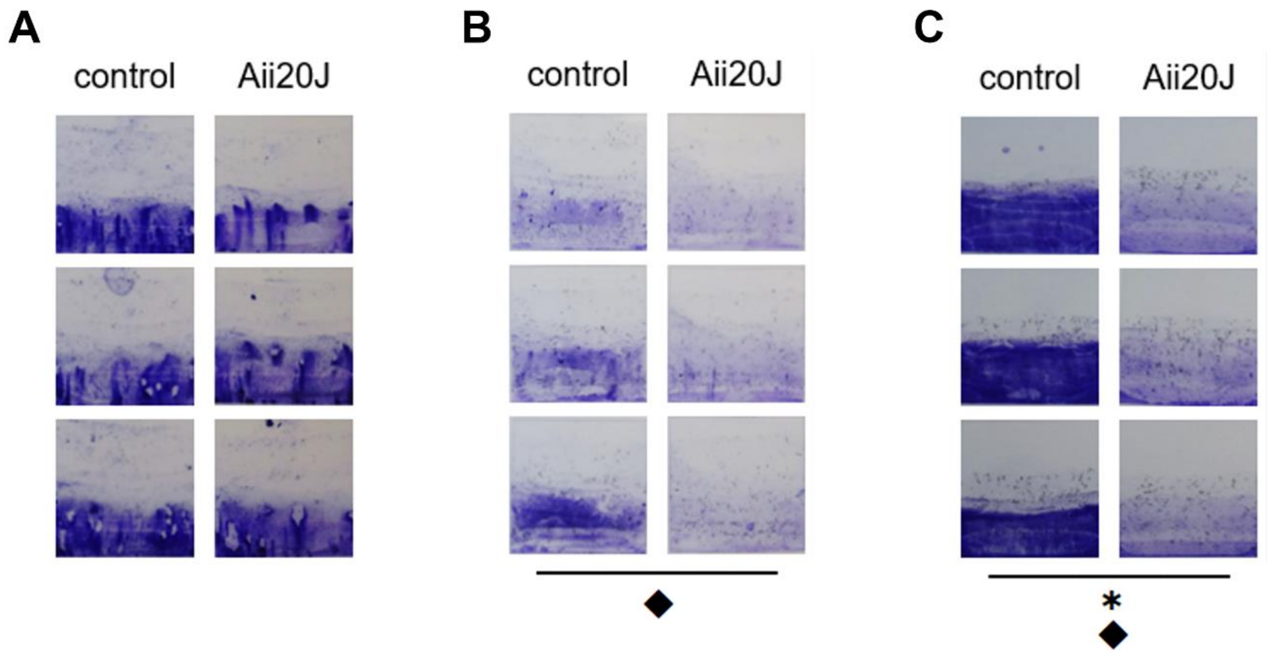

Supplementary Figure S1. Visual examination of biofilms grown in the AAA model and stained with CV. A. Control and Aii20J-treated biofilms that do not differ statistically nor visually. **B.** Control biofilms that are not significantly different in absorbance (OD_{590nm}) but differ visually (marked with a diamond; ♦) from Aii20J-treated biofilms. **C.** Control biofilms that differ statistically (*) and visually (♦) from Aii20J-treated biofilms.

[1] References for the Supplementary Material can be found at the end of this document.

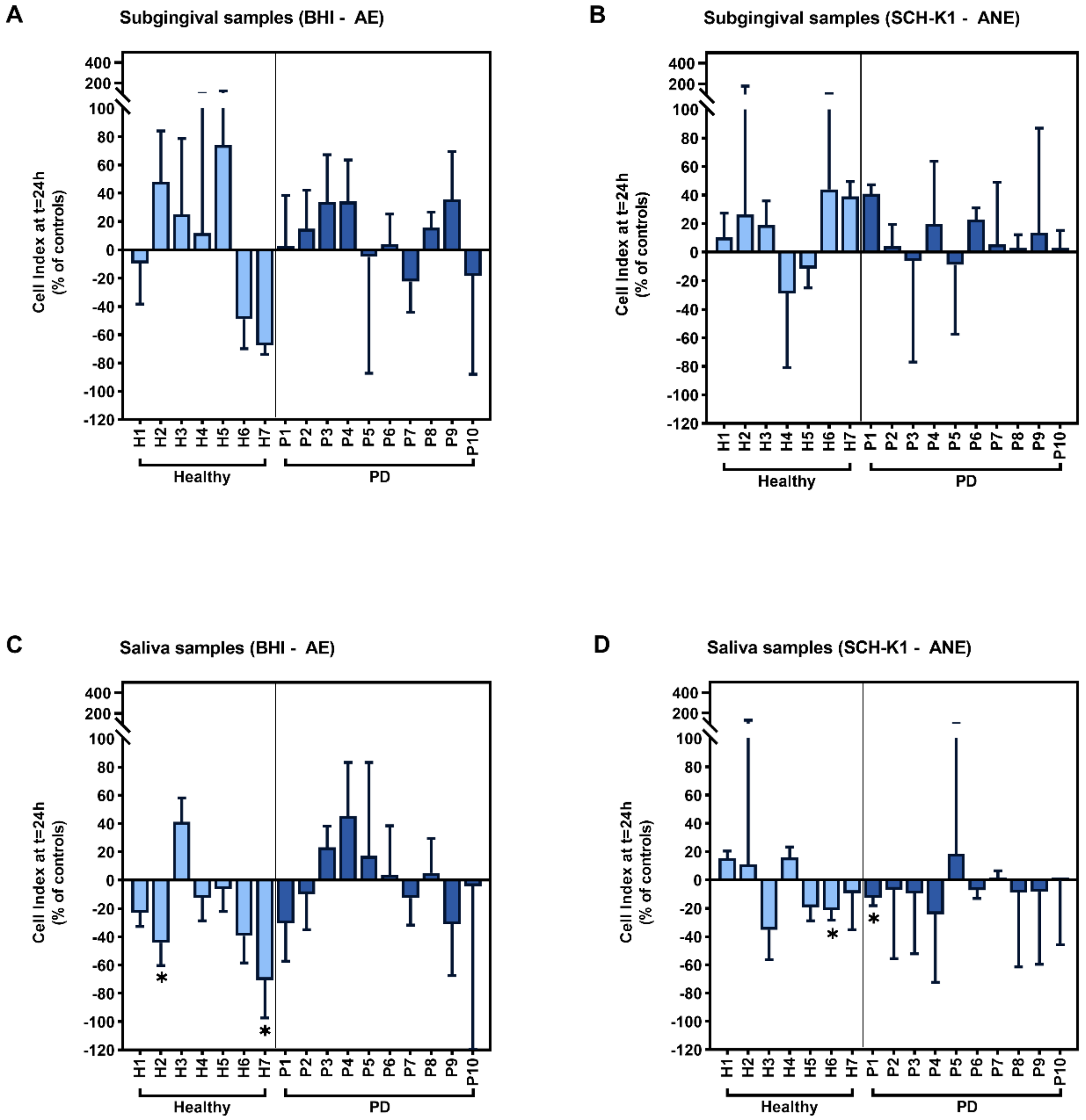

Supplementary Figure S2. Inhibition of biofilm formation in the xCELLigence system in the presence of Aii20J. Results are represented as mean ± SD (*n* = 3) of the percentage of cell index values of Aii20J-treated biofilms compared to their control, at t = 24h. Samples used to inoculate the biofilms were obtained from healthy donors (light blue bars) and patients with periodontal disease (PD, dark blue bars). Subgingival samples were grown in BHI and aerobiosis (**A**) and SCH-K1 and anaerobiosis (**B**). Saliva samples were grown in BHI and aerobiosis (**C**) and SCH-K1 and anaerobiosis (**D**). Asterisks (*) indicate statistical significance (*t*-tests, *α = 0.05). AE: aerobiosis. ANE: anaerobiosis.

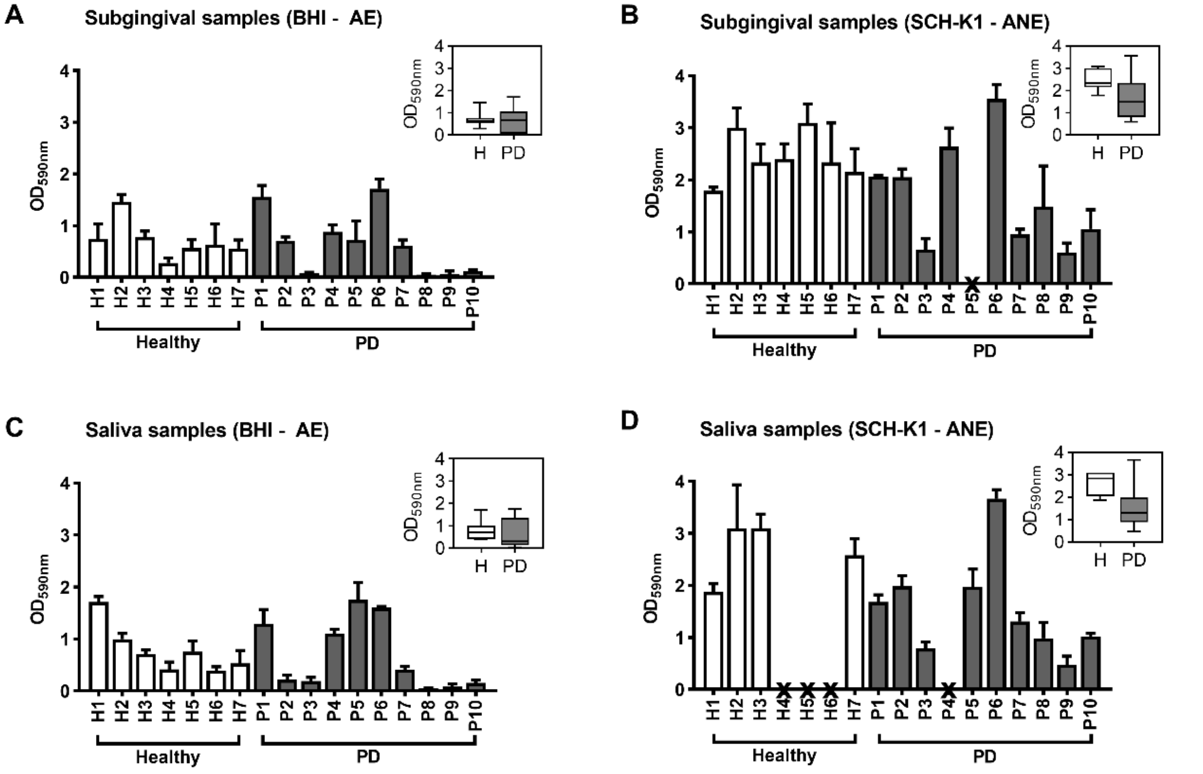

Supplementary Figure S3. Biofilm formation in the AAA model, represented as mean ± SD (*n* = 3) of control biofilms (OD_{590nm}). Samples used to inoculate the biofilms were obtained from healthy donors (white bars) and patients with periodontal disease (PD, grey bars). All biofilms were grown for 24h. Subgingival samples were grown in BHI and aerobiosis (**A**) and SCH-K1 and anaerobiosis (**B**). Saliva samples were grown in BHI and aerobiosis (**C**) and SCH-K1 and anaerobiosis (**D**). In samples P5 (**B**) and H4, H5, H6 and P4 (**D**), marked with an "X" on the x-axis, biofilm could not be quantified. Inserted in each graph is a boxplot (median and interquartile range -IQR-, whiskers range from minimum to maximum) comparing absolute values of control biofilms (OD_{590nm}) from healthy donors (n = 7) and patients with PD (n = 10). Asterisks (*) indicate statistical significance (*t*-tests; *α = 0.05). AE: aerobiosis. ANE: anaerobiosis.

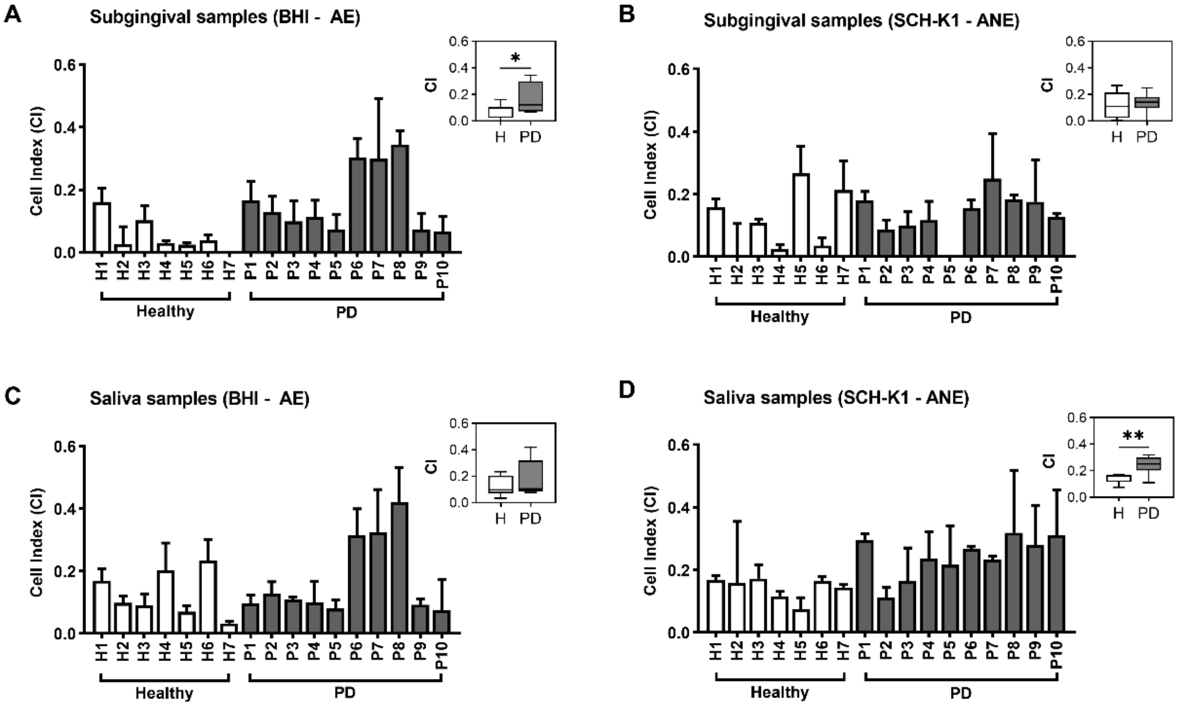

Supplementary Figure S4. Biofilm formation in the xCELLigence system at t = 24h. Results are represented as mean ± SD (*n* = 3) cell index (CI) values of control biofilms. Samples used to inoculate the biofilms were obtained from healthy donors (white bars) and patients with periodontal disease (PD, grey bars). Subgingival samples were grown in BHI and aerobiosis (**A**) and SCH-K1 and anaerobiosis (**B**). Saliva samples were grown in BHI and aerobiosis (**C**) and SCH-K1 and anaerobiosis (**D**). Inserted in each graph is a boxplot (median and interquartile range -IQR-, whiskers range from minimum to maximum) comparing absolute values of control biofilms (CI) from healthy donors (*n* = 7) and patients with PD (*n* = 10). Asterisks (*) indicate statistical significance (*t*-tests; *α = 0.05, **α = 0.01). AE: aerobiosis. ANE: anaerobiosis.

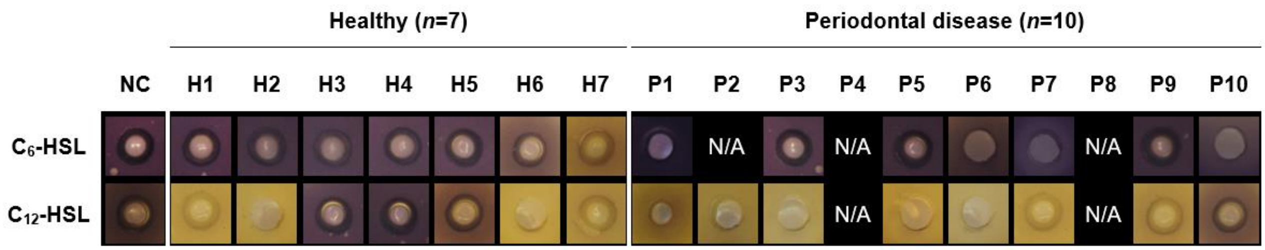

Supplementary Figure S5. QQ activity in saliva samples. A violacein halo around the wells after 24h of incubation with C_6-HSL or C_{12}-HSL indicates that the AHL preserves its original structure. Reduced or inexistent halos show partial and total degradation of the AHL, respectively. NC: negative control. N/A: not available.

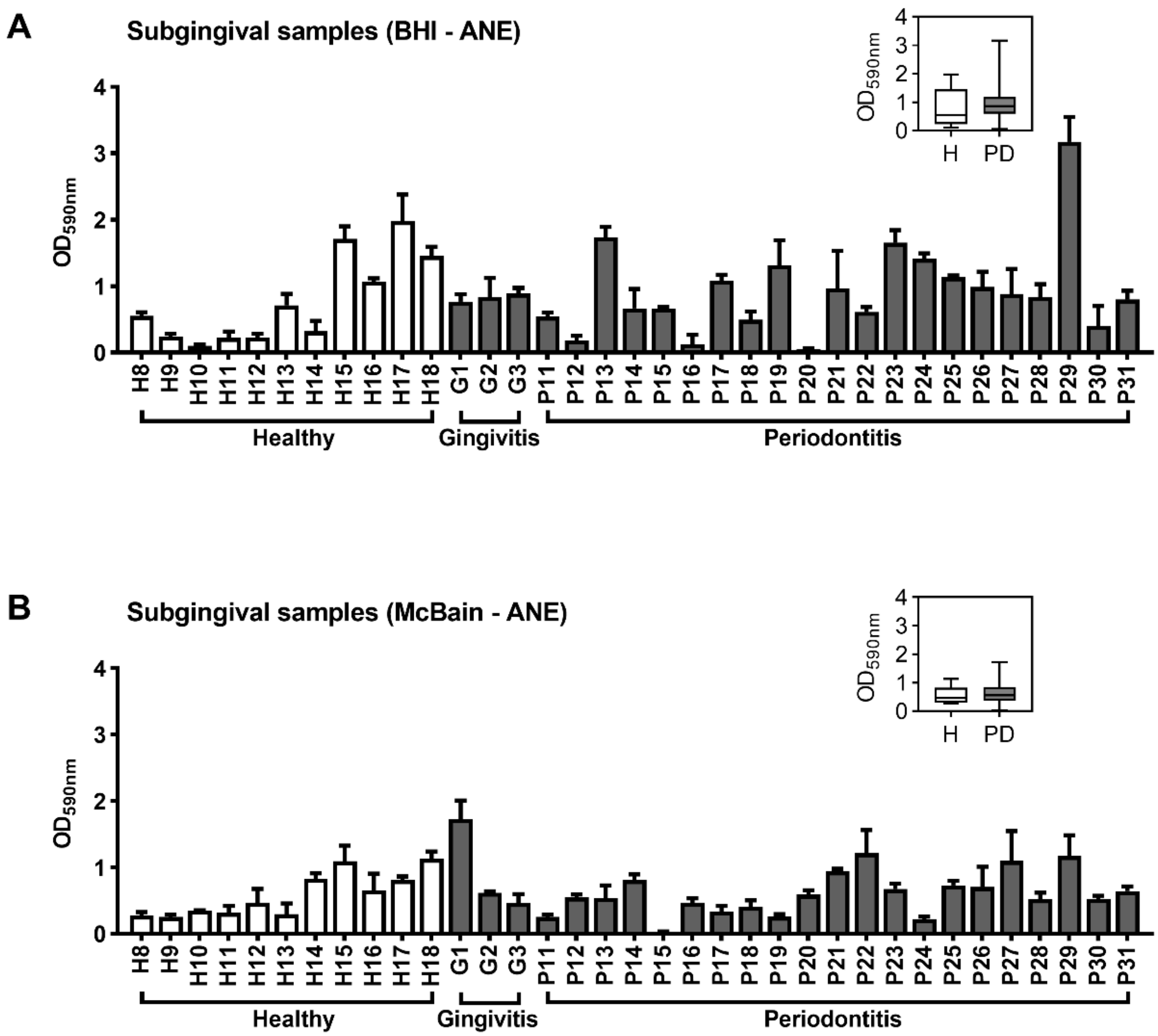

Supplementary Figure S6. Biofilm formation of subgingival samples in the AAA model, represented as mean ± SD (*n* = 3) of control biofilms (OD_{590nm}). Samples used to inoculate the biofilms were obtained from healthy donors (white bars) and patients with gingivitis and periodontitis (grey bars). All biofilms were grown for 24h. Biofilms were cultured in anaerobic conditions in BHI (**A**) and McBain medium (**B**). Inserted in each graph is a boxplot (median and interquartile range -IQR-, whiskers range from minimum to maximum) comparing absolute values of control biofilms (OD_{590nm}) from healthy donors (n = 11) and patients with gingivitis and periodontitis (n = 24). Asterisks (*) indicate statistical significance (t-tests; *α = 0.05). ANE: anaerobiosis.

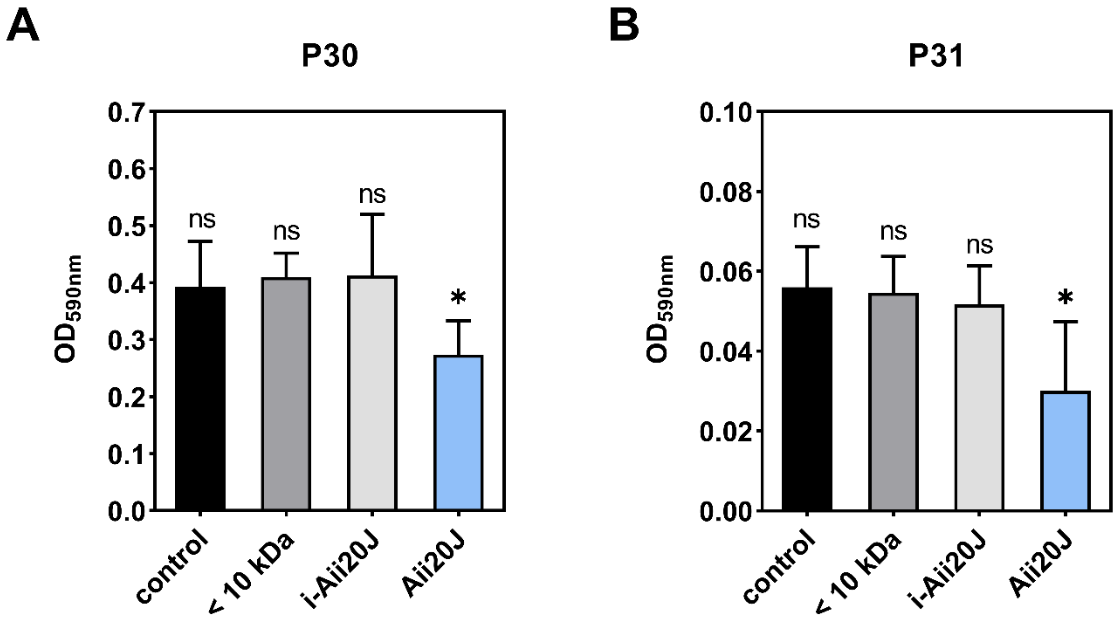

Supplementary Figure S7. Effect of the QQ enzyme ("Aii20J"), the soluble fraction of the purification product comprising all compounds under 10 kDa ("< 10 kDa"), and the heat-inactivated QQ enzyme ("i-Aii20J") on *in vitro* subgingival biofilm formation in comparison with the untreated biofilms ("control"). Biofilm mass was measured with CV staining (OD_{590nm}). Subgingival samples were grown in McBain and anaerobiosis in the AAA model for 24h. Samples used to inoculate the biofilms were obtained from two patients with periodontal disease: P30 (**A**), and P31 (**B**), from previous experiments. Results displayed in this figure were generated from independent experiments than the ones included in Figure 3. Results are represented as mean ± SD (n = 3) of biofilms. Asterisks (*) indicate statistical significance (ANOVA, *α = 0.05).

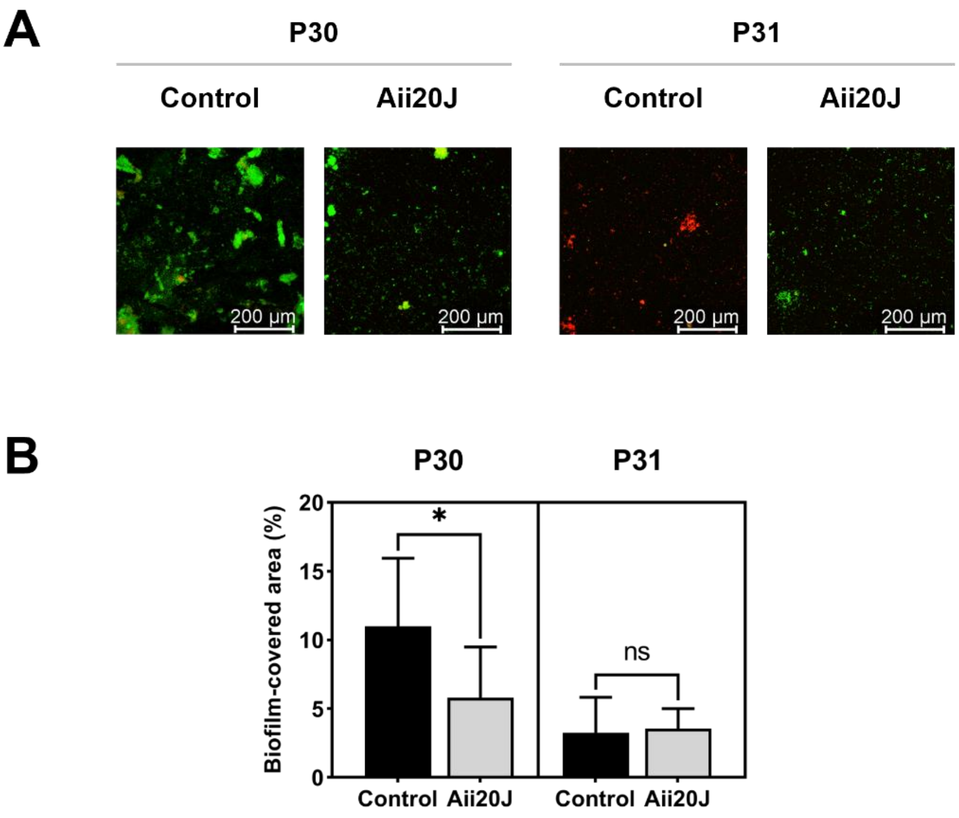

Supplementary Figure S8. Confocal-laser scanning microscopy visualization of subgingival biofilms grown in the AAA model in McBain and anaerobiosis, in control and Aii20J-treated conditions. Samples were taken from two patients with periodontitis (P30 and P31). **A.** Representative confocal photomicrographs of the areas with highest biofilm coverage, for each patient and condition, among eight randomly analyzed fields. The photomicrographs display FoV observed at 20x magnification. Scale bars represent 200 µm. Viable cells are stained with SYTO 9 and appear green, and membrane-damaged cells are stained with PI and appear red. **B.** Area covered by the biofilms, expressed as the percentage of biofilm-covered area relative to the total FoV (*n* = 8 fields at 20x). Asterisks (*) indicate statistical significance of the differences between Aii20J-treated biofilms (grey bars) and control biofilms (black bars) (*t*-tests, *α = 0.05).

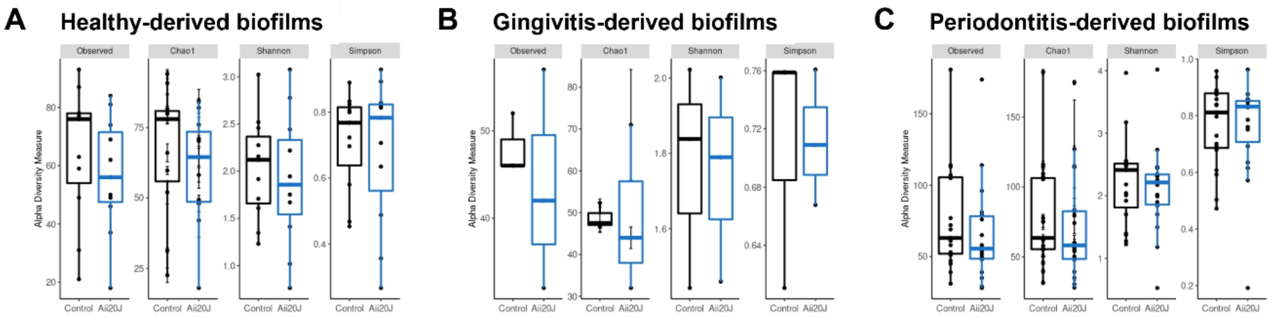

Supplementary Figure S9. Alpha diversity indexes of subgingival biofilms grown in the AAA model. Boxplots represent the median and interquartile range (IQR), and whiskers range from Q1 - 1.5 IQR to Q3 + 1.5 IQR. Biofilms were grown for 24h in McBain and anaerobic conditions. Samples used to inoculate the biofilms were obtained from healthy donors (**A**, *n* = 11), patients with gingivitis (**B**, *n* = 3), and patients with periodontitis (**C**, *n* = 19).

1.2 Supplementary Tables

Supplementary Table S1. **Classification of the oral health status of the first group of patients recruited in this study (*n* = 17).** Oral health status was assessed according to the proceedings from the 2017 World Workshop on the Classification of Periodontal and Peri-Implant Diseases and Conditions (Papapanou et al., 2018; Tonetti et al., 2018). F: female. M: male. RP: reduced periodontium.

	Code	Age	Sex	Classification
**Healthy (*n* = 7)**	H1	30	F	Healthy
	H2	57	M	Healthy, RP
	H3	46	F	Healthy
	H4	22	F	Healthy
	H5	39	F	Healthy
	H6	49	F	Healthy, RP
	H7	45	F	Healthy, RP
**Periodontal disease (*n* = 10)**	P1	48	F	Periodontitis (Stage II)
	P2	49	M	Periodontitis (Stage II)
	P3	47	F	Gingivitis
	P4	43	F	Gingivitis
	P5	44	F	Gingivitis
	P6	70	M	Periodontitis (Stage IV)
	P7	75	M	Periodontitis (Stage IV)
	P8	55	F	Periodontitis (Stage II)
	P9	65	M	Periodontitis (Stage III)
	P10	39	M	Periodontitis (Stage III)

Supplementary Table S2. Classification of the oral health status of the second group of patients recruited in this study (*n* = 35). Oral health status was assessed according to the proceedings from the 2017 World Workshop on the Classification of Periodontal and Peri-Implant Diseases and Conditions (Papapanou et al., 2018; Tonetti et al., 2018). F: female. M: male. RP: reduced periodontium.

	Code	Age	Sex	Classification
**Healthy (*n* = 11)**	H8	24	F	Healthy
	H9	49	M	Healthy, RP
	H10	50	F	Healthy
	H11	42	F	Healthy, RP
	H12	54	M	Healthy, RP
	H13	69	F	Healthy, RP
	H14	54	M	Healthy, RP
	H15	74	F	Healthy, RP
	H16	48	F	Healthy, RP
	H17	39	F	Healthy
	H18	63	F	Healthy, RP
**Periodontal disease (*n* = 24)**	G1	31	F	Gingivitis
	G2	77	F	Gingivitis
	G3	72	M	Gingivitis
	P11	64	F	Periodontitis (Stage III)
	P12	78	F	Periodontitis (Stage II)
	P13	62	M	Periodontitis (Stage III)
	P14	73	M	Periodontitis (Stage II)
	P15	38	F	Periodontitis (Stage I)
	P16	64	M	Periodontitis (Stage II)
	P17	57	F	Periodontitis (Stage IV)
	P18	54	M	Periodontitis (Stage III)
	P19	70	M	Periodontitis (Stage II)
	P20	74	M	Periodontitis (Stage II)
	P21	64	F	Periodontitis (Stage II)
	P22	55	F	Periodontitis (Stage I)
	P23	65	F	Periodontitis (Stage III)
	P24	64	M	Periodontitis (Stage III)
	P25	51	F	Periodontitis (Stage III)
	P26	85	M	Periodontitis (Stage III)
	P27	70	M	Periodontitis (Stage III)
	P28	71	M	Periodontitis (Stage II)
	P29	86	M	Periodontitis (Stage III)
	P30	51	F	Periodontitis (Stage II)
	P31	59	M	Periodontitis (Stage II)

Supplementary Table S3. Sequences of the sets of primers (F: forward; R: reverse) and probes (P) used for the quantification of each bacterial species. The last columns indicate the normalized concentrations and the amplicon size.

**Species**	**Target gene**	**Sequence**	**Concn (µM)**	**Length (bp)**
P. endodontalis	*rpoB*	F: TGTTAAGGATGGGAAAGTCGAT	0.8	147
		R: GCGTAGCGTGACTTTACTTTGTC	0.8	
		P: #6[a]	0.2	
P. gingivalis[b]	*rgpB*	F: GCCTTACCGTCTTTCACGAT	0.5	206
		R: AAGATGCTCGACACATGGAC	0.5	
		P: CCATCGTCAGAGAGCGTAGC[c]	0.2	
Eubacteria[d]	16S rRNA	F: TCCTACGGGAGGCAGCAGT	0.6	462
		R: GGACTACCAGGGTATCTAATCCTGTT	0.7	
		P: CGTATTACCGCGGCTGCTGGCAC	0.2	

[a] Universal ProbeLibrary probe (Roche).
[b] Described at Vilarrasa et al. (Vilarrasa et al., 2018).
[c] TAMRA TaqMan probe (Life Technologies) labeled with 6FAM.
[d] Described at Nadkarni et al. (Nadkarni et al., 2002). All other primers and probes were self-designed.

Supplementary Table S4. Annotation of the sequences queried against the metagenomes. The first column ("Annotation") contains the name of each protein family, as displayed in Figure 6. The second column ("L1") contains the main group to which each protein family belongs. Columns "L2" and "L3" contain the annotated name and accession number, respectively, of the representative sequence of each protein family. Last, column "L4" contains the accesion numbers of all sequences belonging to each protein family.

New_annotation	L1	L2	L3	L4
AHL-Synthases-1-HdtS	AHL-Synthases	putative acylhomoserine lactone synthase HdtS [Pseudomonas protegens CHA0]	gi|500239661|gb|AGL81819.1|	AEV60042.1 gi.500239661.gb.AGL AAG30826.1
AHL-Synthases-2-LuxM	AHL-Synthases	RecName: Full=Acyl-homoserine-lactone synthase; AltName: Full=Autoinducer synthesis protein LuxI	WP-048661067.1	WP-012600240.1 WP-050712378.1 WP-048614683.1 WP-048610660.1 WP-048609758.1 AAC36807.1 CDT89922.1 WP-048661067.1 WP-065779339.1 WP-025501628.1 WP-021823173.1 WP-005494702.1 WP-029857810.1 WP-020838754.1 WP-029798823.1
AHL-Synthases-3-LasI-like	AHL-Synthases	MULTISPECIES: N-3-oxohexanoyl-L-homoserine lactone synthase AinS [Aliivibrio]	WP-003534106.1	WP-003534106.1
AHL-Synthases-4-LasI	AHL-Synthases	acyl-homoserine-lactone synthase [Vibrio crassostreae]	WP-003083017.1	WP-003083017.1
AHL-Synthases-5-RhlI	AHL-Synthases	MULTISPECIES: acyl-homoserine-lactone synthase [Sinorhizobium]	sp|P54291.2|RHLI-PSEAE	sp.P54291.2.RHLI-PS
AHL-Synthases-6-GinI	AHL-Synthases	MULTISPECIES: acyl-homoserine-lactone synthase LasI [Pseudomonas]	BAG07415.1	BAG07415.1
AHL-Synthases-7-CepI	AHL-Synthases	RecName: Full=Acyl-homoserine-lactone synthase; AltName: Full=Autoinducer synthesis protein RhlI	AAG61123.1	WP-002000467.1 WP-002023245.1 WP-000387874.1 WP-106479630.1 AAG61123.1
AHL-Synthases-8-AurI	AHL-Synthases	AHL synthase [Komagataeibacter intermedius]	BAM94429.1	BAM94429.1
AHL-Synthases-9-EdwI	AHL-Synthases	AHL synthase [Burkholderia cepacia ATCC 25416]	AYI57645.1	sp.P52988.1.YENI-YE AAA82096.1 BAF31199.1 AKC96425.1 AYI57645.1
AHL-Synthases-10-LuxI	AHL-Synthases	AHL synthase [Pseudomonas chlororaphis subsp. aurantiaca]	sp|P35328.1|LUXJ-ALIF1	sp.P35328.1.LUXJ-AL AAA27552.1
AHL-Synthases-11-AinS	AHL-Synthases	AHL synthase [Edwardsiella ictaluri]	WP-038155219.1	WP-038155219.1
AHL-Receptors-1-LuxR	AHL-Receptor	LuxR, partial [Aliivibrio fischeri]	AAQ90229.1	AAQ90229.1
AHL-Receptors-2-LuxR	AHL-Receptor	LuxR, partial [Vibrio harveyi]	AAN86705.2	AAN86705.2
AHL-Receptors-3-LuxR	AHL-Receptor	MULTISPECIES: LuxR family transcriptional regulator [Enterobacteriaceae]	WP-001560555.1	WP-001560555.1
AHL-Receptors-4-SdiA	AHL-Receptor	MULTISPECIES: autoinducer binding domain-containing protein [Burkholderia]	WP-006766136.1	WP-006766136.1 WP-027789395.1 AIE47888.1 AIE47886.1 AIE47884.1 AAG61131.1 AAG61132.1 AAG61130.1 AAG61133.1
AHL-Receptors-5-LasR	AHL-Receptor	TPA: transcriptional regulator LasR [Pseudomonas aeruginosa]	HCH0449634.1	BAA06489.1 HCH0449634.1 WP-134292997.1
AHL-Receptors-6-RhlR	AHL-Receptor	MULTISPECIES: transcriptional regulator RhlR [Pseudomonas]	WP-003119559.1	BAA15736.1 ATI05176.1 NP-416426.1 WP-032641394.1 WP-001154267.1 WP-024155212.1 WP-001154265.1 WP-003119559.1 ACI42868.1
AHL-Receptors-7-AbaR	AHL-Receptor	MULTISPECIES: LuxR family transcriptional regulator AbaR [Acinetobacter]	WP-000446790.1	WP-000446790.1
AHL-Receptors-8-TraR	AHL-Receptor	RecName: Full=Transcriptional activator protein TraR	sp|P54294.1|TRAR-AGRFC	sp.P54294.1.TRAR-AG
AHL-Receptors-9-PluR	AHL-Receptor	orphan LuxR-type receptor PluR [Photorhabdus laumondii subsp. laumondii]	AGO97061.1	AGO97061.1
AHL-Receptors-10-LuxN	AHL-Receptor	HAI-1 autoinducer 1 hybrid sensor histidine kinase/phosphatase LuxN	SQA39244.1	SQA39244.1
Non-AHL-Synthases-1-DarABC	Non-AHL-Synthases	Photorhabdus asymbiotica subsp. asymbiotica ATCC 43949 darABC operon	KP258227.1	KP258227.1
Non-AHL-Synthases-2-PpyS	Non-AHL-Synthases	Photorhabdus temperata subsp. thracensis strain DSM 15199 photopyrone synthase gene	KF218577.1	KF218577.1
Non-AHL-Synthases-3-RpaI	Non-AHL-Synthases	p-coumaryl-homoserine lactone synthase	Q6NCZ6.1	Q6NCZ6.1
Non-AHL-Receptors-1-QscR	Non-AHL-Receptors	quorum-sensing control repressor	NP_250589.1	NP_250589.1
Non-AHL-Receptors-2-RpaR	Non-AHL-Receptors	4-coumaroyl-homoserine lactone receptor	Q6NCZ5.1	Q6NCZ5.1
Non-AHL-Receptors-3-VqmA	Non-AHL-Receptors	Chain A, VqmA	pdb|7DWM|A	pdb|7DWM|A
Non-AHL-Receptors-4-PhcR	Non-AHL-Receptors	hybrid sensor histidine kinase/response regulator [Ralstonia solanacearum]	WP_003262523.1	WP_003262523.1
Non-AHL-Receptors-5-OryR	Non-AHL-Receptors	Transcriptional regulator AhyR/AsaR family	CAH2707836.1	CAH2707836.1
PQS-Synthase-PqsABC	PQS-Synthase	Chain A, PQS biosynthetic enzyme	pdb|3H77|A	pdb|3H77|A
PQS-Receptor-PqsR	PQS-Receptor	transcriptional regulator PqsR	QDL04481.1	QDL04481.1
LuxS-1-*V._harveyi*	LuxS (AI-2-Synthase)	autoinducer-2 production protein LuxS [Vibrio harveyi]	AAD17292.1	AAD17292.1 ANW92499.1 NP-417172.1 AAR88507.1 CAL28543.1
LuxS-2-*S._pyogenes*	LuxS (AI-2-Synthase)	LuxS [Streptococcus pyogenes]	AAG28749.1	AAG28749.1 CDK34488.1
LuxS-3-*B._thuringiensis*	LuxS (AI-2-Synthase)	S-ribosylhomocysteine lyase LuxS [Bacillus thuringiensis]	OFC89811.1	OFC89811.1
LuxS-4-*B._heparinolyticus*	LuxS (AI-2-Synthase)	quorum-sensing autoinducer 2 (AI-2), LuxS [Bacteroides heparinolyticus]	VFB13531.1	WP-028584559.1 CDB53682.1 VFB13531.1
AI-2-Receptors-1-LsrR	AI-2-Receptors	Transcriptional regulator LsrR	Q8ZKQ5.1	Q8ZKQ5.1
AI-2-Receptors-2-LuxP	AI-2-Receptors	autoinducer 2-binding periplasmic protein LuxP	WP_137357207.1	WP_137357207.1

QQ-acylases-1-Aac	QQ-enzymes-acylases	acy-homoserine lactone acylase [Shewanella sp. MIB015]	BAF94155.1	BAF94155.1 WP-064663481.1
QQ-acylases-2-PfmA-MomL	QQ-enzymes-acylases	AhlA protein gene (translated)	ASS36259.1	ASS36259.1 KR232934.1 AKN24550.1 AKN24545.1 AKN24546.1 AKN24549.1 AKN24548.1 AKN24547.1 WP-034041734.1 AIY30473.1
QQ-acylases-3-PvdQ	QQ-enzymes-acylases	N-acylhomoserine lactone-degrading acylase [Streptomyces sp. M664]	NP-251075.1	NP-251075.1 NC-007005.1:2285834
QQ-acylases-4-QuiP-QlcA	QQ-enzymes-acylases	AHL acylase [[Ochrobactrum] quorumnocens]	AAG04421.1	AAG04421.1 ABV58973.1
QQ-acylases-5-PheA	QQ-enzymes-acylases	acylhomoserine lactone acylase [Acinetobacter sp. Ooi24]	KON65911.1	KON65911.1
QQ-acylases-6-HacB	QQ-enzymes-acylases	penicillin acylase family protein [Pseudoalteromonas sp. MQS005]	NC-007005.1:5764268-5766655	NC-007005.1:5764268 NP-248996.1
QQ-acylases-7-KcPGA	QQ-enzymes-acylases	N-Acyl-L-homoserine lactone acylase [Acidovorax sp. MR-S7]	WP-061283079.1	WP-061283079.1
QQ-acylases-8-Aibp	QQ-enzymes-acylases	AHL acylase, partial [Pseudoalteromonas flavipulchra JG1]	AAL53453.1	AAL53453.1
QQ-acylases-9-AiiC	QQ-enzymes-acylases	acyl-homoserine lactone acylase PvdQ [Pseudomonas aeruginosa PAO1]	WP-010998065.1	WP-010998065.1
QQ-acylases-10-AhlA	QQ-enzymes-acylases	QuiP [Pseudomonas aeruginosa PAO1]	EU839661.1:1174-2145	EU839661.1:1174-214
QQ-acylases-11-AhlM	QQ-enzymes-acylases	P-protein [Komagataeibacter europaeus]	AAT68473.1	WP-011002462.1 AAO41113.1 AAT68473.1
QQ-acylases-12-AiiO	QQ-enzymes-acylases	Pseudomonas syringae pv. syringae B728a (translated)	ADI80348.1	ADI80348.1
QQ-acylases-13-AmiE	QQ-enzymes-acylases	penicillin acylase family protein [Kluyvera cryocrescens]	BAP18758.1	BAP18758.1
QQ-acylases-14-APTM01	QQ-enzymes-acylases	penicillin acylase [Brucella melitensis bv. 1 str. 16M]	WP-064663184.1	WP-064663184.1
QQ-acylases-15-MacQ	QQ-enzymes-acylases	penicillin acylase family protein [Nostoc sp. PCC 7120]	BAV56778.1	BAV56778.1
QQ-lactonases-1-AidA	QQ-enzymes-lactonases	RecName: Full=Quorum-quenching protein AidA; AltName: Full=Quorum-quenching enzyme; Short=QQ enzyme	sp|G2JHL6.1|AIDA-ACIBM	sp.G2JHL6.1.AIDA-AC ODA53988.1
QQ-lactonases-3-Bpi04	QQ-enzymes-lactonases	cold-adapted N-acylhomoserine lactonase Aii810 [uncultured bacterium]	ABU51107.1	ABU51107.1
QQ-lactonases-4-Bpi05	QQ-enzymes-lactonases	N-acylhomoserine lactonase [Microbacterium testaceum StLB037]	ABU51109.1	ABU51109.1
QQ-lactonases-5-AidB12C	QQ-enzymes-lactonases	acyl homoserine lactone degrading enzyme [Arthrobacter sp. IBN110]	MN240477.1	MN240477.1
QQ-lactonases-6-CarA/CarB	QQ-enzymes-lactonases	Klebsiella pneumoniae AhlK (ahlK) gene (translated)	EU162747.1	EU162747.1
QQ-lactonases-7-RmmL	QQ-enzymes-lactonases	Mammaliicoccus sciuri ATCC 29060 ahlS gene for N-acylhomoserine lactonase (translated)	AYM45058.1	AYM45058.1
QQ-lactonases-8-Y2-AiiA	QQ-enzymes-lactonases	acylhomoserine lactonase [Chryseobacterium sp. StRB126]	KIQ72097.1	KIQ72097.1
QQ-lactonases-9-YtnP	QQ-enzymes-lactonases	putative metallohydrolase [Bacillus sp. 240B1]	KIQ73938.1	KIQ73938.1
QQ-lactonases-10-AidH	QQ-enzymes-lactonases	mlr6805 [Mesorhizobium japonicum MAFF 303099]	ACZ73823.1	ACZ73823.1
QQ-lactonases-11-Aii810	QQ-enzymes-lactonases	metallo-lactamase family protein [Muricauda olearia]	ASY06633.1	ASY06633.1
QQ-lactonases-12-AiiM	QQ-enzymes-lactonases	QlcA [uncultured Acidobacteria bacterium cosmid p2H8]	BAJ75775.1	BAJ75775.1
QQ-lactonases-13-AhlD	QQ-enzymes-lactonases	metal dependent hydrolase (plasmid) [Sinorhizobium fredii NGR234]	AAP57766.1	AAP57766.1
QQ-lactonases-14-AhlK	QQ-enzymes-lactonases	3-isopropylmalate dehydratase small subunit [Sphingomonas ursincola]	AY222324.1	AY222324.1 AWI14311.1 AY052389.1:3565-435
QQ-lactonases-15-AhlS	QQ-enzymes-lactonases	N-acyl homoserine lactonase family protein [Pseudorhizobium banfieldiae]	LC128625.1	AMS36875.1 BAK54003.1 LC128625.1 LC128626.1 LC128627.1 WP-049694637.1 BAO85041.1 sp.A9CKY2.1.AHLLB-A WP-021296945.1 WP-017434252.1
QQ-lactonases-16-AidC	QQ-enzymes-lactonases	Aryldialkylphosphatase [Sulfolobus islandicus M.16.4]	BAM28988.1	BAM28988.1
QQ-lactonases-17-AiiA	QQ-enzymes-lactonases	serum paraoxonase/arylesterase 1 precursor [Homo sapiens]	AAF62398.1	AAF62398.1 ADK91097.1 AHE80976.1 AHE80974.1 AKS43589.1 ALD49083.1 ALD49082.1 AHA91696.1 AHE80975.1 ALD49084.1 CAD44268.1
QQ-lactonases-18-mlr6805	QQ-enzymes-lactonases	Chain B, Organophosphorus hydrolase	BAB53031.1	BAB53031.1
QQ-lactonases-20-QsdR1	QQ-enzymes-lactonases	RND-type efflux transporter protein, partial [Pseudoalteromonas byunsanensis]	ACP23138.1	ACP23138.1
QQ-lactonases-21-QsdS	QQ-enzymes-lactonases	BpiB01 [uncultured bacterium Bio1]	BAV69341.1	BAV69341.1
QQ-lactonases-22-AHL-lactonase	QQ-enzymes-lactonases	BpiB04 [uncultured bacterium Bio7]	WP-052638829.1	WP-052638829.1
QQ-lactonases-23-SisLac	QQ-enzymes-lactonases	BpiB05 [uncultured bacterium Bio8]	ACR40964.1	ACR40964.1 sp.Q97VT7.1.PHP-SAC
QQ-lactonases-25-GsP	QQ-enzymes-lactonases	Pseudomonas sp. G carbamoyl-phosphate synthase small subunit (carA) and large subunit (carB) genes (translated)	pdb|3F4D|B	pdb.4H9U.A pdb.3F4D.B
QQ-lactonases-26-MCP	QQ-enzymes-lactonases	RmmL, partial [Tritonibacter mobilis]	AAS06218.1	AAS06218.1 ASZ24809.1 AAT06802.1 pdb.4RE0.A
QQ-lactonases-27-QsdH	QQ-enzymes-lactonases	N-acyl homoserine lactonase [Acinetobacter baumannii]	AFV15299.1	AFV15299.1
QQ-lactonases-28-Bpi01	QQ-enzymes-lactonases	putative quorum-quenching lactonase YtnP [Acinetobacter baumannii]	ABU51084.1	ABU51084.1
QQ-paraoxonases-PON	QQ-enzymes-lactonases	Bosea thiooxidans strain 12C N-acylhomoserine lactonase gene (translated)	NP-000437.3	NP-000437.3 NP-000296.2 NP-000931.1

Supplementary Table S5. Identification codes and accession links to the 118 Human Microbiome Project supragingival shotgun metagenomes used in this study.

SRS ID	GFF3 Download	GFF3 File Size	Nucleotide Multifasta Download	Nucl. File Size	Protein Multifasta Download	Protein File Size	https://downloads.hmpdacc.org/
SRS011343	/data/HMGI/supragingival_plaque/SRS011343.with_fasta.gff3.bz2	49400880	/data/HMGI/supragingival_plaque/SRS011343_nucleotide.fasta.bz2	24056666	/data/HMGI/supragingival_plaque/SRS011343_aa.fasta.bz2	15533954	https://downloads.hmpdacc.org//data/HMGI/supragingival_plaque/SRS011343_aa.fasta.bz2
SRS058808	/data/HMGI/supragingival_plaque/SRS058808.with_fasta.gff3.bz2	70325972	/data/HMGI/supragingival_plaque/SRS058808_nucleotide.fasta.bz2	34370510	/data/HMGI/supragingival_plaque/SRS058808_aa.fasta.bz2	22179417	https://downloads.hmpdacc.org//data/HMGI/supragingival_plaque/SRS058808_aa.fasta.bz2
SRS063603	/data/HMGI/supragingival_plaque/SRS063603.with_fasta.gff3.bz2	86435943	/data/HMGI/supragingival_plaque/SRS063603_nucleotide.fasta.bz2	41731925	/data/HMGI/supragingival_plaque/SRS063603_aa.fasta.bz2	26947524	https://downloads.hmpdacc.org//data/HMGI/supragingival_plaque/SRS063603_aa.fasta.bz2
SRS021960	/data/HMGI/supragingival_plaque/SRS021960.with_fasta.gff3.bz2	31873077	/data/HMGI/supragingival_plaque/SRS021960_nucleotide.fasta.bz2	15398083	/data/HMGI/supragingival_plaque/SRS021960_aa.fasta.bz2	9947254	https://downloads.hmpdacc.org//data/HMGI/supragingival_plaque/SRS021960_aa.fasta.bz2
SRS018975	/data/HMGI/supragingival_plaque/SRS018975.with_fasta.gff3.bz2	47288550	/data/HMGI/supragingival_plaque/SRS018975_nucleotide.fasta.bz2	23155080	/data/HMGI/supragingival_plaque/SRS018975_aa.fasta.bz2	14961582	https://downloads.hmpdacc.org//data/HMGI/supragingival_plaque/SRS018975_aa.fasta.bz2
SRS045197	/data/HMGI/supragingival_plaque/SRS045197.with_fasta.gff3.bz2	72557164	/data/HMGI/supragingival_plaque/SRS045197_nucleotide.fasta.bz2	34983192	/data/HMGI/supragingival_plaque/SRS045197_aa.fasta.bz2	22610992	https://downloads.hmpdacc.org//data/HMGI/supragingival_plaque/SRS045197_aa.fasta.bz2
SRS019591	/data/HMGI/supragingival_plaque/SRS019591.with_fasta.gff3.bz2	35635653	/data/HMGI/supragingival_plaque/SRS019591_nucleotide.fasta.bz2	17437708	/data/HMGI/supragingival_plaque/SRS019591_aa.fasta.bz2	11255925	https://downloads.hmpdacc.org//data/HMGI/supragingival_plaque/SRS019591_aa.fasta.bz2
SRS013723	/data/HMGI/supragingival_plaque/SRS013723.with_fasta.gff3.bz2	76743955	/data/HMGI/supragingival_plaque/SRS013723_nucleotide.fasta.bz2	37545560	/data/HMGI/supragingival_plaque/SRS013723_aa.fasta.bz2	24233551	https://downloads.hmpdacc.org//data/HMGI/supragingival_plaque/SRS013723_aa.fasta.bz2
SRS018394	/data/HMGI/supragingival_plaque/SRS018394.with_fasta.gff3.bz2	58790438	/data/HMGI/supragingival_plaque/SRS018394_nucleotide.fasta.bz2	28275716	/data/HMGI/supragingival_plaque/SRS018394_aa.fasta.bz2	18282199	https://downloads.hmpdacc.org//data/HMGI/supragingival_plaque/SRS018394_aa.fasta.bz2
SRS019906	/data/HMGI/supragingival_plaque/SRS019906.with_fasta.gff3.bz2	38232190	/data/HMGI/supragingival_plaque/SRS019906_nucleotide.fasta.bz2	18391799	/data/HMGI/supragingival_plaque/SRS019906_aa.fasta.bz2	11881119	https://downloads.hmpdacc.org//data/HMGI/supragingival_plaque/SRS019906_aa.fasta.bz2
SRS022725	/data/HMGI/supragingival_plaque/SRS022725.with_fasta.gff3.bz2	87729005	/data/HMGI/supragingival_plaque/SRS022725_nucleotide.fasta.bz2	42064483	/data/HMGI/supragingival_plaque/SRS022725_aa.fasta.bz2	27181685	https://downloads.hmpdacc.org//data/HMGI/supragingival_plaque/SRS022725_aa.fasta.bz2
SRS015215	/data/HMGI/supragingival_plaque/SRS015215.with_fasta.gff3.bz2	60278728	/data/HMGI/supragingival_plaque/SRS015215_nucleotide.fasta.bz2	29322503	/data/HMGI/supragingival_plaque/SRS015215_aa.fasta.bz2	18926637	https://downloads.hmpdacc.org//data/HMGI/supragingival_plaque/SRS015215_aa.fasta.bz2
SRS051244	/data/HMGI/supragingival_plaque/SRS051244.with_fasta.gff3.bz2	50750990	/data/HMGI/supragingival_plaque/SRS051244_nucleotide.fasta.bz2	24170650	/data/HMGI/supragingival_plaque/SRS051244_aa.fasta.bz2	15625541	https://downloads.hmpdacc.org//data/HMGI/supragingival_plaque/SRS051244_aa.fasta.bz2
SRS024087	/data/HMGI/supragingival_plaque/SRS024087.with_fasta.gff3.bz2	89344911	/data/HMGI/supragingival_plaque/SRS024087_nucleotide.fasta.bz2	43422885	/data/HMGI/supragingival_plaque/SRS024087_aa.fasta.bz2	28058404	https://downloads.hmpdacc.org//data/HMGI/supragingival_plaque/SRS024087_aa.fasta.bz2
SRS042984	/data/HMGI/supragingival_plaque/SRS042984.with_fasta.gff3.bz2	68268114	/data/HMGI/supragingival_plaque/SRS042984_nucleotide.fasta.bz2	33138092	/data/HMGI/supragingival_plaque/SRS042984_aa.fasta.bz2	21403998	https://downloads.hmpdacc.org//data/HMGI/supragingival_plaque/SRS042984_aa.fasta.bz2
SRS022149	/data/HMGI/supragingival_plaque/SRS022149.with_fasta.gff3.bz2	43413094	/data/HMGI/supragingival_plaque/SRS022149_nucleotide.fasta.bz2	20990677	/data/HMGI/supragingival_plaque/SRS022149_aa.fasta.bz2	13565501	https://downloads.hmpdacc.org//data/HMGI/supragingival_plaque/SRS022149_aa.fasta.bz2
SRS024355	/data/HMGI/supragingival_plaque/SRS024355.with_fasta.gff3.bz2	72255015	/data/HMGI/supragingival_plaque/SRS024355_nucleotide.fasta.bz2	35063223	/data/HMGI/supragingival_plaque/SRS024355_aa.fasta.bz2	22654425	https://downloads.hmpdacc.org//data/HMGI/supragingival_plaque/SRS024355_aa.fasta.bz2
SRS011152	/data/HMGI/supragingival_plaque/SRS011152.with_fasta.gff3.bz2	55240553	/data/HMGI/supragingival_plaque/SRS011152_nucleotide.fasta.bz2	26531824	/data/HMGI/supragingival_plaque/SRS011152_aa.fasta.bz2	17143659	https://downloads.hmpdacc.org//data/HMGI/supragingival_plaque/SRS011152_aa.fasta.bz2
SRS011098	/data/HMGI/supragingival_plaque/SRS011098.with_fasta.gff3.bz2	36378362	/data/HMGI/supragingival_plaque/SRS011098_nucleotide.fasta.bz2	17573729	/data/HMGI/supragingival_plaque/SRS011098_aa.fasta.bz2	11348789	https://downloads.hmpdacc.org//data/HMGI/supragingival_plaque/SRS011098_aa.fasta.bz2
SRS015574	/data/HMGI/supragingival_plaque/SRS015574.with_fasta.gff3.bz2	69434782	/data/HMGI/supragingival_plaque/SRS015574_nucleotide.fasta.bz2	33482107	/data/HMGI/supragingival_plaque/SRS015574_aa.fasta.bz2	21648548	https://downloads.hmpdacc.org//data/HMGI/supragingival_plaque/SRS015574_aa.fasta.bz2
SRS015803	/data/HMGI/supragingival_plaque/SRS015803.with_fasta.gff3.bz2	46240264	/data/HMGI/supragingival_plaque/SRS015803_nucleotide.fasta.bz2	22312591	/data/HMGI/supragingival_plaque/SRS015803_aa.fasta.bz2	14413502	https://downloads.hmpdacc.org//data/HMGI/supragingival_plaque/SRS015803_aa.fasta.bz2
SRS016575	/data/HMGI/supragingival_plaque/SRS016575.with_fasta.gff3.bz2	77233910	/data/HMGI/supragingival_plaque/SRS016575_nucleotide.fasta.bz2	37583653	/data/HMGI/supragingival_plaque/SRS016575_aa.fasta.bz2	24288328	https://downloads.hmpdacc.org//data/HMGI/supragingival_plaque/SRS016575_aa.fasta.bz2
SRS017511	/data/HMGI/supragingival_plaque/SRS017511.with_fasta.gff3.bz2	68450293	/data/HMGI/supragingival_plaque/SRS017511_nucleotide.fasta.bz2	33448872	/data/HMGI/supragingival_plaque/SRS017511_aa.fasta.bz2	21580265	https://downloads.hmpdacc.org//data/HMGI/supragingival_plaque/SRS017511_aa.fasta.bz2
SRS055401	/data/HMGI/supragingival_plaque/SRS055401.with_fasta.gff3.bz2	37688173	/data/HMGI/supragingival_plaque/SRS055401_nucleotide.fasta.bz2	17991423	/data/HMGI/supragingival_plaque/SRS055401_aa.fasta.bz2	11626898	https://downloads.hmpdacc.org//data/HMGI/supragingival_plaque/SRS055401_aa.fasta.bz2
SRS043772	/data/HMGI/supragingival_plaque/SRS043772.with_fasta.gff3.bz2	33089112	/data/HMGI/supragingival_plaque/SRS043772_nucleotide.fasta.bz2	16037383	/data/HMGI/supragingival_plaque/SRS043772_aa.fasta.bz2	10344895	https://downloads.hmpdacc.org//data/HMGI/supragingival_plaque/SRS043772_aa.fasta.bz2
SRS017227	/data/HMGI/supragingival_plaque/SRS017227.with_fasta.gff3.bz2	107896371	/data/HMGI/supragingival_plaque/SRS017227_nucleotide.fasta.bz2	52524967	/data/HMGI/supragingival_plaque/SRS017227_aa.fasta.bz2	33929692	https://downloads.hmpdacc.org//data/HMGI/supragingival_plaque/SRS017227_aa.fasta.bz2
SRS018778	/data/HMGI/supragingival_plaque/SRS018778.with_fasta.gff3.bz2	11025696	/data/HMGI/supragingival_plaque/SRS018778_nucleotide.fasta.bz2	5262750	/data/HMGI/supragingival_plaque/SRS018778_aa.fasta.bz2	3408315	https://downloads.hmpdacc.org//data/HMGI/supragingival_plaque/SRS018778_aa.fasta.bz2
SRS015378	/data/HMGI/supragingival_plaque/SRS015378.with_fasta.gff3.bz2	17906071	/data/HMGI/supragingival_plaque/SRS015378_nucleotide.fasta.bz2	8584048	/data/HMGI/supragingival_plaque/SRS015378_aa.fasta.bz2	5548645	https://downloads.hmpdacc.org//data/HMGI/supragingival_plaque/SRS015378_aa.fasta.bz2
SRS049318	/data/HMGI/supragingival_plaque/SRS049318.with_fasta.gff3.bz2	105310599	/data/HMGI/supragingival_plaque/SRS049318_nucleotide.fasta.bz2	50505204	/data/HMGI/supragingival_plaque/SRS049318_aa.fasta.bz2	32666587	https://downloads.hmpdacc.org//data/HMGI/supragingival_plaque/SRS049318_aa.fasta.bz2
SRS015044	/data/HMGI/supragingival_plaque/SRS015044.with_fasta.gff3.bz2	53943714	/data/HMGI/supragingival_plaque/SRS015044_nucleotide.fasta.bz2	25698099	/data/HMGI/supragingival_plaque/SRS015044_aa.fasta.bz2	16586250	https://downloads.hmpdacc.org//data/HMGI/supragingival_plaque/SRS015044_aa.fasta.bz2
SRS022083	/data/HMGI/supragingival_plaque/SRS022083.with_fasta.gff3.bz2	30895121	/data/HMGI/supragingival_plaque/SRS022083_nucleotide.fasta.bz2	15030264	/data/HMGI/supragingival_plaque/SRS022083_aa.fasta.bz2	9706431	https://downloads.hmpdacc.org//data/HMGI/supragingival_plaque/SRS022083_aa.fasta.bz2
SRS045313	/data/HMGI/supragingival_plaque/SRS045313.with_fasta.gff3.bz2	17352072	/data/HMGI/supragingival_plaque/SRS045313_nucleotide.fasta.bz2	8284115	/data/HMGI/supragingival_plaque/SRS045313_aa.fasta.bz2	5367111	https://downloads.hmpdacc.org//data/HMGI/supragingival_plaque/SRS045313_aa.fasta.bz2
SRS055378	/data/HMGI/supragingival_plaque/SRS055378.with_fasta.gff3.bz2	78813877	/data/HMGI/supragingival_plaque/SRS055378_nucleotide.fasta.bz2	37696568	/data/HMGI/supragingival_plaque/SRS055378_aa.fasta.bz2	24356348	https://downloads.hmpdacc.org//data/HMGI/supragingival_plaque/SRS055378_aa.fasta.bz2
SRS055450	/data/HMGI/supragingival_plaque/SRS055450.with_fasta.gff3.bz2	33951341	/data/HMGI/supragingival_plaque/SRS055450_nucleotide.fasta.bz2	16130019	/data/HMGI/supragingival_plaque/SRS055450_aa.fasta.bz2	10430511	https://downloads.hmpdacc.org//data/HMGI/supragingival_plaque/SRS055450_aa.fasta.bz2
SRS065310	/data/HMGI/supragingival_plaque/SRS065310.with_fasta.gff3.bz2	23335525	/data/HMGI/supragingival_plaque/SRS065310_nucleotide.fasta.bz2	11291039	/data/HMGI/supragingival_plaque/SRS065310_aa.fasta.bz2	7291830	https://downloads.hmpdacc.org//data/HMGI/supragingival_plaque/SRS065310_aa.fasta.bz2
SRS020226	/data/HMGI/supragingival_plaque/SRS020226.with_fasta.gff3.bz2	70021830	/data/HMGI/supragingival_plaque/SRS020226_nucleotide.fasta.bz2	34218763	/data/HMGI/supragingival_plaque/SRS020226_aa.fasta.bz2	22097600	https://downloads.hmpdacc.org//data/HMGI/supragingival_plaque/SRS020226_aa.fasta.bz2
SRS015470	/data/HMGI/supragingival_plaque/SRS015470.with_fasta.gff3.bz2	47737290	/data/HMGI/supragingival_plaque/SRS015470_nucleotide.fasta.bz2	23132639	/data/HMGI/supragingival_plaque/SRS015470_aa.fasta.bz2	14935801	https://downloads.hmpdacc.org//data/HMGI/supragingival_plaque/SRS015470_aa.fasta.bz2
SRS019128	/data/HMGI/supragingival_plaque/SRS019128.with_fasta.gff3.bz2	60563414	/data/HMGI/supragingival_plaque/SRS019128_nucleotide.fasta.bz2	28980841	/data/HMGI/supragingival_plaque/SRS019128_aa.fasta.bz2	18729367	https://downloads.hmpdacc.org//data/HMGI/supragingival_plaque/SRS019128_aa.fasta.bz2
SRS019333	/data/HMGI/supragingival_plaque/SRS019333.with_fasta.gff3.bz2	21124008	/data/HMGI/supragingival_plaque/SRS019333_nucleotide.fasta.bz2	10175093	/data/HMGI/supragingival_plaque/SRS019333_aa.fasta.bz2	6591347	https://downloads.hmpdacc.org//data/HMGI/supragingival_plaque/SRS019333_aa.fasta.bz2
SRS024289	/data/HMGI/supragingival_plaque/SRS024289.with_fasta.gff3.bz2	54766435	/data/HMGI/supragingival_plaque/SRS024289_nucleotide.fasta.bz2	26762830	/data/HMGI/supragingival_plaque/SRS024289_aa.fasta.bz2	17294654	https://downloads.hmpdacc.org//data/HMGI/supragingival_plaque/SRS024289_aa.fasta.bz2
SRS052604	/data/HMGI/supragingival_plaque/SRS052604.with_fasta.gff3.bz2	28001121	/data/HMGI/supragingival_plaque/SRS052604_nucleotide.fasta.bz2	13568030	/data/HMGI/supragingival_plaque/SRS052604_aa.fasta.bz2	8768584	https://downloads.hmpdacc.org//data/HMGI/supragingival_plaque/SRS052604_aa.fasta.bz2
SRS063999	/data/HMGI/supragingival_plaque/SRS063999.with_fasta.gff3.bz2	80148254	/data/HMGI/supragingival_plaque/SRS063999_nucleotide.fasta.bz2	38805781	/data/HMGI/supragingival_plaque/SRS063999_aa.fasta.bz2	25061238	https://downloads.hmpdacc.org//data/HMGI/supragingival_plaque/SRS063999_aa.fasta.bz2
SRS014894	/data/HMGI/supragingival_plaque/SRS014894.with_fasta.gff3.bz2	36414138	/data/HMGI/supragingival_plaque/SRS014894_nucleotide.fasta.bz2	17412555	/data/HMGI/supragingival_plaque/SRS014894_aa.fasta.bz2	11273350	https://downloads.hmpdacc.org//data/HMGI/supragingival_plaque/SRS014894_aa.fasta.bz2
SRS020862	/data/HMGI/supragingival_plaque/SRS020862.with_fasta.gff3.bz2	22641457	/data/HMGI/supragingival_plaque/SRS020862_nucleotide.fasta.bz2	11035582	/data/HMGI/supragingival_plaque/SRS020862_aa.fasta.bz2	7113420	https://downloads.hmpdacc.org//data/HMGI/supragingival_plaque/SRS020862_aa.fasta.bz2
SRS014690	/data/HMGI/supragingival_plaque/SRS014690.with_fasta.gff3.bz2	35340035	/data/HMGI/supragingival_plaque/SRS014690_nucleotide.fasta.bz2	17039847	/data/HMGI/supragingival_plaque/SRS014690_aa.fasta.bz2	11015845	https://downloads.hmpdacc.org//data/HMGI/supragingival_plaque/SRS014690_aa.fasta.bz2
SRS015440	/data/HMGI/supragingival_plaque/SRS015440.with_fasta.gff3.bz2	46519305	/data/HMGI/supragingival_plaque/SRS015440_nucleotide.fasta.bz2	22297666	/data/HMGI/supragingival_plaque/SRS015440_aa.fasta.bz2	14398487	https://downloads.hmpdacc.org//data/HMGI/supragingival_plaque/SRS015440_aa.fasta.bz2
SRS023358	/data/HMGI/supragingival_plaque/SRS023358.with_fasta.gff3.bz2	27211923	/data/HMGI/supragingival_plaque/SRS023358_nucleotide.fasta.bz2	12830631	/data/HMGI/supragingival_plaque/SRS023358_aa.fasta.bz2	8304175	https://downloads.hmpdacc.org//data/HMGI/supragingival_plaque/SRS023358_aa.fasta.bz2
SRS019077	/data/HMGI/supragingival_plaque/SRS019077.with_fasta.gff3.bz2	48008101	/data/HMGI/supragingival_plaque/SRS019077_nucleotide.fasta.bz2	22969414	/data/HMGI/supragingival_plaque/SRS019077_aa.fasta.bz2	14851091	https://downloads.hmpdacc.org//data/HMGI/supragingival_plaque/SRS019077_aa.fasta.bz2

SRS054430	/data/HMGI/supragingival_plaque/SRS054430.with_fasta.gff3.bz2	33399056	/data/HMGI/supragingival_plaque/SRS054430_nucleotide.fasta.bz2	15940236	/data/HMGI/supragingival_plaque/SRS054430_aa.fasta.bz2	10308526	https://downloads.hmpdacc.org//data/HMGI/supragingival_plaque/SRS054430_aa.fasta.bz2
SRS054653	/data/HMGI/supragingival_plaque/SRS054653.with_fasta.gff3.bz2	25910304	/data/HMGI/supragingival_plaque/SRS054653_nucleotide.fasta.bz2	12420476	/data/HMGI/supragingival_plaque/SRS054653_aa.fasta.bz2	8038310	https://downloads.hmpdacc.org//data/HMGI/supragingival_plaque/SRS054653_aa.fasta.bz2
SRS051378	/data/HMGI/supragingival_plaque/SRS051378.with_fasta.gff3.bz2	29771012	/data/HMGI/supragingival_plaque/SRS051378_nucleotide.fasta.bz2	14461972	/data/HMGI/supragingival_plaque/SRS051378_aa.fasta.bz2	9340557	https://downloads.hmpdacc.org//data/HMGI/supragingival_plaque/SRS051378_aa.fasta.bz2
SRS043755	/data/HMGI/supragingival_plaque/SRS043755.with_fasta.gff3.bz2	35672068	/data/HMGI/supragingival_plaque/SRS043755_nucleotide.fasta.bz2	17021459	/data/HMGI/supragingival_plaque/SRS043755_aa.fasta.bz2	10992707	https://downloads.hmpdacc.org//data/HMGI/supragingival_plaque/SRS043755_aa.fasta.bz2
SRS023595	/data/HMGI/supragingival_plaque/SRS023595.with_fasta.gff3.bz2	105352446	/data/HMGI/supragingival_plaque/SRS023595_nucleotide.fasta.bz2	51111927	/data/HMGI/supragingival_plaque/SRS023595_aa.fasta.bz2	33028014	https://downloads.hmpdacc.org//data/HMGI/supragingival_plaque/SRS023595_aa.fasta.bz2
SRS019028	/data/HMGI/supragingival_plaque/SRS019028.with_fasta.gff3.bz2	57747835	/data/HMGI/supragingival_plaque/SRS019028_nucleotide.fasta.bz2	27864881	/data/HMGI/supragingival_plaque/SRS019028_aa.fasta.bz2	18026559	https://downloads.hmpdacc.org//data/HMGI/supragingival_plaque/SRS019028_aa.fasta.bz2
SRS049268	/data/HMGI/supragingival_plaque/SRS049268.with_fasta.gff3.bz2	66429509	/data/HMGI/supragingival_plaque/SRS049268_nucleotide.fasta.bz2	32423796	/data/HMGI/supragingival_plaque/SRS049268_aa.fasta.bz2	20954810	https://downloads.hmpdacc.org//data/HMGI/supragingival_plaque/SRS049268_aa.fasta.bz2
SRS043018	/data/HMGI/supragingival_plaque/SRS043018.with_fasta.gff3.bz2	55499834	/data/HMGI/supragingival_plaque/SRS043018_nucleotide.fasta.bz2	26550313	/data/HMGI/supragingival_plaque/SRS043018_aa.fasta.bz2	17158357	https://downloads.hmpdacc.org//data/HMGI/supragingival_plaque/SRS043018_aa.fasta.bz2
SRS047113	/data/HMGI/supragingival_plaque/SRS047113.with_fasta.gff3.bz2	84841892	/data/HMGI/supragingival_plaque/SRS047113_nucleotide.fasta.bz2	40616750	/data/HMGI/supragingival_plaque/SRS047113_aa.fasta.bz2	26260899	https://downloads.hmpdacc.org//data/HMGI/supragingival_plaque/SRS047113_aa.fasta.bz2
SRS024447	/data/HMGI/supragingival_plaque/SRS024447.with_fasta.gff3.bz2	49500348	/data/HMGI/supragingival_plaque/SRS024447_nucleotide.fasta.bz2	23892148	/data/HMGI/supragingival_plaque/SRS024447_aa.fasta.bz2	15453781	https://downloads.hmpdacc.org//data/HMGI/supragingival_plaque/SRS024447_aa.fasta.bz2
SRS013252	/data/HMGI/supragingival_plaque/SRS013252.with_fasta.gff3.bz2	70112435	/data/HMGI/supragingival_plaque/SRS013252_nucleotide.fasta.bz2	34224652	/data/HMGI/supragingival_plaque/SRS013252_aa.fasta.bz2	22115169	https://downloads.hmpdacc.org//data/HMGI/supragingival_plaque/SRS013252_aa.fasta.bz2
SRS016043	/data/HMGI/supragingival_plaque/SRS016043.with_fasta.gff3.bz2	48913438	/data/HMGI/supragingival_plaque/SRS016043_nucleotide.fasta.bz2	23346687	/data/HMGI/supragingival_plaque/SRS016043_aa.fasta.bz2	15094176	https://downloads.hmpdacc.org//data/HMGI/supragingival_plaque/SRS016043_aa.fasta.bz2
SRS016200	/data/HMGI/supragingival_plaque/SRS016200.with_fasta.gff3.bz2	37749930	/data/HMGI/supragingival_plaque/SRS016200_nucleotide.fasta.bz2	18252117	/data/HMGI/supragingival_plaque/SRS016200_aa.fasta.bz2	11798789	https://downloads.hmpdacc.org//data/HMGI/supragingival_plaque/SRS016200_aa.fasta.bz2
SRS024021	/data/HMGI/supragingival_plaque/SRS024021.with_fasta.gff3.bz2	37064282	/data/HMGI/supragingival_plaque/SRS024021_nucleotide.fasta.bz2	18089502	/data/HMGI/supragingival_plaque/SRS024021_aa.fasta.bz2	11684759	https://downloads.hmpdacc.org//data/HMGI/supragingival_plaque/SRS024021_aa.fasta.bz2
SRS015755	/data/HMGI/supragingival_plaque/SRS015755.with_fasta.gff3.bz2	29573627	/data/HMGI/supragingival_plaque/SRS015755_nucleotide.fasta.bz2	13891551	/data/HMGI/supragingival_plaque/SRS015755_aa.fasta.bz2	8994903	https://downloads.hmpdacc.org//data/HMGI/supragingival_plaque/SRS015755_aa.fasta.bz2
SRS015947	/data/HMGI/supragingival_plaque/SRS015947.with_fasta.gff3.bz2	20762034	/data/HMGI/supragingival_plaque/SRS015947_nucleotide.fasta.bz2	9918249	/data/HMGI/supragingival_plaque/SRS015947_aa.fasta.bz2	6413397	https://downloads.hmpdacc.org//data/HMGI/supragingival_plaque/SRS015947_aa.fasta.bz2
SRS017139	/data/HMGI/supragingival_plaque/SRS017139.with_fasta.gff3.bz2	68808685	/data/HMGI/supragingival_plaque/SRS017139_nucleotide.fasta.bz2	33363946	/data/HMGI/supragingival_plaque/SRS017139_aa.fasta.bz2	21548779	https://downloads.hmpdacc.org//data/HMGI/supragingival_plaque/SRS017139_aa.fasta.bz2
SRS047100	/data/HMGI/supragingival_plaque/SRS047100.with_fasta.gff3.bz2	30713861	/data/HMGI/supragingival_plaque/SRS047100_nucleotide.fasta.bz2	14681767	/data/HMGI/supragingival_plaque/SRS047100_aa.fasta.bz2	9497025	https://downloads.hmpdacc.org//data/HMGI/supragingival_plaque/SRS047100_aa.fasta.bz2
SRS052876	/data/HMGI/supragingival_plaque/SRS052876.with_fasta.gff3.bz2	54689684	/data/HMGI/supragingival_plaque/SRS052876_nucleotide.fasta.bz2	26604195	/data/HMGI/supragingival_plaque/SRS052876_aa.fasta.bz2	17183356	https://downloads.hmpdacc.org//data/HMGI/supragingival_plaque/SRS052876_aa.fasta.bz2
SRS015158	/data/HMGI/supragingival_plaque/SRS015158.with_fasta.gff3.bz2	39321333	/data/HMGI/supragingival_plaque/SRS015158_nucleotide.fasta.bz2	18782120	/data/HMGI/supragingival_plaque/SRS015158_aa.fasta.bz2	12141632	https://downloads.hmpdacc.org//data/HMGI/supragingival_plaque/SRS015158_aa.fasta.bz2
SRS021477	/data/HMGI/supragingival_plaque/SRS021477.with_fasta.gff3.bz2	60535208	/data/HMGI/supragingival_plaque/SRS021477_nucleotide.fasta.bz2	29524210	/data/HMGI/supragingival_plaque/SRS021477_aa.fasta.bz2	19054937	https://downloads.hmpdacc.org//data/HMGI/supragingival_plaque/SRS021477_aa.fasta.bz2
SRS016092	/data/HMGI/supragingival_plaque/SRS016092.with_fasta.gff3.bz2	26011621	/data/HMGI/supragingival_plaque/SRS016092_nucleotide.fasta.bz2	12770162	/data/HMGI/supragingival_plaque/SRS016092_aa.fasta.bz2	8249251	https://downloads.hmpdacc.org//data/HMGI/supragingival_plaque/SRS016092_aa.fasta.bz2
SRS017814	/data/HMGI/supragingival_plaque/SRS017814.with_fasta.gff3.bz2	21480346	/data/HMGI/supragingival_plaque/SRS017814_nucleotide.fasta.bz2	10222084	/data/HMGI/supragingival_plaque/SRS017814_aa.fasta.bz2	6614906	https://downloads.hmpdacc.org//data/HMGI/supragingival_plaque/SRS017814_aa.fasta.bz2
SRS013533	/data/HMGI/supragingival_plaque/SRS013533.with_fasta.gff3.bz2	67064570	/data/HMGI/supragingival_plaque/SRS013533_nucleotide.fasta.bz2	32056930	/data/HMGI/supragingival_plaque/SRS013533_aa.fasta.bz2	20725137	https://downloads.hmpdacc.org//data/HMGI/supragingival_plaque/SRS013533_aa.fasta.bz2
SRS014578	/data/HMGI/supragingival_plaque/SRS014578.with_fasta.gff3.bz2	57262379	/data/HMGI/supragingival_plaque/SRS014578_nucleotide.fasta.bz2	27646311	/data/HMGI/supragingival_plaque/SRS014578_aa.fasta.bz2	17851113	https://downloads.hmpdacc.org//data/HMGI/supragingival_plaque/SRS014578_aa.fasta.bz2
SRS047634	/data/HMGI/supragingival_plaque/SRS047634.with_fasta.gff3.bz2	102060169	/data/HMGI/supragingival_plaque/SRS047634_nucleotide.fasta.bz2	50193628	/data/HMGI/supragingival_plaque/SRS047634_aa.fasta.bz2	32397322	https://downloads.hmpdacc.org//data/HMGI/supragingival_plaque/SRS047634_aa.fasta.bz2
SRS013836	/data/HMGI/supragingival_plaque/SRS013836.with_fasta.gff3.bz2	49753803	/data/HMGI/supragingival_plaque/SRS013836_nucleotide.fasta.bz2	24109187	/data/HMGI/supragingival_plaque/SRS013836_aa.fasta.bz2	15592427	https://downloads.hmpdacc.org//data/HMGI/supragingival_plaque/SRS013836_aa.fasta.bz2
SRS015063	/data/HMGI/supragingival_plaque/SRS015063.with_fasta.gff3.bz2	31191971	/data/HMGI/supragingival_plaque/SRS015063_nucleotide.fasta.bz2	15019623	/data/HMGI/supragingival_plaque/SRS015063_aa.fasta.bz2	9735000	https://downloads.hmpdacc.org//data/HMGI/supragingival_plaque/SRS015063_aa.fasta.bz2
SRS024649	/data/HMGI/supragingival_plaque/SRS024649.with_fasta.gff3.bz2	54500672	/data/HMGI/supragingival_plaque/SRS024649_nucleotide.fasta.bz2	26284424	/data/HMGI/supragingival_plaque/SRS024649_aa.fasta.bz2	16981013	https://downloads.hmpdacc.org//data/HMGI/supragingival_plaque/SRS024649_aa.fasta.bz2
SRS015989	/data/HMGI/supragingival_plaque/SRS015989.with_fasta.gff3.bz2	34714679	/data/HMGI/supragingival_plaque/SRS015989_nucleotide.fasta.bz2	17049242	/data/HMGI/supragingival_plaque/SRS015989_aa.fasta.bz2	11012369	https://downloads.hmpdacc.org//data/HMGI/supragingival_plaque/SRS015989_aa.fasta.bz2
SRS015899	/data/HMGI/supragingival_plaque/SRS015899.with_fasta.gff3.bz2	39387561	/data/HMGI/supragingival_plaque/SRS015899_nucleotide.fasta.bz2	19186843	/data/HMGI/supragingival_plaque/SRS015899_aa.fasta.bz2	12392353	https://downloads.hmpdacc.org//data/HMGI/supragingival_plaque/SRS015899_aa.fasta.bz2
SRS024144	/data/HMGI/supragingival_plaque/SRS024144.with_fasta.gff3.bz2	38079300	/data/HMGI/supragingival_plaque/SRS024144_nucleotide.fasta.bz2	18440916	/data/HMGI/supragingival_plaque/SRS024144_aa.fasta.bz2	11919265	https://downloads.hmpdacc.org//data/HMGI/supragingival_plaque/SRS024144_aa.fasta.bz2
SRS015650	/data/HMGI/supragingival_plaque/SRS015650.with_fasta.gff3.bz2	21566207	/data/HMGI/supragingival_plaque/SRS015650_nucleotide.fasta.bz2	10342015	/data/HMGI/supragingival_plaque/SRS015650_aa.fasta.bz2	6682427	https://downloads.hmpdacc.org//data/HMGI/supragingival_plaque/SRS015650_aa.fasta.bz2
SRS017691	/data/HMGI/supragingival_plaque/SRS017691.with_fasta.gff3.bz2	87314456	/data/HMGI/supragingival_plaque/SRS017691_nucleotide.fasta.bz2	42618486	/data/HMGI/supragingival_plaque/SRS017691_aa.fasta.bz2	27554847	https://downloads.hmpdacc.org//data/HMGI/supragingival_plaque/SRS017691_aa.fasta.bz2
SRS013170	/data/HMGI/supragingival_plaque/SRS013170.with_fasta.gff3.bz2	87273402	/data/HMGI/supragingival_plaque/SRS013170_nucleotide.fasta.bz2	42324745	/data/HMGI/supragingival_plaque/SRS013170_aa.fasta.bz2	27328918	https://downloads.hmpdacc.org//data/HMGI/supragingival_plaque/SRS013170_aa.fasta.bz2
SRS016360	/data/HMGI/supragingival_plaque/SRS016360.with_fasta.gff3.bz2	43940275	/data/HMGI/supragingival_plaque/SRS016360_nucleotide.fasta.bz2	21341351	/data/HMGI/supragingival_plaque/SRS016360_aa.fasta.bz2	13778033	https://downloads.hmpdacc.org//data/HMGI/supragingival_plaque/SRS016360_aa.fasta.bz2
SRS017304	/data/HMGI/supragingival_plaque/SRS017304.with_fasta.gff3.bz2	39371835	/data/HMGI/supragingival_plaque/SRS017304_nucleotide.fasta.bz2	19142501	/data/HMGI/supragingival_plaque/SRS017304_aa.fasta.bz2	12383412	https://downloads.hmpdacc.org//data/HMGI/supragingival_plaque/SRS017304_aa.fasta.bz2
SRS075410	/data/HMGI/supragingival_plaque/SRS075410.with_fasta.gff3.bz2	40246625	/data/HMGI/supragingival_plaque/SRS075410_nucleotide.fasta.bz2	19432633	/data/HMGI/supragingival_plaque/SRS075410_aa.fasta.bz2	12585739	https://downloads.hmpdacc.org//data/HMGI/supragingival_plaque/SRS075410_aa.fasta.bz2
SRS065099	/data/HMGI/supragingival_plaque/SRS065099.with_fasta.gff3.bz2	63115140	/data/HMGI/supragingival_plaque/SRS065099_nucleotide.fasta.bz2	30095045	/data/HMGI/supragingival_plaque/SRS065099_aa.fasta.bz2	19451487	https://downloads.hmpdacc.org//data/HMGI/supragingival_plaque/SRS065099_aa.fasta.bz2
SRS016746	/data/HMGI/supragingival_plaque/SRS016746.with_fasta.gff3.bz2	59263848	/data/HMGI/supragingival_plaque/SRS016746_nucleotide.fasta.bz2	28785928	/data/HMGI/supragingival_plaque/SRS016746_aa.fasta.bz2	18585397	https://downloads.hmpdacc.org//data/HMGI/supragingival_plaque/SRS016746_aa.fasta.bz2
SRS017445	/data/HMGI/supragingival_plaque/SRS017445.with_fasta.gff3.bz2	56691338	/data/HMGI/supragingival_plaque/SRS017445_nucleotide.fasta.bz2	27427650	/data/HMGI/supragingival_plaque/SRS017445_aa.fasta.bz2	17718375	https://downloads.hmpdacc.org//data/HMGI/supragingival_plaque/SRS017445_aa.fasta.bz2
SRS018157	/data/HMGI/supragingival_plaque/SRS018157.with_fasta.gff3.bz2	77324482	/data/HMGI/supragingival_plaque/SRS018157_nucleotide.fasta.bz2	37496197	/data/HMGI/supragingival_plaque/SRS018157_aa.fasta.bz2	24222613	https://downloads.hmpdacc.org//data/HMGI/supragingival_plaque/SRS018157_aa.fasta.bz2
SRS024561	/data/HMGI/supragingival_plaque/SRS024561.with_fasta.gff3.bz2	61574532	/data/HMGI/supragingival_plaque/SRS024561_nucleotide.fasta.bz2	29969719	/data/HMGI/supragingival_plaque/SRS024561_aa.fasta.bz2	19366527	https://downloads.hmpdacc.org//data/HMGI/supragingival_plaque/SRS024561_aa.fasta.bz2
SRS058053	/data/HMGI/supragingival_plaque/SRS058053.with_fasta.gff3.bz2	63861650	/data/HMGI/supragingival_plaque/SRS058053_nucleotide.fasta.bz2	30728377	/data/HMGI/supragingival_plaque/SRS058053_aa.fasta.bz2	19866537	https://downloads.hmpdacc.org//data/HMGI/supragingival_plaque/SRS058053_aa.fasta.bz2
SRS064493	/data/HMGI/supragingival_plaque/SRS064493.with_fasta.gff3.bz2	11017668	/data/HMGI/supragingival_plaque/SRS064493_nucleotide.fasta.bz2	5175116	/data/HMGI/supragingival_plaque/SRS064493_aa.fasta.bz2	3343408	https://downloads.hmpdacc.org//data/HMGI/supragingival_plaque/SRS064493_aa.fasta.bz2
SRS019225	/data/HMGI/supragingival_plaque/SRS019225.with_fasta.gff3.bz2	28602349	/data/HMGI/supragingival_plaque/SRS019225_nucleotide.fasta.bz2	13799525	/data/HMGI/supragingival_plaque/SRS019225_aa.fasta.bz2	8924756	https://downloads.hmpdacc.org//data/HMGI/supragingival_plaque/SRS019225_aa.fasta.bz2
SRS053584	/data/HMGI/supragingival_plaque/SRS053584.with_fasta.gff3.bz2	32117507	/data/HMGI/supragingival_plaque/SRS053584_nucleotide.fasta.bz2	15450579	/data/HMGI/supragingival_plaque/SRS053584_aa.fasta.bz2	9993373	https://downloads.hmpdacc.org//data/HMGI/supragingival_plaque/SRS053584_aa.fasta.bz2
SRS019980	/data/HMGI/supragingival_plaque/SRS019980.with_fasta.gff3.bz2	63566427	/data/HMGI/supragingival_plaque/SRS019980_nucleotide.fasta.bz2	31080409	/data/HMGI/supragingival_plaque/SRS019980_aa.fasta.bz2	20076145	https://downloads.hmpdacc.org//data/HMGI/supragingival_plaque/SRS019980_aa.fasta.bz2
SRS011255	/data/HMGI/supragingival_plaque/SRS011255.with_fasta.gff3.bz2	62253587	/data/HMGI/supragingival_plaque/SRS011255_nucleotide.fasta.bz2	30230063	/data/HMGI/supragingival_plaque/SRS011255_aa.fasta.bz2	19531877	https://downloads.hmpdacc.org//data/HMGI/supragingival_plaque/SRS011255_aa.fasta.bz2
SRS012285	/data/HMGI/supragingival_plaque/SRS012285.with_fasta.gff3.bz2	66542729	/data/HMGI/supragingival_plaque/SRS012285_nucleotide.fasta.bz2	32354754	/data/HMGI/supragingival_plaque/SRS012285_aa.fasta.bz2	20906920	https://downloads.hmpdacc.org//data/HMGI/supragingival_plaque/SRS012285_aa.fasta.bz2
SRS022536	/data/HMGI/supragingival_plaque/SRS022536.with_fasta.gff3.bz2	83143741	/data/HMGI/supragingival_plaque/SRS022536_nucleotide.fasta.bz2	39283662	/data/HMGI/supragingival_plaque/SRS022536_aa.fasta.bz2	25442865	https://downloads.hmpdacc.org//data/HMGI/supragingival_plaque/SRS022536_aa.fasta.bz2

SRS023964	/data/HMGI/supragingival_plaque/SRS023964.with_fasta.gff3.bz2	51151679	/data/HMGI/supragingival_plaque/SRS023964_nucleotide.fasta.bz2	25056970	/data/HMGI/supragingival_plaque/SRS023964_aa.fasta.bz2	16177051	https://downloads.hmpdacc.org//data/HMGI/supragingival_plaque/SRS023964_aa.fasta.bz2
SRS047265	/data/HMGI/supragingival_plaque/SRS047265.with_fasta.gff3.bz2	17963365	/data/HMGI/supragingival_plaque/SRS047265_nucleotide.fasta.bz2	8569520	/data/HMGI/supragingival_plaque/SRS047265_aa.fasta.bz2	5538921	https://downloads.hmpdacc.org//data/HMGI/supragingival_plaque/SRS047265_aa.fasta.bz2
SRS011126	/data/HMGI/supragingival_plaque/SRS011126.with_fasta.gff3.bz2	78571005	/data/HMGI/supragingival_plaque/SRS011126_nucleotide.fasta.bz2	37977000	/data/HMGI/supragingival_plaque/SRS011126_aa.fasta.bz2	24538729	https://downloads.hmpdacc.org//data/HMGI/supragingival_plaque/SRS011126_aa.fasta.bz2
SRS016331	/data/HMGI/supragingival_plaque/SRS016331.with_fasta.gff3.bz2	54003197	/data/HMGI/supragingival_plaque/SRS016331_nucleotide.fasta.bz2	25762238	/data/HMGI/supragingival_plaque/SRS016331_aa.fasta.bz2	16657310	https://downloads.hmpdacc.org//data/HMGI/supragingival_plaque/SRS016331_aa.fasta.bz2
SRS064449	/data/HMGI/supragingival_plaque/SRS064449.with_fasta.gff3.bz2	58870487	/data/HMGI/supragingival_plaque/SRS064449_nucleotide.fasta.bz2	28148246	/data/HMGI/supragingival_plaque/SRS064449_aa.fasta.bz2	18200364	https://downloads.hmpdacc.org//data/HMGI/supragingival_plaque/SRS064449_aa.fasta.bz2
SRS023538	/data/HMGI/supragingival_plaque/SRS023538.with_fasta.gff3.bz2	31029386	/data/HMGI/supragingival_plaque/SRS023538_nucleotide.fasta.bz2	15212997	/data/HMGI/supragingival_plaque/SRS023538_aa.fasta.bz2	9818990	https://downloads.hmpdacc.org//data/HMGI/supragingival_plaque/SRS023538_aa.fasta.bz2
SRS014476	/data/HMGI/supragingival_plaque/SRS014476.with_fasta.gff3.bz2	58040379	/data/HMGI/supragingival_plaque/SRS014476_nucleotide.fasta.bz2	27909746	/data/HMGI/supragingival_plaque/SRS014476_aa.fasta.bz2	18031353	https://downloads.hmpdacc.org//data/HMGI/supragingival_plaque/SRS014476_aa.fasta.bz2
SRS051941	/data/HMGI/supragingival_plaque/SRS051941.with_fasta.gff3.bz2	56978376	/data/HMGI/supragingival_plaque/SRS051941_nucleotide.fasta.bz2	27327060	/data/HMGI/supragingival_plaque/SRS051941_aa.fasta.bz2	17658963	https://downloads.hmpdacc.org//data/HMGI/supragingival_plaque/SRS051941_aa.fasta.bz2
SRS024381	/data/HMGI/supragingival_plaque/SRS024381.with_fasta.gff3.bz2	47161622	/data/HMGI/supragingival_plaque/SRS024381_nucleotide.fasta.bz2	22902937	/data/HMGI/supragingival_plaque/SRS024381_aa.fasta.bz2	14784128	https://downloads.hmpdacc.org//data/HMGI/supragingival_plaque/SRS024381_aa.fasta.bz2
SRS023938	/data/HMGI/supragingival_plaque/SRS023938.with_fasta.gff3.bz2	58556015	/data/HMGI/supragingival_plaque/SRS023938_nucleotide.fasta.bz2	28706534	/data/HMGI/supragingival_plaque/SRS023938_aa.fasta.bz2	18530422	https://downloads.hmpdacc.org//data/HMGI/supragingival_plaque/SRS023938_aa.fasta.bz2
SRS013949	/data/HMGI/supragingival_plaque/SRS013949.with_fasta.gff3.bz2	47539911	/data/HMGI/supragingival_plaque/SRS013949_nucleotide.fasta.bz2	22791462	/data/HMGI/supragingival_plaque/SRS013949_aa.fasta.bz2	14734569	https://downloads.hmpdacc.org//data/HMGI/supragingival_plaque/SRS013949_aa.fasta.bz2
SRS020340	/data/HMGI/supragingival_plaque/SRS020340.with_fasta.gff3.bz2	39940999	/data/HMGI/supragingival_plaque/SRS020340_nucleotide.fasta.bz2	19193006	/data/HMGI/supragingival_plaque/SRS020340_aa.fasta.bz2	12402837	https://downloads.hmpdacc.org//data/HMGI/supragingival_plaque/SRS020340_aa.fasta.bz2
SRS018337	/data/HMGI/supragingival_plaque/SRS018337.with_fasta.gff3.bz2	24472392	/data/HMGI/supragingival_plaque/SRS018337_nucleotide.fasta.bz2	11703725	/data/HMGI/supragingival_plaque/SRS018337_aa.fasta.bz2	7571983	https://downloads.hmpdacc.org//data/HMGI/supragingival_plaque/SRS018337_aa.fasta.bz2
SRS015278	/data/HMGI/supragingival_plaque/SRS015278.with_fasta.gff3.bz2	41403619	/data/HMGI/supragingival_plaque/SRS015278_nucleotide.fasta.bz2	19275561	/data/HMGI/supragingival_plaque/SRS015278_aa.fasta.bz2	12470416	https://downloads.hmpdacc.org//data/HMGI/supragingival_plaque/SRS015278_aa.fasta.bz2
SRS017025	/data/HMGI/supragingival_plaque/SRS017025.with_fasta.gff3.bz2	32762543	/data/HMGI/supragingival_plaque/SRS017025_nucleotide.fasta.bz2	15851166	/data/HMGI/supragingival_plaque/SRS017025_aa.fasta.bz2	10241045	https://downloads.hmpdacc.org//data/HMGI/supragingival_plaque/SRS017025_aa.fasta.bz2
SRS051930	/data/HMGI/supragingival_plaque/SRS051930.with_fasta.gff3.bz2	59172345	/data/HMGI/supragingival_plaque/SRS051930_nucleotide.fasta.bz2	28732149	/data/HMGI/supragingival_plaque/SRS051930_aa.fasta.bz2	18557027	https://downloads.hmpdacc.org//data/HMGI/supragingival_plaque/SRS051930_aa.fasta.bz2
SRS018573	/data/HMGI/supragingival_plaque/SRS018573.with_fasta.gff3.bz2	42839980	/data/HMGI/supragingival_plaque/SRS018573_nucleotide.fasta.bz2	20696157	/data/HMGI/supragingival_plaque/SRS018573_aa.fasta.bz2	13364234	https://downloads.hmpdacc.org//data/HMGI/supragingival_plaque/SRS018573_aa.fasta.bz2
SRS018665	/data/HMGI/supragingival_plaque/SRS018665.with_fasta.gff3.bz2	68388031	/data/HMGI/supragingival_plaque/SRS018665_nucleotide.fasta.bz2	33029501	/data/HMGI/supragingival_plaque/SRS018665_aa.fasta.bz2	21327764	https://downloads.hmpdacc.org//data/HMGI/supragingival_plaque/SRS018665_aa.fasta.bz2
SRS019387	/data/HMGI/supragingival_plaque/SRS019387.with_fasta.gff3.bz2	30174461	/data/HMGI/supragingival_plaque/SRS019387_nucleotide.fasta.bz2	14535625	/data/HMGI/supragingival_plaque/SRS019387_aa.fasta.bz2	9383307	https://downloads.hmpdacc.org//data/HMGI/supragingival_plaque/SRS019387_aa.fasta.bz2

Supplementary Table S6. Accession numbers of the genomic sequences used to build the synthetic subgingival metagenome.

**Species representative of the subgingival niche**	**Accession number**
Actinomyces oris strain FDAARGOS_1051	GCF_016127955.1
Aggregatibacter actinomycetemcomitans strain 4S	GCF_023518055.1
Campylobacter gracilis ATCC 33236	GCF_001190745.1
Capnocytophaga ochracea DSM 7271	GCF_000023285.1
Corynebacterium durum F0235	GCF_000318135.1
Fusobacterium nucleatum subsp. *polymorphum* NCTC10562	GCF_001457555.1
Haemophilus influenzae strain 477	GCF_000931575.1
Kingella oralis DSM 18271	GCF_014054985.1
Lautropia mirabilis NCTC12852	GCF_900637555.1
Leptotrichia buccalis C-1013-b	GCF_000023905.1
Neisseria mucosa NCTC 10774	GCF_900454435.1
Porphyromonas endodontalis NCTC13058	GCF_900454815.1
Porphyromonas gingivalis ATCC 33277	GCF_000010505.1
Prevotella intermedia ATCC 25611	GCF_001953955.1
Rothia dentocariosa ATCC 17931	GCF_000164695.2
Rothia dentocariosa NCTC10207	GCF_900637985.1
Rothia mucilaginosa ATCC 25296	GCF_000175615.1
Selenomonas noxia F0398	GCF_000234135.1
Streptococcus constellatus NCTC11325	GCF_900459125.1
Streptococcus oralis ATCC 35037	GCF_900637025.1
Treponema denticola ATCC 35405	GCF_000008185.1
Veillonella parvula NCTC11810	GCF_900186885.1

Supplementary Table S7. Proportion (in percentage) of the bacterial genera present in supragingival and subgingival sites, based on the 16S rRNA gene information available in the Human Microbiome Project database.

Bacterial genera	Supragingival plaque (%)	Subgingival plaque (%)
Fusobacterium	5.1473649	11.0306199
Streptococcus	14.3459463	11.0177072
Actinomyces	9.4572169	8.0431097
Capnocytophaga	9.3199440	8.0406768
Corynebacterium	10.2235033	7.3093319
Prevotella	2.1991420	6.9427238
Neisseria	8.2208355	5.5408546
Veillonella	4.5956831	4.3777767
Leptotrichia	4.0932124	3.3846223
Rothia	4.9891865	3.3415800
Porphyromonas	2.7972597	3.2023475
Haemophilus	5.3980452	3.0346696
Lautropia	3.2964004	2.3076290
Selenomonas	1.1919804	2.2707623
Treponema	0.3487324	2.0424509
Campylobacter	1.0295470	1.4699883
Kingella	1.8552196	1.3339372
uncultured TM7	1.0104916	1.2755494
Aggregatibacter	1.5653184	1.2560867
Propionibacterium	1.2593219	1.2049974
Alloprevotella	0.4301342	0.9839845
Bergeyella	1.3508988	0.9658319
Paludibacter	0.2386551	0.9214796
Candidate	0.5979328	0.8191138
Actinobaculum	0.7102302	0.6671557
Lactobacillus	0.0234955	0.6417046
Eikenella	0.5043208	0.4949865
Dialister	0.0997171	0.4523185
Tannerella	0.1724237	0.3946792
Lachnoanaerobaculum	0.3505825	0.3765266
Granulicatella	0.4593648	0.3639882
Incertae	0.1628035	0.3364785
Cardiobacterium	0.3085866	0.2663009
Comamonas	0.1672436	0.2537625
Catonella	0.0899119	0.2386041
Johnsonella	0.1585484	0.2105330
Abiotrophia	0.3531725	0.2075388
Staphylococcus	0.0919470	0.1796548
Atopobium	0.0284906	0.1102257
Oribacterium	0.0294156	0.1047987
Actinobacillus	0.0958320	0.1008687
Gracilibacteria	0.1224726	0.0903888

Quorum jupyter notebook-2023

January 16, 2023

[2]:
```
library(phyloseq)
library(ggplot2)
library('ggrepel')
library('vegan')
library('RColorBrewer')
```

```
Loading required package: permute

Loading required package: lattice

This is vegan 2.6-4
```

[3]:
```
otu <- as.data.frame(read.table("tbout2023.clstr.tsv", header=T, row.names=1))
print(otu)
```

```
                        ca02246 NCA001 NCA003 NCA004 NCA008 NOCA002P RL7A1e
AHL-Receptors-4               0      0      0      0      0        0      0
AHL-Receptors-6               0      0      0      0      0        0      0
AHL-Receptors-7               0      0      0      0      0        0      0
AHL-Synthases-1               0      1      0      0      0       14      0
LuxS-1                        0      2      2      1      0       48      0
LuxS-2                        0      3      1      1      0       26      0
LuxS-3                        0      3      1      2      0       28      1
LuxS-4                        0      1      1      0      0        1      0
QQ-enzymes-acylases-1         0      0      1      0      1        0      0
QQ-enzymes-acylases-3         0      0      0      0      0        0      0
QQ-enzymes-acylases-4         0      0      0      0      0        0      0
QQ-enzymes-acylases-5         0      1      0      0      1        7      1
QQ-enzymes-acylases-6         0      0      0      0      0        0      0
QQ-enzymes-acylases-7         0      0      0      0      0        0      0
QQ-enzymes-acylases-8         0      0      0      0      0        0      0
QQ-enzymes-acylases-9         0      0      0      0      0        0      0
QQ-enzymes-acylases-10        0      0      0      0      0        0      0
QQ-enzymes-acylases-11        0      0      1      0      1        0      0
QQ-enzymes-acylases-12        0      0      0      0      0        0      0
QQ-enzymes-acylases-13        0      0      0      0      0        0      0
QQ-enzymes-acylases-14        0      0      0      0      0        0      0
```

QQ-enzymes-acylases-15	0	0	0	0	0	0	0
QQ-enzymes-lactonases-1	0	1	2	2	2	16	3
QQ-enzymes-lactonases-6	0	0	0	0	0	0	0
QQ-enzymes-lactonases-7	0	0	0	0	0	0	0
QQ-enzymes-lactonases-8	0	0	0	0	0	0	0
QQ-enzymes-lactonases-9	0	0	0	0	0	0	0
QQ-enzymes-lactonases-13	0	0	0	0	0	0	0
QQ-enzymes-lactonases-14	0	1	2	2	2	16	3
QQ-enzymes-lactonases-15	0	0	0	0	0	0	0
QQ-enzymes-lactonases-16	0	0	0	0	0	0	0
QQ-enzymes-lactonases-18	0	0	0	0	0	0	0
QQ-enzymes-lactonases-19	0	0	0	0	0	0	0
QQ-enzymes-lactonases-20	0	1	2	4	1	0	2
QQ-enzymes-lactonases-24	0	0	0	0	0	0	0
QQ-enzymes-lactonases-25	0	10	7	15	6	163	12
QQ-enzymes-lactonases-26	0	0	0	0	0	0	0
QQ-enzymes-lactonases-27	0	0	0	0	0	0	0
QQ-enzymes-lactonases-28	0	0	0	0	0	0	0
DarABC-1	0	1	0	1	1	0	0
LsrR-1	0	0	0	0	0	0	0
LuxN-1	0	0	1	0	0	0	0
PqsABC-1	0	2	2	1	2	17	3
QscR-1	0	0	0	0	0	0	0
RpaR-1	0	0	0	0	0	0	0

	SRS011098	SRS011126	SRS011152	SRS011255	SRS011343
AHL-Receptors-4	1	0	1	1	1
AHL-Receptors-6	2	3	2	2	2
AHL-Receptors-7	3	1	2	2	2
AHL-Synthases-1	3	5	4	5	2
LuxS-1	10	21	14	12	16
LuxS-2	8	16	15	10	11
LuxS-3	7	15	11	12	14
LuxS-4	10	14	9	13	7
QQ-enzymes-acylases-1	7	6	12	9	10
QQ-enzymes-acylases-3	0	2	0	0	0
QQ-enzymes-acylases-4	0	0	0	0	0
QQ-enzymes-acylases-5	3	12	9	6	12
QQ-enzymes-acylases-6	1	2	1	0	0
QQ-enzymes-acylases-7	0	2	0	0	0
QQ-enzymes-acylases-8	0	1	2	0	1
QQ-enzymes-acylases-9	0	2	0	0	0
QQ-enzymes-acylases-10	1	2	1	2	1
QQ-enzymes-acylases-11	5	1	7	7	8
QQ-enzymes-acylases-12	0	1	3	0	0
QQ-enzymes-acylases-13	0	1	2	0	0
QQ-enzymes-acylases-14	2	1	0	1	1
QQ-enzymes-acylases-15	2	4	5	2	2
QQ-enzymes-lactonases-1	17	18	8	21	14

QQ-enzymes-lactonases-6	0	2	0	0	0
QQ-enzymes-lactonases-7	0	0	0	0	0
QQ-enzymes-lactonases-8	0	1	1	2	0
QQ-enzymes-lactonases-9	0	0	0	0	0
QQ-enzymes-lactonases-13	0	0	1	2	0
QQ-enzymes-lactonases-14	16	13	6	17	13
QQ-enzymes-lactonases-15	0	1	0	0	0
QQ-enzymes-lactonases-16	1	2	0	1	1
QQ-enzymes-lactonases-18	1	3	1	1	0
QQ-enzymes-lactonases-19	1	1	0	1	0
QQ-enzymes-lactonases-20	19	23	13	25	20
QQ-enzymes-lactonases-24	1	2	1	1	0
QQ-enzymes-lactonases-25	59	133	89	116	88
QQ-enzymes-lactonases-26	3	4	2	3	3
QQ-enzymes-lactonases-27	0	0	0	0	0
QQ-enzymes-lactonases-28	3	5	3	3	2
DarABC-1	3	10	4	8	6
LsrR-1	3	5	5	4	7
LuxN-1	0	1	2	1	1
PqsABC-1	14	29	13	18	18
QscR-1	0	1	0	0	0
RpaR-1	1	1	2	2	2

	SRS012285	SRS013170	SRS013252	SRS013533	SRS013723
AHL-Receptors-4	0	1	0	1	1
AHL-Receptors-6	2	2	0	1	3
AHL-Receptors-7	2	2	0	2	3
AHL-Synthases-1	6	3	4	1	6
LuxS-1	23	17	19	11	26
LuxS-2	17	13	18	11	23
LuxS-3	16	14	10	14	20
LuxS-4	14	24	19	15	23
QQ-enzymes-acylases-1	11	15	17	8	16
QQ-enzymes-acylases-3	0	0	0	0	0
QQ-enzymes-acylases-4	0	0	0	0	0
QQ-enzymes-acylases-5	7	10	16	7	13
QQ-enzymes-acylases-6	0	0	0	1	1
QQ-enzymes-acylases-7	0	0	0	0	0
QQ-enzymes-acylases-8	1	1	2	2	3
QQ-enzymes-acylases-9	0	0	0	0	0
QQ-enzymes-acylases-10	2	1	1	0	2
QQ-enzymes-acylases-11	6	9	12	5	11
QQ-enzymes-acylases-12	1	1	1	1	2
QQ-enzymes-acylases-13	0	0	0	0	0
QQ-enzymes-acylases-14	1	1	0	1	1
QQ-enzymes-acylases-15	4	6	4	3	4
QQ-enzymes-lactonases-1	19	23	25	5	19
QQ-enzymes-lactonases-6	1	1	0	1	2
QQ-enzymes-lactonases-7	0	0	0	0	0

QQ-enzymes-lactonases-8	0	2	1	0	2
QQ-enzymes-lactonases-9	0	0	0	0	0
QQ-enzymes-lactonases-13	0	1	1	0	1
QQ-enzymes-lactonases-14	16	20	20	5	14
QQ-enzymes-lactonases-15	0	0	0	0	1
QQ-enzymes-lactonases-16	2	0	2	0	1
QQ-enzymes-lactonases-18	2	2	3	0	2
QQ-enzymes-lactonases-19	1	2	2	0	1
QQ-enzymes-lactonases-20	19	31	27	17	27
QQ-enzymes-lactonases-24	1	1	0	2	1
QQ-enzymes-lactonases-25	112	164	125	98	138
QQ-enzymes-lactonases-26	3	3	4	2	6
QQ-enzymes-lactonases-27	1	0	1	1	0
QQ-enzymes-lactonases-28	2	3	3	0	3
DarABC-1	10	11	6	4	10
LsrR-1	6	9	8	4	7
LuxN-1	3	2	2	0	1
PqsABC-1	22	33	26	20	27
QscR-1	0	0	0	0	0
RpaR-1	0	1	0	1	3

	SRS013836	SRS013949	SRS014476	SRS014578	SRS014690
AHL-Receptors-4	0	0	2	1	0
AHL-Receptors-6	0	1	3	2	0
AHL-Receptors-7	0	1	2	2	0
AHL-Synthases-1	1	4	4	6	1
LuxS-1	6	12	13	12	12
LuxS-2	5	10	7	12	10
LuxS-3	6	12	10	14	10
LuxS-4	15	10	11	9	10
QQ-enzymes-acylases-1	5	11	10	6	13
QQ-enzymes-acylases-3	0	0	1	0	0
QQ-enzymes-acylases-4	0	0	0	0	0
QQ-enzymes-acylases-5	6	8	10	10	8
QQ-enzymes-acylases-6	0	0	1	0	0
QQ-enzymes-acylases-7	0	0	1	0	0
QQ-enzymes-acylases-8	0	1	0	0	4
QQ-enzymes-acylases-9	0	0	1	0	0
QQ-enzymes-acylases-10	0	1	2	0	0
QQ-enzymes-acylases-11	5	6	6	5	8
QQ-enzymes-acylases-12	0	1	1	0	3
QQ-enzymes-acylases-13	0	0	0	0	0
QQ-enzymes-acylases-14	0	0	1	0	0
QQ-enzymes-acylases-15	0	4	2	1	5
QQ-enzymes-lactonases-1	8	17	11	22	11
QQ-enzymes-lactonases-6	1	1	1	0	1
QQ-enzymes-lactonases-7	0	0	0	0	0
QQ-enzymes-lactonases-8	0	1	3	4	1
QQ-enzymes-lactonases-9	0	0	0	0	0

QQ-enzymes-lactonases-13	0	1	0	1	0
QQ-enzymes-lactonases-14	6	12	11	19	10
QQ-enzymes-lactonases-15	1	0	0	0	0
QQ-enzymes-lactonases-16	1	1	0	2	0
QQ-enzymes-lactonases-18	0	3	0	1	1
QQ-enzymes-lactonases-19	0	2	0	1	1
QQ-enzymes-lactonases-20	14	10	15	20	7
QQ-enzymes-lactonases-24	0	1	1	1	0
QQ-enzymes-lactonases-25	102	66	92	92	62
QQ-enzymes-lactonases-26	1	2	2	2	2
QQ-enzymes-lactonases-27	1	0	0	0	0
QQ-enzymes-lactonases-28	0	3	2	3	3
DarABC-1	1	6	6	8	4
LsrR-1	2	5	1	4	3
LuxN-1	0	1	2	2	0
PqsABC-1	15	9	17	17	9
QscR-1	0	0	1	0	0
RpaR-1	0	1	2	2	0
	SRS014894	SRS015044	SRS015063	SRS015158	SRS015215
AHL-Receptors-4	0	1	4	0	0
AHL-Receptors-6	3	0	4	0	0
AHL-Receptors-7	1	1	5	0	0
AHL-Synthases-1	6	3	6	2	1
LuxS-1	7	13	8	7	19
LuxS-2	7	13	7	5	17
LuxS-3	7	13	10	6	13
LuxS-4	2	13	2	5	16
QQ-enzymes-acylases-1	6	9	4	7	10
QQ-enzymes-acylases-3	0	0	1	0	0
QQ-enzymes-acylases-4	0	0	0	0	0
QQ-enzymes-acylases-5	5	10	4	2	10
QQ-enzymes-acylases-6	0	0	0	0	0
QQ-enzymes-acylases-7	0	0	1	0	0
QQ-enzymes-acylases-8	1	1	0	0	0
QQ-enzymes-acylases-9	0	0	1	0	0
QQ-enzymes-acylases-10	0	1	1	0	0
QQ-enzymes-acylases-11	3	5	1	3	9
QQ-enzymes-acylases-12	0	2	0	0	0
QQ-enzymes-acylases-13	0	0	0	0	0
QQ-enzymes-acylases-14	0	2	0	0	0
QQ-enzymes-acylases-15	3	3	2	4	1
QQ-enzymes-lactonases-1	13	13	15	13	11
QQ-enzymes-lactonases-6	0	1	1	2	0
QQ-enzymes-lactonases-7	0	0	0	0	0
QQ-enzymes-lactonases-8	2	2	2	1	1
QQ-enzymes-lactonases-9	0	0	0	0	0
QQ-enzymes-lactonases-13	1	1	1	0	1
QQ-enzymes-lactonases-14	11	10	13	13	9

QQ-enzymes-lactonases-15	0	0	0	0	0
QQ-enzymes-lactonases-16	0	1	1	0	1
QQ-enzymes-lactonases-18	1	1	1	0	1
QQ-enzymes-lactonases-19	0	0	0	0	1
QQ-enzymes-lactonases-20	8	19	10	11	21
QQ-enzymes-lactonases-24	1	0	2	0	0
QQ-enzymes-lactonases-25	74	101	50	68	125
QQ-enzymes-lactonases-26	3	3	3	2	1
QQ-enzymes-lactonases-27	1	1	0	1	0
QQ-enzymes-lactonases-28	3	1	3	3	0
DarABC-1	8	5	5	6	6
LsrR-1	2	2	2	2	3
LuxN-1	1	1	0	2	0
PqsABC-1	10	12	10	9	16
QscR-1	0	0	1	0	0
RpaR-1	0	0	4	0	0

	SRS015278	SRS015378	SRS015440	SRS015470	SRS015574
AHL-Receptors-4	0	0	3	3	0
AHL-Receptors-6	0	0	3	4	0
AHL-Receptors-7	0	0	4	5	0
AHL-Synthases-1	1	2	3	3	7
LuxS-1	7	5	12	16	14
LuxS-2	8	3	15	14	12
LuxS-3	10	7	14	16	12
LuxS-4	10	2	8	11	17
QQ-enzymes-acylases-1	4	5	14	12	13
QQ-enzymes-acylases-3	0	0	1	1	0
QQ-enzymes-acylases-4	0	0	0	0	0
QQ-enzymes-acylases-5	6	3	7	12	9
QQ-enzymes-acylases-6	0	0	1	2	1
QQ-enzymes-acylases-7	0	0	1	1	0
QQ-enzymes-acylases-8	0	1	3	3	2
QQ-enzymes-acylases-9	0	0	1	1	0
QQ-enzymes-acylases-10	1	0	1	2	2
QQ-enzymes-acylases-11	3	2	8	6	9
QQ-enzymes-acylases-12	0	1	3	2	2
QQ-enzymes-acylases-13	0	1	1	1	0
QQ-enzymes-acylases-14	0	1	1	3	1
QQ-enzymes-acylases-15	0	3	5	4	3
QQ-enzymes-lactonases-1	16	6	16	15	15
QQ-enzymes-lactonases-6	1	2	2	2	3
QQ-enzymes-lactonases-7	0	0	0	0	0
QQ-enzymes-lactonases-8	2	0	2	2	1
QQ-enzymes-lactonases-9	0	0	0	0	0
QQ-enzymes-lactonases-13	2	0	1	1	0
QQ-enzymes-lactonases-14	13	6	13	13	13
QQ-enzymes-lactonases-15	0	0	0	0	1
QQ-enzymes-lactonases-16	0	0	1	0	1

QQ-enzymes-lactonases-18	1	0	1	1	1
QQ-enzymes-lactonases-19	0	0	1	1	1
QQ-enzymes-lactonases-20	11	2	11	14	26
QQ-enzymes-lactonases-24	0	0	1	2	0
QQ-enzymes-lactonases-25	78	39	71	79	126
QQ-enzymes-lactonases-26	3	1	1	1	3
QQ-enzymes-lactonases-27	0	0	1	1	0
QQ-enzymes-lactonases-28	2	0	3	2	2
DarABC-1	3	1	6	5	9
LsrR-1	4	2	5	7	7
LuxN-1	2	0	1	3	0
PqsABC-1	10	2	13	16	20
QscR-1	0	0	1	1	0
RpaR-1	0	0	3	4	0

	SRS015650	SRS015755	SRS015803	SRS015899	SRS015947
AHL-Receptors-4	1	0	0	0	0
AHL-Receptors-6	2	0	0	1	0
AHL-Receptors-7	2	0	0	1	0
AHL-Synthases-1	3	3	5	5	2
LuxS-1	6	4	17	13	4
LuxS-2	7	5	12	8	6
LuxS-3	8	4	14	13	6
LuxS-4	3	5	14	8	4
QQ-enzymes-acylases-1	1	2	8	7	4
QQ-enzymes-acylases-3	0	0	0	0	0
QQ-enzymes-acylases-4	0	0	1	0	0
QQ-enzymes-acylases-5	3	1	3	3	6
QQ-enzymes-acylases-6	0	0	1	0	0
QQ-enzymes-acylases-7	0	0	0	0	0
QQ-enzymes-acylases-8	0	0	3	0	0
QQ-enzymes-acylases-9	0	0	0	0	0
QQ-enzymes-acylases-10	0	2	0	1	2
QQ-enzymes-acylases-11	1	0	4	5	2
QQ-enzymes-acylases-12	0	0	2	0	0
QQ-enzymes-acylases-13	0	0	0	0	0
QQ-enzymes-acylases-14	0	1	2	0	1
QQ-enzymes-acylases-15	0	2	4	2	1
QQ-enzymes-lactonases-1	10	9	11	15	10
QQ-enzymes-lactonases-6	0	1	1	2	0
QQ-enzymes-lactonases-7	0	0	0	0	0
QQ-enzymes-lactonases-8	1	1	0	1	1
QQ-enzymes-lactonases-9	0	1	0	0	0
QQ-enzymes-lactonases-13	1	1	0	0	0
QQ-enzymes-lactonases-14	9	7	10	13	9
QQ-enzymes-lactonases-15	0	0	0	0	1
QQ-enzymes-lactonases-16	0	0	0	0	0
QQ-enzymes-lactonases-18	0	1	1	2	0
QQ-enzymes-lactonases-19	0	0	0	1	0

QQ-enzymes-lactonases-20	12	13	12	14	8
QQ-enzymes-lactonases-24	1	0	0	2	0
QQ-enzymes-lactonases-25	52	50	59	66	43
QQ-enzymes-lactonases-26	2	3	2	4	0
QQ-enzymes-lactonases-27	1	1	0	0	0
QQ-enzymes-lactonases-28	1	4	2	3	0
DarABC-1	4	2	10	7	3
LsrR-1	0	1	2	2	1
LuxN-1	0	1	2	0	0
PqsABC-1	6	5	18	16	6
QscR-1	0	0	0	0	0
RpaR-1	2	0	0	0	0

	SRS015989	SRS016043	SRS016092	SRS016200	SRS016331
AHL-Receptors-4	0	1	0	1	1
AHL-Receptors-6	0	1	1	1	0
AHL-Receptors-7	0	3	1	1	2
AHL-Synthases-1	4	5	4	5	3
LuxS-1	11	15	9	8	6
LuxS-2	11	12	9	8	3
LuxS-3	12	12	12	12	6
LuxS-4	5	11	4	4	8
QQ-enzymes-acylases-1	4	4	5	10	9
QQ-enzymes-acylases-3	0	0	0	0	0
QQ-enzymes-acylases-4	0	0	0	0	0
QQ-enzymes-acylases-5	6	9	3	10	15
QQ-enzymes-acylases-6	0	0	0	0	0
QQ-enzymes-acylases-7	0	0	0	0	0
QQ-enzymes-acylases-8	1	0	2	0	0
QQ-enzymes-acylases-9	0	0	0	0	0
QQ-enzymes-acylases-10	1	0	0	0	0
QQ-enzymes-acylases-11	1	3	3	6	7
QQ-enzymes-acylases-12	1	0	2	0	0
QQ-enzymes-acylases-13	0	0	1	0	0
QQ-enzymes-acylases-14	1	1	1	0	0
QQ-enzymes-acylases-15	3	1	2	4	2
QQ-enzymes-lactonases-1	12	18	9	12	9
QQ-enzymes-lactonases-6	1	1	0	2	2
QQ-enzymes-lactonases-7	0	0	0	0	0
QQ-enzymes-lactonases-8	2	2	1	3	0
QQ-enzymes-lactonases-9	0	0	0	0	0
QQ-enzymes-lactonases-13	1	2	0	1	0
QQ-enzymes-lactonases-14	9	15	8	10	7
QQ-enzymes-lactonases-15	0	0	0	0	0
QQ-enzymes-lactonases-16	1	1	0	0	1
QQ-enzymes-lactonases-18	2	1	1	1	1
QQ-enzymes-lactonases-19	2	1	1	1	0
QQ-enzymes-lactonases-20	17	17	13	14	10
QQ-enzymes-lactonases-24	0	1	0	1	0

QQ-enzymes-lactonases-25	77	78	40	75	89
QQ-enzymes-lactonases-26	3	4	0	2	0
QQ-enzymes-lactonases-27	1	1	0	1	1
QQ-enzymes-lactonases-28	2	4	1	2	0
DarABC-1	6	1	3	4	7
LsrR-1	3	2	0	6	3
LuxN-1	0	1	0	0	3
PqsABC-1	14	12	15	13	11
QscR-1	0	0	0	0	0
RpaR-1	0	1	1	1	0

	SRS016360	SRS016575	SRS016746	SRS017025	SRS017139
AHL-Receptors-4	1	1	0	0	2
AHL-Receptors-6	2	0	0	2	2
AHL-Receptors-7	2	2	0	1	3
AHL-Synthases-1	6	9	4	4	3
LuxS-1	15	22	12	11	14
LuxS-2	13	17	11	13	13
LuxS-3	13	19	10	17	13
LuxS-4	10	16	13	6	10
QQ-enzymes-acylases-1	3	15	7	3	12
QQ-enzymes-acylases-3	0	0	0	0	0
QQ-enzymes-acylases-4	0	0	0	0	0
QQ-enzymes-acylases-5	7	14	9	6	11
QQ-enzymes-acylases-6	0	0	0	0	0
QQ-enzymes-acylases-7	0	0	0	0	0
QQ-enzymes-acylases-8	0	2	0	0	0
QQ-enzymes-acylases-9	0	0	0	0	0
QQ-enzymes-acylases-10	0	0	1	0	1
QQ-enzymes-acylases-11	1	11	6	2	8
QQ-enzymes-acylases-12	0	1	0	0	1
QQ-enzymes-acylases-13	0	0	0	0	0
QQ-enzymes-acylases-14	0	1	0	0	0
QQ-enzymes-acylases-15	2	4	1	1	3
QQ-enzymes-lactonases-1	9	21	14	13	15
QQ-enzymes-lactonases-6	0	2	1	2	1
QQ-enzymes-lactonases-7	0	0	1	0	0
QQ-enzymes-lactonases-8	2	3	1	2	1
QQ-enzymes-lactonases-9	0	0	0	0	0
QQ-enzymes-lactonases-13	0	2	0	2	0
QQ-enzymes-lactonases-14	7	17	13	9	12
QQ-enzymes-lactonases-15	0	0	0	1	0
QQ-enzymes-lactonases-16	1	2	1	0	2
QQ-enzymes-lactonases-18	2	2	0	1	3
QQ-enzymes-lactonases-19	2	2	0	0	3
QQ-enzymes-lactonases-20	15	26	18	12	20
QQ-enzymes-lactonases-24	0	1	0	1	1
QQ-enzymes-lactonases-25	70	140	95	60	132
QQ-enzymes-lactonases-26	4	5	2	2	2

QQ-enzymes-lactonases-27	0	1	1	0	0
QQ-enzymes-lactonases-28	3	3	1	2	1
DarABC-1	10	8	7	6	10
LsrR-1	1	5	6	1	7
LuxN-1	2	1	0	1	2
PqsABC-1	19	33	18	8	29
QscR-1	0	0	0	0	1
RpaR-1	2	0	0	1	2

	SRS017227	SRS017304	SRS017445	SRS017511	SRS017691
AHL-Receptors-4	4	3	1	1	1
AHL-Receptors-6	6	4	2	2	2
AHL-Receptors-7	6	4	2	2	2
AHL-Synthases-1	7	4	6	4	5
LuxS-1	24	15	17	12	12
LuxS-2	19	12	16	7	11
LuxS-3	23	13	15	10	14
LuxS-4	32	8	13	16	24
QQ-enzymes-acylases-1	18	10	14	10	14
QQ-enzymes-acylases-3	1	2	0	0	0
QQ-enzymes-acylases-4	0	0	0	0	0
QQ-enzymes-acylases-5	18	7	12	11	11
QQ-enzymes-acylases-6	0	0	0	1	0
QQ-enzymes-acylases-7	1	2	0	0	0
QQ-enzymes-acylases-8	3	0	3	2	2
QQ-enzymes-acylases-9	1	2	0	0	0
QQ-enzymes-acylases-10	0	1	2	2	0
QQ-enzymes-acylases-11	11	7	6	6	11
QQ-enzymes-acylases-12	2	0	2	1	1
QQ-enzymes-acylases-13	1	0	0	0	0
QQ-enzymes-acylases-14	1	1	2	1	0
QQ-enzymes-acylases-15	6	2	6	4	3
QQ-enzymes-lactonases-1	29	22	22	21	21
QQ-enzymes-lactonases-6	2	0	1	1	1
QQ-enzymes-lactonases-7	0	0	0	0	0
QQ-enzymes-lactonases-8	3	2	2	2	1
QQ-enzymes-lactonases-9	0	1	0	0	0
QQ-enzymes-lactonases-13	1	1	1	1	0
QQ-enzymes-lactonases-14	25	21	17	16	19
QQ-enzymes-lactonases-15	0	0	0	0	1
QQ-enzymes-lactonases-16	2	0	2	3	0
QQ-enzymes-lactonases-18	2	0	3	3	1
QQ-enzymes-lactonases-19	2	0	1	2	0
QQ-enzymes-lactonases-20	38	12	19	33	25
QQ-enzymes-lactonases-24	1	1	1	1	0
QQ-enzymes-lactonases-25	175	85	96	106	147
QQ-enzymes-lactonases-26	3	3	2	2	1
QQ-enzymes-lactonases-27	0	0	0	1	0
QQ-enzymes-lactonases-28	4	3	3	2	3

DarABC-1	15	4	7	8	10
LsrR-1	10	3	9	5	4
LuxN-1	1	0	1	3	1
PqsABC-1	46	12	23	22	29
QscR-1	1	1	0	0	0
RpaR-1	4	4	2	2	2

	SRS017814	SRS018157	SRS018337	SRS018394	SRS018573
AHL-Receptors-4	2	1	0	1	1
AHL-Receptors-6	4	3	0	1	1
AHL-Receptors-7	3	1	0	2	1
AHL-Synthases-1	3	6	3	4	2
LuxS-1	4	18	11	14	12
LuxS-2	2	10	5	8	11
LuxS-3	3	13	8	9	14
LuxS-4	2	14	4	12	9
QQ-enzymes-acylases-1	0	8	2	5	6
QQ-enzymes-acylases-3	0	1	0	0	0
QQ-enzymes-acylases-4	0	0	0	0	0
QQ-enzymes-acylases-5	1	12	7	9	7
QQ-enzymes-acylases-6	0	0	0	0	0
QQ-enzymes-acylases-7	0	2	0	0	0
QQ-enzymes-acylases-8	0	2	0	0	0
QQ-enzymes-acylases-9	0	2	0	0	0
QQ-enzymes-acylases-10	0	1	0	1	2
QQ-enzymes-acylases-11	0	3	2	2	3
QQ-enzymes-acylases-12	0	2	0	0	0
QQ-enzymes-acylases-13	0	1	0	0	0
QQ-enzymes-acylases-14	0	1	0	0	0
QQ-enzymes-acylases-15	0	4	0	3	1
QQ-enzymes-lactonases-1	6	19	10	16	9
QQ-enzymes-lactonases-6	1	2	2	0	2
QQ-enzymes-lactonases-7	0	0	0	0	0
QQ-enzymes-lactonases-8	2	3	0	1	1
QQ-enzymes-lactonases-9	1	0	0	0	0
QQ-enzymes-lactonases-13	1	2	0	0	1
QQ-enzymes-lactonases-14	5	15	10	15	7
QQ-enzymes-lactonases-15	0	0	0	0	0
QQ-enzymes-lactonases-16	0	1	0	0	0
QQ-enzymes-lactonases-18	0	2	0	1	1
QQ-enzymes-lactonases-19	0	1	0	1	0
QQ-enzymes-lactonases-20	4	22	5	22	16
QQ-enzymes-lactonases-24	2	2	0	1	1
QQ-enzymes-lactonases-25	52	108	49	99	82
QQ-enzymes-lactonases-26	3	6	2	3	5
QQ-enzymes-lactonases-27	1	1	0	1	0
QQ-enzymes-lactonases-28	3	3	2	3	5
DarABC-1	3	15	1	11	8
LsrR-1	1	7	2	3	5

LuxN-1	0	3	0	0	2
PqsABC-1	5	26	7	16	14
QscR-1	0	1	0	0	0
RpaR-1	0	1	0	0	0
	SRS018665	SRS018778	SRS018975	SRS019028	SRS019077
AHL-Receptors-4	1	0	1	0	0
AHL-Receptors-6	1	0	2	0	1
AHL-Receptors-7	2	0	2	0	2
AHL-Synthases-1	8	1	7	2	3
LuxS-1	20	2	16	18	11
LuxS-2	16	2	14	13	13
LuxS-3	16	3	18	13	13
LuxS-4	15	1	12	13	11
QQ-enzymes-acylases-1	15	1	7	9	10
QQ-enzymes-acylases-3	0	0	0	0	0
QQ-enzymes-acylases-4	0	0	0	0	0
QQ-enzymes-acylases-5	11	1	6	10	9
QQ-enzymes-acylases-6	1	0	0	1	1
QQ-enzymes-acylases-7	0	0	0	0	0
QQ-enzymes-acylases-8	5	0	0	2	2
QQ-enzymes-acylases-9	0	0	0	0	0
QQ-enzymes-acylases-10	1	0	2	0	1
QQ-enzymes-acylases-11	6	1	5	5	5
QQ-enzymes-acylases-12	5	0	0	3	1
QQ-enzymes-acylases-13	1	0	0	0	0
QQ-enzymes-acylases-14	2	0	0	0	2
QQ-enzymes-acylases-15	8	0	2	3	5
QQ-enzymes-lactonases-1	12	5	19	8	12
QQ-enzymes-lactonases-6	0	1	1	0	1
QQ-enzymes-lactonases-7	0	0	0	0	0
QQ-enzymes-lactonases-8	2	0	2	2	1
QQ-enzymes-lactonases-9	0	0	0	0	0
QQ-enzymes-lactonases-13	1	0	1	0	0
QQ-enzymes-lactonases-14	9	5	15	7	10
QQ-enzymes-lactonases-15	0	0	0	0	0
QQ-enzymes-lactonases-16	0	0	2	1	2
QQ-enzymes-lactonases-18	2	0	3	1	1
QQ-enzymes-lactonases-19	0	0	2	1	1
QQ-enzymes-lactonases-20	20	5	22	19	18
QQ-enzymes-lactonases-24	1	0	1	0	1
QQ-enzymes-lactonases-25	104	25	80	83	87
QQ-enzymes-lactonases-26	3	0	3	2	2
QQ-enzymes-lactonases-27	0	0	1	0	1
QQ-enzymes-lactonases-28	3	0	3	2	3
DarABC-1	11	2	8	3	11
LsrR-1	6	0	5	1	3
LuxN-1	0	0	1	1	2
PqsABC-1	23	3	22	20	14

QscR-1	0	0	0	0	0
RpaR-1	1	0	2	0	0

	SRS019128	SRS019225	SRS019333	SRS019387	SRS019591
AHL-Receptors-4	0	0	0	0	0
AHL-Receptors-6	0	2	1	0	0
AHL-Receptors-7	1	1	1	0	0
AHL-Synthases-1	4	3	2	3	3
LuxS-1	11	6	3	8	12
LuxS-2	9	7	5	7	18
LuxS-3	10	8	7	9	15
LuxS-4	10	6	4	5	8
QQ-enzymes-acylases-1	8	3	4	8	8
QQ-enzymes-acylases-3	0	0	0	0	0
QQ-enzymes-acylases-4	0	0	0	0	0
QQ-enzymes-acylases-5	7	4	5	2	8
QQ-enzymes-acylases-6	0	0	0	0	1
QQ-enzymes-acylases-7	0	0	0	0	0
QQ-enzymes-acylases-8	1	0	0	0	2
QQ-enzymes-acylases-9	0	0	0	0	0
QQ-enzymes-acylases-10	0	1	1	3	1
QQ-enzymes-acylases-11	3	2	2	5	4
QQ-enzymes-acylases-12	1	0	0	0	1
QQ-enzymes-acylases-13	0	0	0	0	0
QQ-enzymes-acylases-14	0	0	0	1	0
QQ-enzymes-acylases-15	5	1	2	2	3
QQ-enzymes-lactonases-1	13	12	10	15	15
QQ-enzymes-lactonases-6	1	0	3	1	2
QQ-enzymes-lactonases-7	0	0	0	0	0
QQ-enzymes-lactonases-8	1	2	1	0	1
QQ-enzymes-lactonases-9	0	0	0	0	0
QQ-enzymes-lactonases-13	0	2	1	0	0
QQ-enzymes-lactonases-14	9	9	9	14	14
QQ-enzymes-lactonases-15	0	0	0	0	0
QQ-enzymes-lactonases-16	2	0	0	0	1
QQ-enzymes-lactonases-18	4	1	0	1	1
QQ-enzymes-lactonases-19	3	0	0	0	1
QQ-enzymes-lactonases-20	18	8	4	7	10
QQ-enzymes-lactonases-24	0	2	2	0	0
QQ-enzymes-lactonases-25	105	67	48	69	68
QQ-enzymes-lactonases-26	1	2	4	1	2
QQ-enzymes-lactonases-27	0	0	0	0	0
QQ-enzymes-lactonases-28	2	2	4	0	1
DarABC-1	12	4	3	3	4
LsrR-1	5	5	2	5	2
LuxN-1	2	0	0	0	1
PqsABC-1	15	11	5	3	12
QscR-1	0	0	0	0	0
RpaR-1	0	0	0	0	0

	SRS019906	SRS019980	SRS020226	SRS020340	SRS020862
AHL-Receptors-4	0	1	1	1	0
AHL-Receptors-6	0	2	1	2	0
AHL-Receptors-7	0	2	2	2	0
AHL-Synthases-1	6	4	5	1	2
LuxS-1	8	16	18	13	7
LuxS-2	6	19	16	14	10
LuxS-3	6	17	13	16	8
LuxS-4	8	16	17	10	4
QQ-enzymes-acylases-1	4	12	9	10	6
QQ-enzymes-acylases-3	0	0	0	0	0
QQ-enzymes-acylases-4	0	0	0	0	0
QQ-enzymes-acylases-5	6	12	14	6	4
QQ-enzymes-acylases-6	0	0	0	1	0
QQ-enzymes-acylases-7	0	0	0	0	0
QQ-enzymes-acylases-8	0	2	0	0	0
QQ-enzymes-acylases-9	0	0	0	0	0
QQ-enzymes-acylases-10	0	0	0	3	0
QQ-enzymes-acylases-11	3	9	8	6	5
QQ-enzymes-acylases-12	0	1	1	0	0
QQ-enzymes-acylases-13	0	0	0	0	0
QQ-enzymes-acylases-14	0	0	0	1	1
QQ-enzymes-acylases-15	1	3	0	4	1
QQ-enzymes-lactonases-1	10	16	21	9	9
QQ-enzymes-lactonases-6	0	1	1	1	1
QQ-enzymes-lactonases-7	0	0	0	0	0
QQ-enzymes-lactonases-8	3	2	2	1	0
QQ-enzymes-lactonases-9	0	0	0	0	0
QQ-enzymes-lactonases-13	0	1	2	1	0
QQ-enzymes-lactonases-14	9	13	16	7	9
QQ-enzymes-lactonases-15	0	0	1	0	0
QQ-enzymes-lactonases-16	1	1	1	0	0
QQ-enzymes-lactonases-18	1	2	2	1	0
QQ-enzymes-lactonases-19	1	1	2	1	0
QQ-enzymes-lactonases-20	13	34	25	11	10
QQ-enzymes-lactonases-24	0	1	1	1	0
QQ-enzymes-lactonases-25	52	123	105	85	51
QQ-enzymes-lactonases-26	2	3	2	2	1
QQ-enzymes-lactonases-27	0	0	0	0	1
QQ-enzymes-lactonases-28	1	3	2	1	0
DarABC-1	8	9	8	3	3
LsrR-1	1	3	7	3	2
LuxN-1	0	1	1	0	0
PqsABC-1	10	28	23	7	7
QscR-1	0	0	0	0	0
RpaR-1	0	2	0	1	0

	SRS021477	SRS021960	SRS022083	SRS022149	SRS022536
AHL-Receptors-4	2	0	0	0	0

AHL-Receptors-6	3	0	2	0	0
AHL-Receptors-7	3	0	2	0	0
AHL-Synthases-1	5	3	2	1	3
LuxS-1	15	7	8	5	21
LuxS-2	16	7	9	6	22
LuxS-3	16	9	8	6	22
LuxS-4	9	7	6	9	20
QQ-enzymes-acylases-1	9	5	7	8	16
QQ-enzymes-acylases-3	0	0	0	0	0
QQ-enzymes-acylases-4	0	0	0	0	0
QQ-enzymes-acylases-5	14	7	5	9	12
QQ-enzymes-acylases-6	1	0	0	0	0
QQ-enzymes-acylases-7	0	0	0	0	0
QQ-enzymes-acylases-8	0	0	0	0	2
QQ-enzymes-acylases-9	0	0	0	0	0
QQ-enzymes-acylases-10	2	0	2	0	1
QQ-enzymes-acylases-11	6	4	5	8	11
QQ-enzymes-acylases-12	0	0	0	0	1
QQ-enzymes-acylases-13	0	0	0	0	1
QQ-enzymes-acylases-14	3	0	1	0	1
QQ-enzymes-acylases-15	3	1	1	0	5
QQ-enzymes-lactonases-1	18	10	9	10	20
QQ-enzymes-lactonases-6	3	1	0	0	1
QQ-enzymes-lactonases-7	0	0	0	0	0
QQ-enzymes-lactonases-8	1	0	1	0	1
QQ-enzymes-lactonases-9	0	1	0	0	0
QQ-enzymes-lactonases-13	1	0	0	0	1
QQ-enzymes-lactonases-14	16	9	9	8	15
QQ-enzymes-lactonases-15	0	0	0	0	1
QQ-enzymes-lactonases-16	0	0	0	1	1
QQ-enzymes-lactonases-18	1	1	0	2	2
QQ-enzymes-lactonases-19	0	0	0	1	3
QQ-enzymes-lactonases-20	27	22	10	10	33
QQ-enzymes-lactonases-24	1	0	1	1	1
QQ-enzymes-lactonases-25	113	61	60	82	124
QQ-enzymes-lactonases-26	2	0	5	1	4
QQ-enzymes-lactonases-27	1	0	0	0	1
QQ-enzymes-lactonases-28	2	0	2	2	4
DarABC-1	4	4	4	0	10
LsrR-1	8	2	2	4	6
LuxN-1	0	0	1	1	1
PqsABC-1	24	10	8	7	25
QscR-1	1	0	0	0	0
RpaR-1	2	0	0	0	0

	SRS022725	SRS023358	SRS023538	SRS023595	SRS023938
AHL-Receptors-4	1	0	0	1	1
AHL-Receptors-6	2	0	0	1	2
AHL-Receptors-7	2	0	0	2	2

AHL-Synthases-1	5	1	2	7	3
LuxS-1	22	12	13	16	17
LuxS-2	17	10	13	14	16
LuxS-3	19	11	15	15	15
LuxS-4	20	8	7	22	15
QQ-enzymes-acylases-1	13	4	5	18	5
QQ-enzymes-acylases-3	0	0	0	0	0
QQ-enzymes-acylases-4	0	0	0	0	0
QQ-enzymes-acylases-5	9	2	3	16	12
QQ-enzymes-acylases-6	2	0	0	0	0
QQ-enzymes-acylases-7	0	0	0	0	0
QQ-enzymes-acylases-8	1	2	0	2	0
QQ-enzymes-acylases-9	0	0	0	0	0
QQ-enzymes-acylases-10	1	1	0	1	0
QQ-enzymes-acylases-11	11	1	5	12	5
QQ-enzymes-acylases-12	1	2	0	2	0
QQ-enzymes-acylases-13	0	1	0	0	0
QQ-enzymes-acylases-14	1	2	0	1	0
QQ-enzymes-acylases-15	2	3	0	5	0
QQ-enzymes-lactonases-1	27	8	13	20	17
QQ-enzymes-lactonases-6	2	0	2	0	0
QQ-enzymes-lactonases-7	0	0	0	0	0
QQ-enzymes-lactonases-8	4	1	2	1	1
QQ-enzymes-lactonases-9	0	0	0	0	0
QQ-enzymes-lactonases-13	3	1	2	0	1
QQ-enzymes-lactonases-14	20	6	9	19	13
QQ-enzymes-lactonases-15	0	0	1	0	0
QQ-enzymes-lactonases-16	1	1	0	0	2
QQ-enzymes-lactonases-18	4	0	1	1	2
QQ-enzymes-lactonases-19	1	0	0	0	2
QQ-enzymes-lactonases-20	40	8	14	23	28
QQ-enzymes-lactonases-24	1	0	0	1	1
QQ-enzymes-lactonases-25	128	53	59	172	106
QQ-enzymes-lactonases-26	4	3	4	6	3
QQ-enzymes-lactonases-27	1	1	0	1	0
QQ-enzymes-lactonases-28	3	2	3	3	2
DarABC-1	11	3	5	11	10
LsrR-1	11	2	2	7	3
LuxN-1	1	0	1	2	0
PqsABC-1	29	4	12	26	21
QscR-1	0	0	0	0	0
RpaR-1	2	0	0	1	1
	SRS023964	SRS024021	SRS024087	SRS024144	SRS024289
AHL-Receptors-4	1	2	1	1	1
AHL-Receptors-6	2	3	2	1	2
AHL-Receptors-7	2	3	2	1	2
AHL-Synthases-1	3	4	3	1	7
LuxS-1	12	8	21	14	15

LuxS-2	10	11	13	8	14
LuxS-3	13	11	14	11	16
LuxS-4	11	8	22	8	14
QQ-enzymes-acylases-1	7	5	13	3	9
QQ-enzymes-acylases-3	0	0	0	0	0
QQ-enzymes-acylases-4	0	0	0	0	0
QQ-enzymes-acylases-5	5	8	15	6	5
QQ-enzymes-acylases-6	0	0	1	0	0
QQ-enzymes-acylases-7	0	0	0	1	0
QQ-enzymes-acylases-8	1	0	1	0	1
QQ-enzymes-acylases-9	0	0	0	1	0
QQ-enzymes-acylases-10	0	0	1	0	0
QQ-enzymes-acylases-11	4	5	10	2	7
QQ-enzymes-acylases-12	1	0	1	0	1
QQ-enzymes-acylases-13	0	0	1	0	1
QQ-enzymes-acylases-14	0	0	2	1	1
QQ-enzymes-acylases-15	3	0	3	1	2
QQ-enzymes-lactonases-1	19	16	20	12	19
QQ-enzymes-lactonases-6	1	1	2	0	0
QQ-enzymes-lactonases-7	0	0	0	0	0
QQ-enzymes-lactonases-8	3	2	2	1	2
QQ-enzymes-lactonases-9	0	0	0	1	0
QQ-enzymes-lactonases-13	1	1	3	0	2
QQ-enzymes-lactonases-14	17	14	16	12	16
QQ-enzymes-lactonases-15	0	0	0	0	0
QQ-enzymes-lactonases-16	1	0	1	0	1
QQ-enzymes-lactonases-18	1	1	1	0	1
QQ-enzymes-lactonases-19	1	0	1	0	1
QQ-enzymes-lactonases-20	23	10	40	12	25
QQ-enzymes-lactonases-24	1	1	1	0	1
QQ-enzymes-lactonases-25	98	74	161	82	104
QQ-enzymes-lactonases-26	2	4	4	2	2
QQ-enzymes-lactonases-27	0	1	0	0	0
QQ-enzymes-lactonases-28	3	4	3	2	1
DarABC-1	7	4	11	8	8
LsrR-1	1	5	6	2	8
LuxN-1	3	0	2	0	0
PqsABC-1	15	14	35	18	24
QscR-1	0	0	0	1	0
RpaR-1	2	3	2	0	2

	SRS024355	SRS024381	SRS024447	SRS024561	SRS024649
AHL-Receptors-4	1	1	0	1	0
AHL-Receptors-6	3	2	1	2	0
AHL-Receptors-7	3	2	2	2	0
AHL-Synthases-1	5	3	3	4	3
LuxS-1	14	13	13	17	8
LuxS-2	12	16	15	15	8
LuxS-3	13	10	12	12	9

LuxS-4	13	11	9	16	15
QQ-enzymes-acylases-1	8	7	3	7	12
QQ-enzymes-acylases-3	0	0	0	0	0
QQ-enzymes-acylases-4	0	0	0	0	0
QQ-enzymes-acylases-5	13	7	7	8	10
QQ-enzymes-acylases-6	0	0	0	0	1
QQ-enzymes-acylases-7	0	0	0	0	0
QQ-enzymes-acylases-8	0	0	1	0	0
QQ-enzymes-acylases-9	0	0	0	0	0
QQ-enzymes-acylases-10	2	0	0	0	2
QQ-enzymes-acylases-11	5	6	2	7	9
QQ-enzymes-acylases-12	0	0	1	0	1
QQ-enzymes-acylases-13	0	0	0	0	0
QQ-enzymes-acylases-14	1	0	0	0	1
QQ-enzymes-acylases-15	3	1	1	0	2
QQ-enzymes-lactonases-1	19	13	13	15	9
QQ-enzymes-lactonases-6	2	2	0	1	2
QQ-enzymes-lactonases-7	0	0	0	0	0
QQ-enzymes-lactonases-8	1	3	0	3	1
QQ-enzymes-lactonases-9	0	0	0	0	0
QQ-enzymes-lactonases-13	0	2	0	1	0
QQ-enzymes-lactonases-14	16	10	11	13	9
QQ-enzymes-lactonases-15	0	0	0	0	0
QQ-enzymes-lactonases-16	0	0	1	1	0
QQ-enzymes-lactonases-18	2	1	2	0	0
QQ-enzymes-lactonases-19	1	1	1	0	0
QQ-enzymes-lactonases-20	24	30	14	25	21
QQ-enzymes-lactonases-24	1	1	0	1	0
QQ-enzymes-lactonases-25	116	83	81	108	105
QQ-enzymes-lactonases-26	3	3	2	3	1
QQ-enzymes-lactonases-27	0	1	1	0	0
QQ-enzymes-lactonases-28	3	2	2	2	1
DarABC-1	6	6	4	9	6
LsrR-1	5	4	6	3	6
LuxN-1	2	1	3	0	2
PqsABC-1	30	21	15	22	19
QscR-1	0	0	0	0	0
RpaR-1	3	2	1	2	0

	SRS042984	SRS043018	SRS043755	SRS043772	SRS045197
AHL-Receptors-4	4	0	0	0	1
AHL-Receptors-6	4	0	0	0	2
AHL-Receptors-7	5	0	0	0	2
AHL-Synthases-1	4	1	4	1	4
LuxS-1	18	4	9	9	28
LuxS-2	6	5	5	8	19
LuxS-3	11	9	9	10	20
LuxS-4	13	11	9	10	22
QQ-enzymes-acylases-1	8	6	6	2	13

QQ-enzymes-acylases-3	1	0	0	0	0
QQ-enzymes-acylases-4	0	0	0	0	0
QQ-enzymes-acylases-5	9	9	3	3	9
QQ-enzymes-acylases-6	1	0	0	0	0
QQ-enzymes-acylases-7	1	0	0	0	0
QQ-enzymes-acylases-8	1	1	0	0	1
QQ-enzymes-acylases-9	1	0	0	0	0
QQ-enzymes-acylases-10	1	0	0	0	1
QQ-enzymes-acylases-11	5	3	3	2	8
QQ-enzymes-acylases-12	1	0	0	0	0
QQ-enzymes-acylases-13	1	0	0	0	0
QQ-enzymes-acylases-14	0	0	0	0	0
QQ-enzymes-acylases-15	2	3	3	0	4
QQ-enzymes-lactonases-1	17	13	10	12	17
QQ-enzymes-lactonases-6	1	1	1	1	3
QQ-enzymes-lactonases-7	1	0	0	0	0
QQ-enzymes-lactonases-8	2	1	1	1	1
QQ-enzymes-lactonases-9	0	0	0	0	0
QQ-enzymes-lactonases-13	2	0	1	1	1
QQ-enzymes-lactonases-14	13	12	5	10	13
QQ-enzymes-lactonases-15	1	0	1	0	0
QQ-enzymes-lactonases-16	0	1	2	0	2
QQ-enzymes-lactonases-18	1	0	1	1	3
QQ-enzymes-lactonases-19	0	0	1	0	2
QQ-enzymes-lactonases-20	28	14	10	20	23
QQ-enzymes-lactonases-24	1	0	0	0	1
QQ-enzymes-lactonases-25	103	76	70	79	92
QQ-enzymes-lactonases-26	5	0	2	2	2
QQ-enzymes-lactonases-27	0	1	0	0	0
QQ-enzymes-lactonases-28	5	2	1	2	2
DarABC-1	11	7	7	5	13
LsrR-1	4	3	2	1	5
LuxN-1	1	0	2	0	1
PqsABC-1	28	14	10	7	26
QscR-1	1	0	0	0	0
RpaR-1	4	0	0	0	2

	SRS045313	SRS047100	SRS047113	SRS047265	SRS047634
AHL-Receptors-4	0	1	0	0	2
AHL-Receptors-6	0	2	2	0	2
AHL-Receptors-7	0	2	1	1	3
AHL-Synthases-1	0	5	3	2	7
LuxS-1	4	7	15	5	19
LuxS-2	4	6	11	6	14
LuxS-3	4	9	13	6	14
LuxS-4	9	2	18	9	33
QQ-enzymes-acylases-1	3	0	6	2	16
QQ-enzymes-acylases-3	0	0	0	0	0
QQ-enzymes-acylases-4	0	0	0	0	0

QQ-enzymes-acylases-5	1	7	9	1	20
QQ-enzymes-acylases-6	0	0	1	0	1
QQ-enzymes-acylases-7	0	0	2	0	0
QQ-enzymes-acylases-8	0	0	2	0	1
QQ-enzymes-acylases-9	0	0	2	0	0
QQ-enzymes-acylases-10	0	0	0	0	0
QQ-enzymes-acylases-11	3	0	3	2	13
QQ-enzymes-acylases-12	0	0	2	0	1
QQ-enzymes-acylases-13	0	0	1	0	1
QQ-enzymes-acylases-14	0	0	1	0	1
QQ-enzymes-acylases-15	0	0	1	0	3
QQ-enzymes-lactonases-1	6	5	13	9	21
QQ-enzymes-lactonases-6	0	1	1	2	0
QQ-enzymes-lactonases-7	0	0	0	0	0
QQ-enzymes-lactonases-8	0	1	2	0	0
QQ-enzymes-lactonases-9	0	0	0	0	0
QQ-enzymes-lactonases-13	0	1	0	0	0
QQ-enzymes-lactonases-14	6	4	12	8	19
QQ-enzymes-lactonases-15	0	0	0	1	0
QQ-enzymes-lactonases-16	0	0	0	0	1
QQ-enzymes-lactonases-18	0	0	1	0	2
QQ-enzymes-lactonases-19	0	0	0	0	1
QQ-enzymes-lactonases-20	5	9	24	3	40
QQ-enzymes-lactonases-24	0	1	1	0	1
QQ-enzymes-lactonases-25	33	56	132	44	130
QQ-enzymes-lactonases-26	1	2	4	1	3
QQ-enzymes-lactonases-27	0	0	0	1	1
QQ-enzymes-lactonases-28	0	1	4	0	4
DarABC-1	1	3	14	2	15
LsrR-1	0	4	8	1	8
LuxN-1	0	1	2	1	1
PqsABC-1	6	7	25	4	34
QscR-1	0	0	1	0	0
RpaR-1	0	2	1	0	2

	SRS049268	SRS049318	SRS051244	SRS051378	SRS051930
AHL-Receptors-4	0	1	1	0	2
AHL-Receptors-6	0	0	0	0	3
AHL-Receptors-7	1	2	2	0	4
AHL-Synthases-1	4	5	5	3	3
LuxS-1	17	17	12	9	15
LuxS-2	11	12	14	5	17
LuxS-3	11	10	11	7	15
LuxS-4	21	30	10	6	10
QQ-enzymes-acylases-1	9	14	7	4	11
QQ-enzymes-acylases-3	0	0	0	0	0
QQ-enzymes-acylases-4	0	0	0	0	0
QQ-enzymes-acylases-5	10	10	5	5	13
QQ-enzymes-acylases-6	1	1	0	0	0

QQ-enzymes-acylases-7	0	0	0	0	0
QQ-enzymes-acylases-8	2	3	0	0	1
QQ-enzymes-acylases-9	0	0	0	0	0
QQ-enzymes-acylases-10	1	0	2	1	3
QQ-enzymes-acylases-11	5	10	4	3	6
QQ-enzymes-acylases-12	2	3	1	0	1
QQ-enzymes-acylases-13	0	0	0	0	0
QQ-enzymes-acylases-14	0	1	1	1	3
QQ-enzymes-acylases-15	3	4	2	1	4
QQ-enzymes-lactonases-1	13	12	12	13	10
QQ-enzymes-lactonases-6	2	1	1	1	0
QQ-enzymes-lactonases-7	1	0	0	0	0
QQ-enzymes-lactonases-8	0	3	1	1	2
QQ-enzymes-lactonases-9	0	1	0	0	1
QQ-enzymes-lactonases-13	0	2	0	0	1
QQ-enzymes-lactonases-14	12	8	10	12	8
QQ-enzymes-lactonases-15	0	0	0	0	0
QQ-enzymes-lactonases-16	1	2	1	1	0
QQ-enzymes-lactonases-18	0	2	1	1	1
QQ-enzymes-lactonases-19	0	2	1	1	1
QQ-enzymes-lactonases-20	26	32	17	12	24
QQ-enzymes-lactonases-24	0	1	0	0	1
QQ-enzymes-lactonases-25	128	136	90	63	109
QQ-enzymes-lactonases-26	3	2	1	1	4
QQ-enzymes-lactonases-27	0	0	0	1	0
QQ-enzymes-lactonases-28	4	3	1	1	3
DarABC-1	6	8	3	4	7
LsrR-1	7	2	2	1	5
LuxN-1	2	2	0	0	0
PqsABC-1	23	27	15	11	22
QscR-1	0	0	0	0	0
RpaR-1	0	0	0	0	3

	SRS051941	SRS052604	SRS052876	SRS053584	SRS054430
AHL-Receptors-4	1	0	0	1	1
AHL-Receptors-6	2	0	0	1	2
AHL-Receptors-7	1	0	0	1	2
AHL-Synthases-1	3	2	3	4	4
LuxS-1	9	5	18	9	13
LuxS-2	8	4	18	10	9
LuxS-3	10	4	13	12	6
LuxS-4	8	8	11	4	8
QQ-enzymes-acylases-1	3	2	12	7	6
QQ-enzymes-acylases-3	0	0	0	0	0
QQ-enzymes-acylases-4	0	0	0	0	0
QQ-enzymes-acylases-5	8	2	15	4	3
QQ-enzymes-acylases-6	0	0	0	0	0
QQ-enzymes-acylases-7	0	0	0	0	0
QQ-enzymes-acylases-8	0	0	3	0	0

QQ-enzymes-acylases-9	0	0	0	0	0
QQ-enzymes-acylases-10	0	0	0	1	2
QQ-enzymes-acylases-11	2	2	8	4	4
QQ-enzymes-acylases-12	0	0	2	0	0
QQ-enzymes-acylases-13	0	0	0	0	0
QQ-enzymes-acylases-14	0	0	0	0	1
QQ-enzymes-acylases-15	1	0	4	3	1
QQ-enzymes-lactonases-1	11	11	15	9	11
QQ-enzymes-lactonases-6	2	0	0	1	2
QQ-enzymes-lactonases-7	0	0	0	0	0
QQ-enzymes-lactonases-8	1	1	1	1	2
QQ-enzymes-lactonases-9	0	0	0	1	0
QQ-enzymes-lactonases-13	0	1	1	1	1
QQ-enzymes-lactonases-14	9	8	10	8	9
QQ-enzymes-lactonases-15	0	0	0	0	1
QQ-enzymes-lactonases-16	1	0	1	0	0
QQ-enzymes-lactonases-18	1	2	3	0	0
QQ-enzymes-lactonases-19	2	0	0	0	0
QQ-enzymes-lactonases-20	25	15	23	13	12
QQ-enzymes-lactonases-24	1	0	0	1	1
QQ-enzymes-lactonases-25	78	51	98	61	64
QQ-enzymes-lactonases-26	1	1	2	2	2
QQ-enzymes-lactonases-27	0	0	0	1	0
QQ-enzymes-lactonases-28	2	1	2	2	2
DarABC-1	7	4	9	9	9
LsrR-1	3	2	2	3	1
LuxN-1	3	1	2	0	1
PqsABC-1	21	10	20	15	7
QscR-1	0	0	0	0	0
RpaR-1	1	0	0	1	2
	SRS054653	SRS055378	SRS055401	SRS055450	SRS058053
AHL-Receptors-4	2	0	1	0	1
AHL-Receptors-6	2	0	1	0	2
AHL-Receptors-7	4	0	1	0	2
AHL-Synthases-1	2	5	5	0	5
LuxS-1	6	11	14	9	11
LuxS-2	7	7	12	6	4
LuxS-3	9	8	15	6	11
LuxS-4	1	13	6	6	10
QQ-enzymes-acylases-1	4	13	4	6	7
QQ-enzymes-acylases-3	0	0	0	0	0
QQ-enzymes-acylases-4	0	0	0	0	0
QQ-enzymes-acylases-5	3	12	3	6	6
QQ-enzymes-acylases-6	0	1	0	0	0
QQ-enzymes-acylases-7	0	0	0	0	0
QQ-enzymes-acylases-8	0	1	0	1	0
QQ-enzymes-acylases-9	0	0	0	0	0
QQ-enzymes-acylases-10	1	0	0	0	2

QQ-enzymes-acylases-11	1	10	4	5	5
QQ-enzymes-acylases-12	0	1	0	0	0
QQ-enzymes-acylases-13	0	0	0	0	0
QQ-enzymes-acylases-14	0	0	0	0	1
QQ-enzymes-acylases-15	2	3	0	1	1
QQ-enzymes-lactonases-1	12	16	9	12	12
QQ-enzymes-lactonases-6	0	0	1	0	1
QQ-enzymes-lactonases-7	0	0	0	0	0
QQ-enzymes-lactonases-8	0	1	2	1	2
QQ-enzymes-lactonases-9	0	0	0	0	0
QQ-enzymes-lactonases-13	0	1	1	1	2
QQ-enzymes-lactonases-14	11	14	7	9	9
QQ-enzymes-lactonases-15	0	0	0	0	1
QQ-enzymes-lactonases-16	0	0	0	1	0
QQ-enzymes-lactonases-18	1	1	1	2	0
QQ-enzymes-lactonases-19	0	0	1	1	0
QQ-enzymes-lactonases-20	8	30	15	17	18
QQ-enzymes-lactonases-24	1	1	1	0	1
QQ-enzymes-lactonases-25	44	130	83	65	118
QQ-enzymes-lactonases-26	1	3	2	1	2
QQ-enzymes-lactonases-27	1	1	0	0	0
QQ-enzymes-lactonases-28	1	3	1	1	3
DarABC-1	2	10	5	5	5
LsrR-1	2	9	3	0	3
LuxN-1	2	1	1	1	1
PqsABC-1	7	21	10	8	18
QscR-1	0	0	0	0	0
RpaR-1	2	0	0	0	2

	SRS058808	SRS063603	SRS063999	SRS064449	SRS064493
AHL-Receptors-4	0	0	0	1	0
AHL-Receptors-6	0	0	0	0	0
AHL-Receptors-7	0	0	0	1	0
AHL-Synthases-1	3	2	5	7	0
LuxS-1	20	17	20	12	3
LuxS-2	16	15	9	12	3
LuxS-3	13	12	15	14	5
LuxS-4	30	25	21	16	2
QQ-enzymes-acylases-1	9	11	12	6	4
QQ-enzymes-acylases-3	0	0	0	0	0
QQ-enzymes-acylases-4	0	0	0	0	0
QQ-enzymes-acylases-5	11	15	7	2	3
QQ-enzymes-acylases-6	0	0	1	0	1
QQ-enzymes-acylases-7	0	0	0	0	0
QQ-enzymes-acylases-8	0	0	1	0	2
QQ-enzymes-acylases-9	0	0	0	0	0
QQ-enzymes-acylases-10	0	1	0	1	0
QQ-enzymes-acylases-11	9	8	10	4	2
QQ-enzymes-acylases-12	0	0	1	0	2

QQ-enzymes-acylases-13	0	0	0	0	0
QQ-enzymes-acylases-14	0	0	2	0	1
QQ-enzymes-acylases-15	0	2	2	2	2
QQ-enzymes-lactonases-1	18	21	20	16	6
QQ-enzymes-lactonases-6	3	1	2	1	2
QQ-enzymes-lactonases-7	0	0	0	0	0
QQ-enzymes-lactonases-8	3	2	0	2	0
QQ-enzymes-lactonases-9	0	1	0	0	0
QQ-enzymes-lactonases-13	0	2	1	1	0
QQ-enzymes-lactonases-14	16	16	17	12	6
QQ-enzymes-lactonases-15	0	0	0	0	0
QQ-enzymes-lactonases-16	1	1	2	1	0
QQ-enzymes-lactonases-18	1	3	2	3	0
QQ-enzymes-lactonases-19	0	2	2	2	0
QQ-enzymes-lactonases-20	34	32	26	14	0
QQ-enzymes-lactonases-24	0	0	0	1	0
QQ-enzymes-lactonases-25	104	123	111	81	24
QQ-enzymes-lactonases-26	3	3	2	3	1
QQ-enzymes-lactonases-27	0	0	0	0	0
QQ-enzymes-lactonases-28	1	2	2	3	0
DarABC-1	8	9	10	12	1
LsrR-1	6	4	8	3	1
LuxN-1	4	0	1	0	0
PqsABC-1	15	28	25	18	2
QscR-1	0	0	0	0	0
RpaR-1	0	0	0	0	0

	SRS065099	SRS065310	SRS075410	subgingival
AHL-Receptors-4	1	0	2	1
AHL-Receptors-6	1	0	6	2
AHL-Receptors-7	2	0	4	2
AHL-Synthases-1	4	1	10	2
LuxS-1	16	4	10	9
LuxS-2	20	5	9	9
LuxS-3	16	8	10	12
LuxS-4	20	6	4	8
QQ-enzymes-acylases-1	8	1	7	5
QQ-enzymes-acylases-3	0	0	1	0
QQ-enzymes-acylases-4	0	0	0	0
QQ-enzymes-acylases-5	5	1	6	8
QQ-enzymes-acylases-6	1	0	0	0
QQ-enzymes-acylases-7	0	0	1	0
QQ-enzymes-acylases-8	2	0	0	0
QQ-enzymes-acylases-9	0	0	1	0
QQ-enzymes-acylases-10	0	1	0	0
QQ-enzymes-acylases-11	6	0	4	5
QQ-enzymes-acylases-12	1	0	0	0
QQ-enzymes-acylases-13	0	0	0	0
QQ-enzymes-acylases-14	1	0	0	0

QQ-enzymes-acylases-15	2	1	2	0
QQ-enzymes-lactonases-1	18	7	16	18
QQ-enzymes-lactonases-6	1	1	0	1
QQ-enzymes-lactonases-7	0	0	0	0
QQ-enzymes-lactonases-8	2	0	1	3
QQ-enzymes-lactonases-9	0	0	0	0
QQ-enzymes-lactonases-13	1	0	0	3
QQ-enzymes-lactonases-14	15	5	14	15
QQ-enzymes-lactonases-15	0	0	0	0
QQ-enzymes-lactonases-16	0	1	0	0
QQ-enzymes-lactonases-18	2	2	2	0
QQ-enzymes-lactonases-19	2	1	1	0
QQ-enzymes-lactonases-20	27	12	13	17
QQ-enzymes-lactonases-24	1	0	3	1
QQ-enzymes-lactonases-25	101	60	69	18
QQ-enzymes-lactonases-26	2	0	5	3
QQ-enzymes-lactonases-27	1	0	0	0
QQ-enzymes-lactonases-28	1	0	5	3
DarABC-1	10	3	9	2
LsrR-1	5	2	4	3
LuxN-1	2	0	0	0
PqsABC-1	20	4	20	13
QscR-1	0	0	1	0
RpaR-1	1	0	3	2

[8]:

```
#normalizing by predicted proteins for each metagenome from metagenome_norm.txt
vecnorm <-␣
 ↪c(6266,8924,11539,15784,3843,146008,13004,120288,279009,207088,208177,172270,231720,310688,
           ␣
 ↪258578,260380,193617,174046,210877,194510,123961,127734,195986,104185,139734,201207,159066,
           ␣
 ↪161080,161822,248646,75099,113543,160533,129723,74250,112845,173751,85615,131008,204627,157
           ␣
 ↪268038,207912,113886,241990,371149,127446,193398,230069,315002,84406,264435,91803,211906,14
           ␣
 ↪242200,39469,152094,203222,177345,226406,97304,72535,106559,118180,135169,219548,244747,145
           ␣
 ↪203619,119634,109931,162510,325852,315060,110055,104667,390735,194224,174843,122968,304260,
           ␣
 ↪185172,256145,165500,181019,217294,195067,228964,202893,132003,118570,253977,67257,110290,3
           ␣
 ↪68465,344851,235839,389276,193439,107331,212768,211521,107010,188371,116293,123826,93368,28
           ␣
 ↪137758,130514,241830,240205,304829,278640,216545,39161,230667,85864,136029,46349)
divnorm <- sweep(otu,2,vecnorm,'/') #2 is for column operation

OTU = otu_table(otu, taxa_are_rows=T) # raw OTU table
```

```r
OTUn = otu_table(divnorm, taxa_are_rows=T) #normalized OTU table
taximat <- as.matrix(read.table("taxonomy2023.txt", header=T, row.names=1,␣
↪sep="\t"))
taxi = tax_table(taximat)
quorum <- phyloseq(OTU, taxi)
data = read.table("samples.txt", header=T, row.names=1, sep="\t")
sampledata = sample_data(data.frame(id=data$id, project=data$project, row.names␣
↪= sample_names(quorum)))
quorum = phyloseq (OTU, sampledata, taxi) #raw OTU object
quorumN =phyloseq (OTUn, sampledata, taxi) #normalized

tax_table(quorumN)
```

A taxonomyTable: 43 × 6 of type chr

	L0	L1
AHL-Receptors-4	AHL-Receptors-4-SdiA	AHL-Receptor
AHL-Receptors-6	AHL-Receptors-6-RhlR	AHL-Receptor
AHL-Receptors-7	AHL-Receptors-7-AbaR	AHL-Receptor
AHL-Synthases-1	AHL-Synthases-1-HdtS	AHL-Synthase
LuxS-1	LuxS-1-V._harveyi	LuxS
LuxS-2	LuxS-2-S._pyogenes	LuxS
LuxS-3	LuxS-3-B._thuringiensis	LuxS
LuxS-4	LuxS-4-B._heparinolyticus	LuxS
QQ-enzymes-acylases-1	QQ-acylases-1-Aac	QQ-enzymes-ac
QQ-enzymes-acylases-3	QQ-acylases-3-PvdQ	QQ-enzymes-ac
QQ-enzymes-acylases-4	QQ-acylases-4-QuiP-QlcA	QQ-enzymes-ac
QQ-enzymes-acylases-5	QQ-acylases-5-PheA	QQ-enzymes-ac
QQ-enzymes-acylases-6	QQ-acylases-6-HacB	QQ-enzymes-ac
QQ-enzymes-acylases-7	QQ-acylases-7-KcPGA	QQ-enzymes-ac
QQ-enzymes-acylases-8	QQ-acylases-8-Aibp	QQ-enzymes-ac
QQ-enzymes-acylases-9	QQ-acylases-9-AiiC	QQ-enzymes-ac
QQ-enzymes-acylases-10	QQ-acylases-10-AhlA	QQ-enzymes-ac
QQ-enzymes-acylases-11	QQ-acylases-11-AhlM	QQ-enzymes-ac
QQ-enzymes-acylases-12	QQ-acylases-12-AiiO	QQ-enzymes-ac
QQ-enzymes-acylases-13	QQ-acylases-13-AmiE	QQ-enzymes-ac
QQ-enzymes-acylases-14	QQ-acylases-14-APTM01	QQ-enzymes-ac
QQ-enzymes-acylases-15	QQ-acylases-15-MacQ	QQ-enzymes-ac
QQ-enzymes-lactonases-1	QQ-lactonases-1-AidA	QQ-enzymes-la
QQ-enzymes-lactonases-6	QQ-lactonases-6-CarA/CarB	QQ-enzymes-la
QQ-enzymes-lactonases-7	QQ-lactonases-7-RmmL	QQ-enzymes-la
QQ-enzymes-lactonases-8	QQ-lactonases-8-Y2-AiiA	QQ-enzymes-la
QQ-enzymes-lactonases-9	QQ-lactonases-9-YtnP	QQ-enzymes-la
QQ-enzymes-lactonases-13	QQ-lactonases-13-AhlD	QQ-enzymes-la
QQ-enzymes-lactonases-14	QQ-lactonases-14-AhlK	QQ-enzymes-la
QQ-enzymes-lactonases-15	QQ-lactonases-15-AhlS	QQ-enzymes-la
QQ-enzymes-lactonases-16	QQ-lactonases-16-AidC	QQ-enzymes-la
QQ-enzymes-lactonases-18	QQ-lactonases-18-mlr6805	QQ-enzymes-la
QQ-enzymes-lactonases-20	QQ-lactonases-20-QsdR1	QQ-enzymes-la
QQ-enzymes-lactonases-24	QQ-paraoxonases-PON	QQ-enzymes-la
QQ-enzymes-lactonases-25	QQ-lactonases-25-GsP	QQ-enzymes-la
QQ-enzymes-lactonases-26	QQ-lactonases-26-MCP	QQ-enzymes-la
QQ-enzymes-lactonases-27	QQ-lactonases-27-QsdH	QQ-enzymes-la
QQ-enzymes-lactonases-28	QQ-lactonases-28-Bpi01	QQ-enzymes-la
LsrR-1	AI-2-Receptors-1-LsrR	LsrR-1
LuxN-1	AHL-Receptors-10-LuxN	LuxN-1
PqsABC-1	PQS-Synthase-PqsABC	PqsABC-1
QscR-1	Non-AHL-Receptors-1-QscR	QscR-1
RpaR-1	Non-AHL-Receptors-2-RpaR	RpaR-1

[26]:
```
p <- plot_bar(quorumN,"L0")
p
```

```
#
data <- psmelt(quorumN)
head(data)
data$L0 <- as.character(data$L0)

# install.packages("tidyverse")
library("tidyverse")

pd <- data %>%
    as_tibble %>%
    mutate(L0 = as.character(L0)) # %>%
#     replace_na(list(Genus = "unknown"))
genus_abun <- pd %>%
    group_by(L0) %>%
    summarize(Abundance = sum(Abundance)) %>%
    arrange(Abundance)
genus_levels <- genus_abun$L0
pd0 <- pd %>%
    mutate(L0 = factor(L0, genus_levels))

print(pd0)
z <- factor(unique(data$L0))
z <- nlevels(z)

head(pd0)
# p <- ggplot(pd0, aes(x = Sample, y = Abundance, fill= pd0$L1, color= pd0$L1))␣
↪+ geom_bar(stat = "identity", position="stack")  +
#                                                ␣
↪scale_fill_manual(values=rev(colorRampPalette(brewer.pal(12,"Paired"))(z))) +
#                                                ␣
↪scale_color_manual(values=rev(colorRampPalette(brewer.pal(12,"Paired"))(z)))

# p
p2 <- ggplot(pd0, aes(x = L0, y = Sample, fill= Abundance)) + ␣
↪scale_fill_gradient(low="#B9DDF1", high="#2A5783", na.value = "white", trans␣
↪= "log2") + geom_tile() + coord_flip() +  theme(axis.text.x = element_text(␣
↪size=3, angle = 90, hjust = 1), axis.text.y = element_text(size = 6)) +␣
↪labs(y = "Metagenome", x="")
# p2 <- ggplot(pd0, aes(x = L0, y = Sample, fill= Abundance)) + ␣
↪scale_fill_gradient(low="#bebebe", high="#000000", na.value = "white", trans␣
↪= "log2") + geom_tile() + coord_flip() +  theme(axis.text.x = element_text(␣
↪size=4, angle = 90, hjust = 1), axis.text.y = element_text(size = 6)) +␣
↪labs(y = "Metagenome", x="")
```

```r
# p2 <- ggplot(pd0, aes(x = L0, y = Sample, fill= Abundance)) + ␣
↪scale_fill_gradient(low="#B9DDF1", high="#2A5783", na.value = "white", trans␣
↪= "log2") + geom_tile() + coord_flip() +  theme(axis.text.x = element_text(␣
↪size=2, angle = 90, hjust = 1), axis.text.y = element_text(size = 6)) +␣
↪labs(y = "Metagenome", x="")

p2

ggsave("Quorum_heatmap.pdf", , width=21, height=27, units="cm")
```

A data.frame: 6 × 11

	OTU <chr>	Sample <chr>	Abundance <dbl>	id <chr>	project <chr>	I[illegible]
4173	QQ-enzymes-lactonases-25	NCA008	0.0015612802	NCA008	Spain	[illegible]
4203	QQ-enzymes-lactonases-25	NCA001	0.0011205737	NCA001	Spain	[illegible]
4185	QQ-enzymes-lactonases-25	NOCA002P	0.0011163772	NOCA002P	Spain	[illegible]
4182	QQ-enzymes-lactonases-25	NCA004	0.0009503294	NCA004	Spain	[illegible]
4189	QQ-enzymes-lactonases-25	RL7Ale	0.0009227930	RL7Ale	Spain	[illegible]
4164	QQ-enzymes-lactonases-25	SRS065310	0.0006987795	SRS065310	HMP	[illegible]

```
# A tibble: 5,418 × 11
   OTU          Sample Abund…¹ id     project L0     L1     L2     L3     L4     L5
   <chr>        <chr>
<dbl> <chr> <chr>
<fct> <chr> <chr>
<chr> <chr> <chr>
 1 QQ-enzymes-… NCA008 1.56e-3 NCAO… Spain   QQ-l…
QQ-e… Pseu… pdb|… pdb.… NA
 2 QQ-enzymes-… NCA001 1.12e-3 NCAO… Spain   QQ-l…
QQ-e… Pseu… pdb|… pdb.… NA
 3 QQ-enzymes-… NOCAO… 1.12e-3 NOCA… Spain   QQ-l…
QQ-e… Pseu… pdb|… pdb.… NA
 4 QQ-enzymes-… NCA004 9.50e-4 NCAO… Spain   QQ-l…
QQ-e… Pseu… pdb|… pdb.… NA
 5 QQ-enzymes-… RL7Ale 9.23e-4 RL7A… Spain   QQ-l…
QQ-e… Pseu… pdb|… pdb.… NA
 6 QQ-enzymes-… SRS06… 6.99e-4 SRSO… HMP     QQ-l…
QQ-e… Pseu… pdb|… pdb.… NA
 7 QQ-enzymes-… SRS01… 6.92e-4 SRSO… HMP     QQ-l…
QQ-e… Pseu… pdb|… pdb.… NA
 8 QQ-enzymes-… SRS02… 6.92e-4 SRSO… HMP     QQ-l…
QQ-e… Pseu… pdb|… pdb.… NA
 9 QQ-enzymes-… SRS01… 6.89e-4 SRSO… HMP     QQ-l…
QQ-e… Pseu… pdb|… pdb.… NA
10 QQ-enzymes-… SRS01… 6.82e-4 SRSO… HMP     QQ-l…
QQ-e… Pseu… pdb|… pdb.… NA
# … with 5,408 more rows, and abbreviated variable name ¹Abundance
```

A tibble: 6 × 11

OTU	Sample	Abundance	id	project	L0
<chr>	<chr>	<dbl>	<chr>	<chr>	<fct>
QQ-enzymes-lactonases-25	NCA008	0.0015612802	NCA008	Spain	QQ-lactonase
QQ-enzymes-lactonases-25	NCA001	0.0011205737	NCA001	Spain	QQ-lactonase
QQ-enzymes-lactonases-25	NOCA002P	0.0011163772	NOCA002P	Spain	QQ-lactonase
QQ-enzymes-lactonases-25	NCA004	0.0009503294	NCA004	Spain	QQ-lactonase
QQ-enzymes-lactonases-25	RL7Ale	0.0009227930	RL7Ale	Spain	QQ-lactonase
QQ-enzymes-lactonases-25	SRS065310	0.0006987795	SRS065310	HMP	QQ-lactonase

```
Warning message:
"Transformation introduced infinite values in discrete y-axis"
```

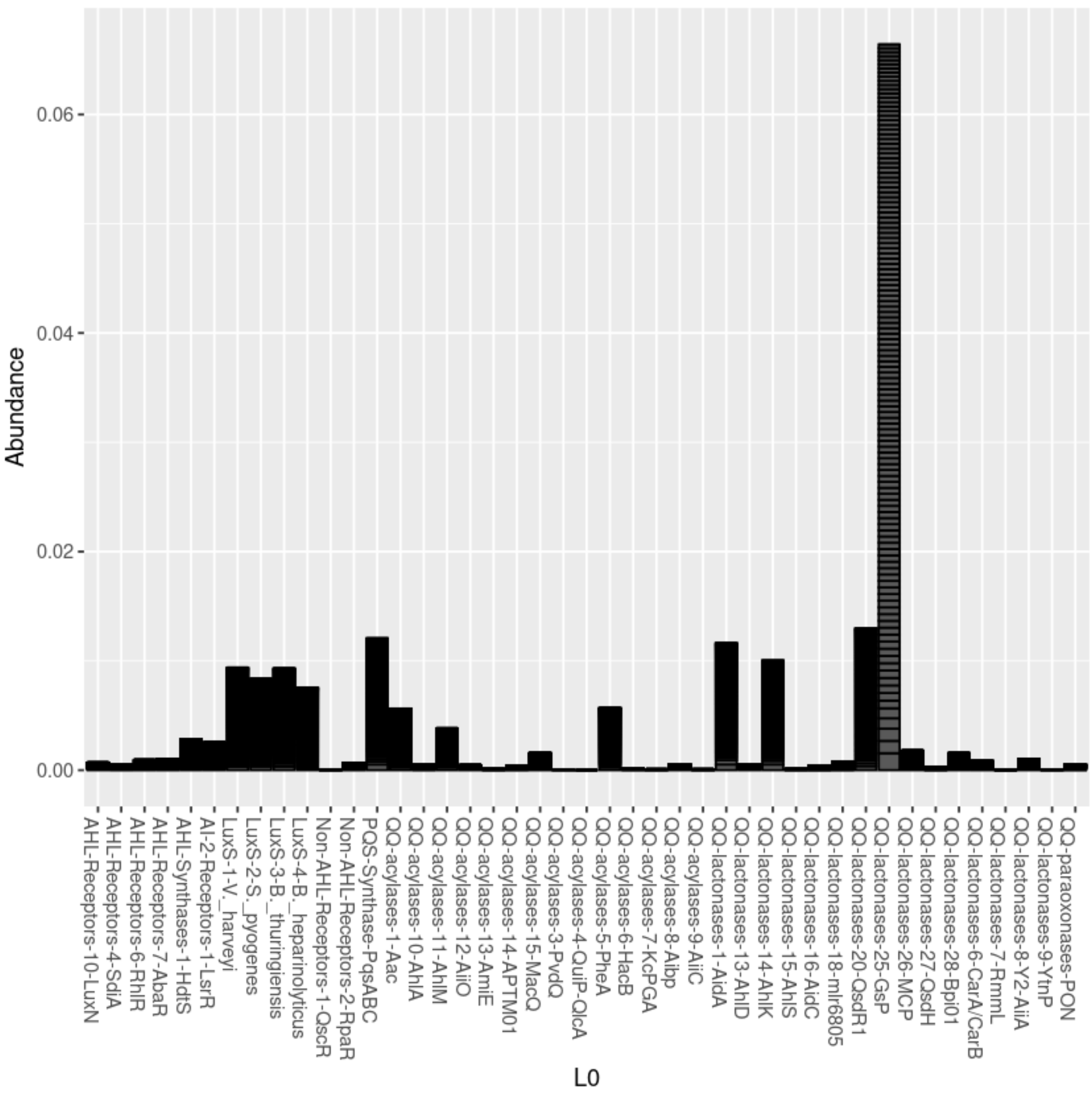


```
Warning message:
"Transformation introduced infinite values in discrete y-axis"
```

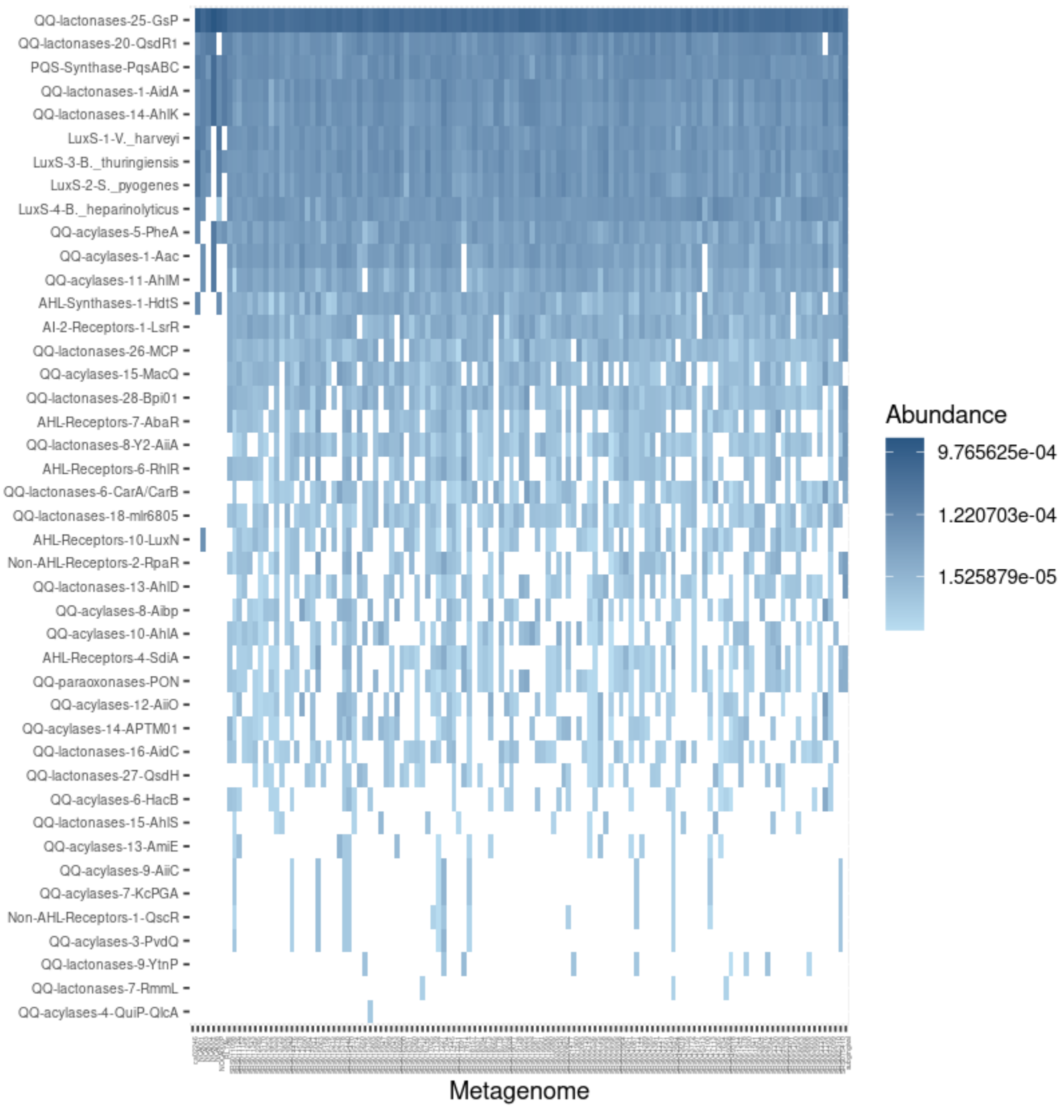
QQ-lactonases-25-GsP
QQ-lactonases-20-QsdR1
PQS-Synthase-PqsABC
QQ-lactonases-1-AidA
QQ-lactonases-14-AhlK
LuxS-1-V._harveyi
LuxS-3-B._thuringiensis
LuxS-2-S._pyogenes
LuxS-4-B._heparinolyticus
QQ-acylases-5-PheA
QQ-acylases-1-Aac
QQ-acylases-11-AhlM
AHL-Synthases-1-HdtS
AI-2-Receptors-1-LsrR
QQ-lactonases-26-MCP
QQ-acylases-15-MacQ
QQ-lactonases-28-Bpi01
AHL-Receptors-7-AbaR
QQ-lactonases-8-Y2-AiiA
AHL-Receptors-6-RhlR
QQ-lactonases-6-CarA/CarB
QQ-lactonases-18-mlr6805
AHL-Receptors-10-LuxN
Non-AHL-Receptors-2-RpaR
QQ-lactonases-13-AhlD
QQ-acylases-8-Aibp
QQ-acylases-10-AhlA
AHL-Receptors-4-SdiA
QQ-paraoxonases-PON
QQ-acylases-12-AiiO
QQ-acylases-14-APTM01
QQ-lactonases-16-AidC
QQ-lactonases-27-QsdH
QQ-acylases-6-HacB
QQ-lactonases-15-AhlS
QQ-acylases-13-AmiE
QQ-acylases-9-AiiC
QQ-acylases-7-KcPGA
Non-AHL-Receptors-1-QscR
QQ-acylases-3-PvdQ
QQ-lactonases-9-YtnP
QQ-lactonases-7-RmmL
QQ-acylases-4-QuiP-QlcA
Abundance
9.765625e-04
1.220703e-04
1.525879e-05
Metagenome

[ ]:

2 References

Nadkarni, M. A., Martin, F. E., Jacques, N. A., and Hunter, N. (2002). Determination of bacterial load by real-time PCR using a broad-range (universal) probe and primers set. *Microbiology* 148, 257–266. doi: 10.1099/00221287-148-1-257.

Papapanou, P. N., Sanz, M., Buduneli, N., Dietrich, T., Feres, M., Fine, D. H., et al. (2018). Periodontitis: Consensus report of workgroup 2 of the 2017 World Workshop on the Classification of Periodontal and Peri-Implant Diseases and Conditions. *J. Clin. Periodontol.* 45, S162–S170. doi: 10.1111/jcpe.12946.

Tonetti, M. S., Greenwell, H., and Kornman, K. S. (2018). Staging and grading of periodontitis: Framework and proposal of a new classification and case definition. *J. Periodontol.* 89, S159–S172. doi: 10.1002/JPER.18-0006.

Vilarrasa, J., Delgado, L. M., Galofré, M., Àlvarez, G., Violant, D., Manero, J. M., et al. (2018). In vitro evaluation of a multispecies oral biofilm over antibacterial coated titanium surfaces. *J. Mater. Sci. Mater. Med.* 29, 164. doi: 10.1007/s10856-018-6168-8.
